# Investigation of Structure, Ionic Conductivity, and Electrochemical Stability of Halogen Substitution in Solid-State Ion Conductor Li_3_YBr_*x*_Cl_6–*x*_

**DOI:** 10.1021/acs.jpcc.2c07910

**Published:** 2022-12-16

**Authors:** Eveline van der Maas, Wenxuan Zhao, Zhu Cheng, Theodosios Famprikis, Michel Thijs, Steven R. Parnell, Swapna Ganapathy, Marnix Wagemaker

**Affiliations:** Department of Radiation Science and Technology, Faculty of Applied Sciences, Delft University of Technology, 2629JBDelft, The Netherlands

## Abstract

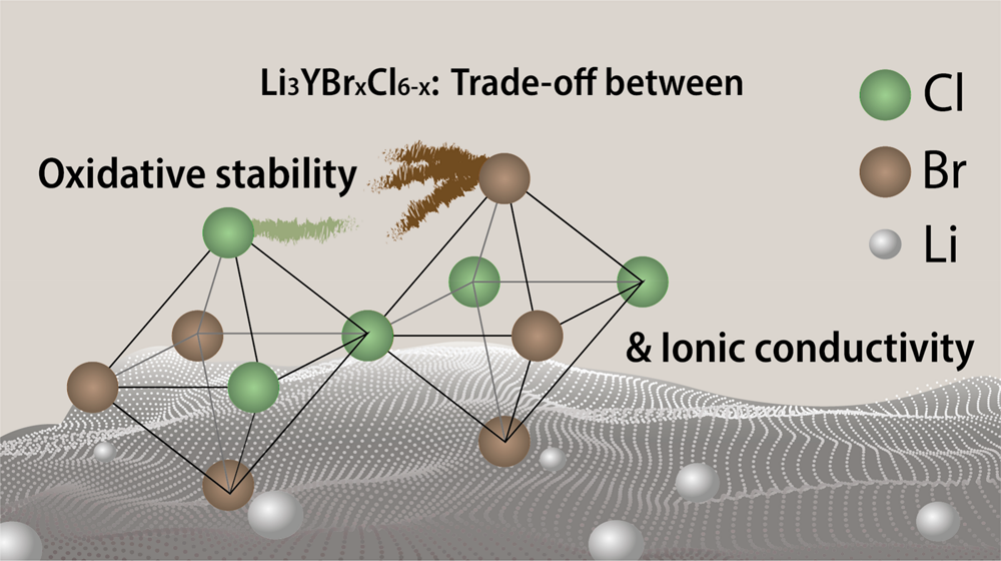

Li_3_YX_6_ (X = Cl, Br) materials are Li-ion conductors that can be used as solid electrolytes in all solid-state batteries. Solid electrolytes ideally have high ionic conductivity and (electro)chemical compatibility with the electrodes. It was proven that introducing Br to Li_3_YCl_6_ increases ionic conductivity but, according to thermodynamic calculations, should also reduce oxidative stability. In this paper, the trade-off between ionic conductivity and electrochemical stability in Li_3_YBr_*x*_Cl_6–*x*_ halogen-substituted compounds is investigated. The compositions of Li_3_YBr_1.5_Cl_4.5_ and Li_3_YBr_4.5_Cl_1.5_ are reported for the first time, along with a consistent analysis of the whole Li_3_YBr_*x*_Cl_6–*x*_ (*x* = 0–6) tie-line. The results show that, while Br-rich materials are more conductive (5.36 × 10^–3^ S/cm at 30 °C for *x* = 4.5), the oxidative stability is lower (∼3 V compared to ∼3.5 V). Small Br content (*x* = 1.5) does not affect oxidative stability but substantially increases ionic conductivity compared to pristine Li_3_YCl_6_ (2.1 compared to 0.049 × 10^–3^ S/cm at 30 °C). This work highlights that optimization of substitutions in the anion framework provide prolific and rational avenues for tailoring the properties of solid electrolytes.

## Introduction

Solid-state electrolytes for all-solid-state batteries (ASSBs) have been intensively researched in recent years, due to the improved safety they offer compared to liquid electrolytes and because they may enable alternative electrodes.^1^ In 2018, Asano et al.^2^ reported that the ternary halides Li_3_YCl_6_ and Li_3_YBr_6_ have ionic conductivities of ∼1 × 10^–3^ S/cm and showcased ASSBs with Coulombic efficiencies of 94% using (uncoated) LiCoO_2_ as the active material in the cathode composite. Since then, the material family Li_3_M(III)X_6_ (M(III) = Y, In, Sc, lanthanides; X = Cl, Br, I)) and other halide solid electrolytes have gained renewed interest in the scientific community due to their favorable combination of ionic conductivity (∼mS/cm) and high-voltage cathode compatibility.^3−6^ More recently, Zhou et al.^7^ reached a milestone in the development of ASSBs using a Li_2_In_*x*_Sc_0.66–*x*_Cl_4_ electrolyte. The ASSB with this electrolyte possessing a 2 × 10^–3^ S/cm ionic conductivity and a 4.7 × 10^–10^ S/cm electronic conductivity, reached 3000 cycles at 80% capacity retention when cycled between 2.8–4.3 V vs Li/Li^+^ using LiNi_0.85_Co_0.1_Mn_0.05_O_2_ as the cathode (6.21 mg/cm^2^) at 3C.^7^ Considering these promising results, further improvements and fundamental understanding of such electrolytes may accelerate the development of practical ASSBs.

A strategy that has been successfully employed to tune the ionic conductivity of Li_3_M(III)X_6_ materials is halogen substitution.^8−13^ Specifically for the compositional tie-line between Li_3_YCl_6_ and Li_3_YBr_6_, the Li_3_YBr_3_Cl_3_ composition was shown to have a 7.2 × 10^–3^ S/cm ionic conductivity at room temperature (compared to the ∼1 × 10^–3^ S/cm of the end members Li_3_YCl_6_/Li_3_YBr_6_ ref (2)). Based on thermodynamic phase equilibrium calculations, it has been predicted that Li_3_YCl_6_ should be stable between 0.62 and 4.21 V vs Li/Li^+^ and Li_3_YBr_6_ between 0.59 and 3.15 V vs Li/Li^+^ (ref (14)). According to these calculations, the materials would decompose into the YX_3_ precursor and the halogen gas X_2_ at high potentials (oxidation) and to metallic Y and LiX (X = Cl, Br) at low potentials (reduction). These calculations suggest that introducing Br may reduce the high-voltage stability, leading to a trade-off between cathode compatibility and ionic conductivity. However, it has been shown that such calculations do not always reflect the practically relevant stability window of solid electrolytes, as the (possible) formation of intermediate phases are not considered.^15^

More generally, it has been proposed that the reduction and oxidation potential of the solid electrolyte upon lithiation/delithiation is a more accurate measure of the practical electrochemical stability window because the energetics of the reaction intermediates and non-equilibrium states are captured.^15^ The stability window based on the oxidation and reduction potentials was found to be larger compared to thermodynamic calculations and matched the experimentally measured oxidation and reduction voltages for argyrodite-type Li_6_PS_5_Cl, garnet-type Li_7_La_3_Zr_2_O_12_, and NASICON-type Li_1.5_Al_0.5_Ge_1.5_(PO_4_)_3_ (ref (15)). For Li_3_YBr_6_, this method proposes a stability window between 0 and 3.43 V vs Li/Li^+^ (ref (16)) (compared to the previously predicted window of 0.59–3.15 V vs Li/Li^+^). For Li_3_YCl_6_, no data simulated data using this method isavailable.

Specifically for Li_3_M(III)Cl_6_ (M(III) = Bi, Dy, Er, Ho, In, Lu, Sc, Sm, Tb, Tl, Tm, Y), the influence of the M(III) in different chlorides on the high-voltage stability was calculated to be between 4.26 and 4.38 V, with the majority at 4.26 V vs Li/Li^+^ (ref (17)). Recent experimental results showed that the difference in the high-voltage stability between solid-electrolytes with different M(III)/M(IV) is larger than expected in some cases, and that not only the electrochemical stability is relevant in such systems, but also the stability against oxygen release especially in combination with high capacity LiNi_0.85_Co_0.1_Mn_0.05_O_2_ (ref (18)).

In this study, we report an experimental study of the substitution series Li_3_YBr_*x*_Cl_6–*x*_ (*x* = [0, 6] steps of 1.5) and investigate the influence of the halogen composition on the crystal structure, ionic conductivity, and the electrochemical stability window. It is found that the composition Li_3_YBr_*x*_Cl_6–*x*_ (*x* = 1.5) is a good compromise, with increased ionic conductivity compared to Li_3_YCl_6_ while maintaining the higher oxidative stability of Li_3_YCl_6_ compared to the bromide end-member.

## Methods

### Synthesis of Halide SSEs

For the ampule synthesis of Li_3_YBr_*x*_Cl_6–*x*_, stoichiometric mixtures of the precursors (*x* ≤ 3: LiCl (>99%, Sigma-Aldrich), LiBr (>99.99%, Sigma-Aldrich), YCl_3_(99.99%, Sigma-Aldrich); *x* > 3: LiCl, LiBr, YBr_3_(99.9% REO)) were filled in quartz ampules in an argon-filled glovebox. Then the ampules were evacuated and refilled to 200 mbar argon, flame-sealed, and placed in a muffle furnace. The powder mixtures were heated to 650 °C (above their melting point) in 4 h, held at that temperature for 24 h, and then cooled down to room temperature over 24 h. The resulting products were pulverized using pestle and mortar until a fine powder was obtained. The mechanochemical synthesis of Li_3_YBr_*x*_Cl_6–*x*_ with *x* = 3 was carried out by ball milling of precursors (YCl_3_ and LiBr in a molar ratio of 1:3). Each 45 mL ZrO_2_ ball mill jar (Fritsch) was filled with a total amount of 3 g of material with 20 g of Φ3 mm and 60 g of Φ10 mm ZrO_2_ balls as milling media and tightly closed in the glovebox. The milling was conducted for 288 cycles of 5 min (each followed by 5 min rest) with a rotation speed of 500 rpm. After every 2 h, the mixtures were homogenized in the argon-filled box by scraping off the material pressed to the wall. A small part of the resulting material was taken out every 4 h for analysis. The as-milled products were subsequently put in the furnace for a sequential crystallization. The annealing was performed in quartz ampules in the same way as described above, at a temperature of 200–600 °C for 5 h with a heating rate of 2 °C per minute. The ampules were naturally cooled down to room temperature after annealing.

### X-ray Diffraction

The samples were analyzed using a X’Pert-Pro diffractometer (PANalytical) equipped with a Cu Kα radiation (λ = 1.5406 Å) source operating at 45 kV and 40 mA. The samples were prepared by filling the powder into an airtight holder, which consisted of a silicon zero-diffraction wafer separated from the environment with a Kapton film arch. The data were collected at room temperature and atmospheric pressure by scanning over the 2θ range of 10 to 90°. The measured X-ray diffraction patterns were analyzed by Le Bail refinement using GSAS-II.^19,20^

### Neutron Diffraction

Neutron diffraction was performed on the PEARL^21^ diffractometer of the Reactor Institute Delft at a wavelength of 1.667 Å. The sample was filled in 7 mm diameter vanadium cans, which were filled in an argon-filled glovebox and sealed using rubber O-rings.

### Rietveld Refinement

Rietveld refinement of the neutron diffraction data was performed using GSAS-II.^36,37^ For the monoclinic phase, Y and Li were found to occupy the same crystallographic site. Due to the opposite sign coherent scattering length of these atoms (*b*_Li_ = −1.90 fm, *b*_Y_ = 7.75 fm), this led to a diminution of the signal intensity. As the full occupancy of the site is also not known, it was not possible to automatically refine this ratio due to divergence of the fit. Therefore, the refinement was done manually trying out (almost) all possible configurations, and deciding on the best fit based on visual inspection and the goodness of fit. Due to this difficulty, no conclusions have been drawn in this report on the differences between the occupancy parameters extracted. Crystal structures were visualized in VESTA.^22^

### AC Impedance

AC impedance spectroscopy was used to determine the ionic conductivity of the synthesized powders. A homemade cell was used consisting of an insulating alumina hollow cylinder with two stainless steel plungers that are used both to initially compress the powder into a pellet and act as current collectors. The cell was assembled in an Ar-filled glovebox by filling 100 mg of powder into the alumina cylinder (inner diameter of Φ10 mm) and cold-pressing the powder into a pellet (392 MPa) using the steel plungers. The assembly was screwed tight under pressure using three (electronically insulated) screws. The measurements were performed on an Autolab electrochemical workstation (AUT86298) in a frequency range between 1 MHz and 100 Hz. The obtained data were fit using the commercial software RelaxIS (rhd instruments). The datasets were fit using a L-R-CPE circuit, which was validated by the Kramers–Kronig relations (see supporting files). The values of the parameter fits were used to calculate the ionic conductivities and the Arrhenius relationship. The error of individual conductivity measurements was calculated from Gaussian error propagation as proposed by Krasnikova et al.:^23^
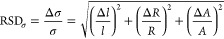
considering the influence of pellet thickness *l* and the error of the fit of the resistance *R*. Due to the nature of the setup, the error in the area, *A*, is considered negligible by the authors. A 5% error was estimated for the pellet thickness (from the standard deviation of the thickness measured by the micrometer at different positions across the pellet), due to deviations in the thickness across the pellet. The error of the activation energy was calculated based on the fit of the Arrhenius relationship.

### Electrochemical Measurements

The redox activity of the solid electrolytes was measured in the same assembly as the ionic conductivity, consisting of an alumina cylinder and stainless steel plungers.

The solid electrolyte–carbon composites were made by ball milling of the solid electrolytes with Super C45 and carbon nanofibers in the weight ratio of 0.85:0.10:0.05 for 2 h at 300 rpm. This mixture was tested for both oxidation and reduction stability. Depending on the reaction, either a lithium source or drain is needed as the counter electrode with a known constant potential.

Lithium titanate (Li_4_Ti_5_O_12_, from altair nano) was used as a lithium drain. As the material does not have a constant potential at the beginning of the lithiation, the material was prelithiated chemically using *n*-butyllithium (1.6 M from Merck Sigma). The amount of *n*-butyllithium needed to half lithiate the Li_4_Ti_5_O_12_ to Li_5.5_Ti_5_O_12_ (prelithiated LTO) was calculated, and the reaction was carried out in hexane while stirring continuously inside an Ar-filled glovebox. After the reaction, the hexane was evaporated. For the measurements, the prelithiated LTO, the Li_3_YBr_*x*_Cl_6–*x*_ solid electrolyte, Super C45, and carbon nanofibers were ball milled with the ratio of 0.45:0.40:0.10:0.05 for 2 h at 300 rpm. Twenty-five milligrams of that mixture was used as the electrode.

As a lithium source, Li–In alloy was used. A Φ7 mm indium foil (∼58 mg) was prelithated with Φ3 mm Li foil (∼1 mg) by pressing the foils on top of each other. Li in indium is at 0.62 V vs Li/Li^+^, and therefore, the in foil will lithiate spontaneously.

The fabrication of ASSBs starts with a cold pressing of 150 mg of the Li_3_YBr_*x*_Cl_6–*x*_ solid electrolytes under the pressure of 392 MPa. Sequentially, 16 mg of the halide–carbon mixture was placed and pressed under the pressure of 490 MPa on the one side, and the reference electrodes were then pressed on the other side (prelithiated LTO and In–Li alloy under the pressure of 490 and 50 MPa, respectively).

The oxidation and reduction voltages were measured by drawing a constant current of 6.5 μA/cm^2^ to 0 V vs Li/Li^+^ (reduction of the solid electrolyte), i.e., galvanostatically charging to 3.5 V versus prelithiated LTO or discharging to −0.62 V versus the In–Li alloy with a current density of 6.5 μA/cm^2^. For the determination of the electrochemical stability window, the differential capacity was calculated based on the charge and discharge profiles.

### Battery Assembly

ASSBs were assembled using single crystal NCM 811 (MSE supplies) as a cathode and Li–In as an anode. To ensure reversibility on the anode side, argyrodite Li_6_PS_5_Cl (acquired from NEI corporation) was used as an interlayer between the halide SE and the Li–In alloy. During assembly, the first 50 mg of the halide solid electrolyte was pressed at 20 MPa for 2 min. Then 50 mg of argyrodite was added on the other side and the pressing repeated. Around 10 mg of NCM 811 (composite 70% NCM 811 and 30% halide solid electrolyte, mixed using pestle and mortar) was added on the halide SE side. Prelithiated In foil (52 mg of indium foil, 8 mm diameter, and 2 mg of Li-metal foil pressed on) was then added on the argyrodite Li_6_PS_5_Cl side. The whole cell was pressed at 5 MPa for 1 min. The cells were cycled at C/10 between 2.75 and 4.3 V vs Li/Li^+^.

## Results and Discussion

**Figure 1:**
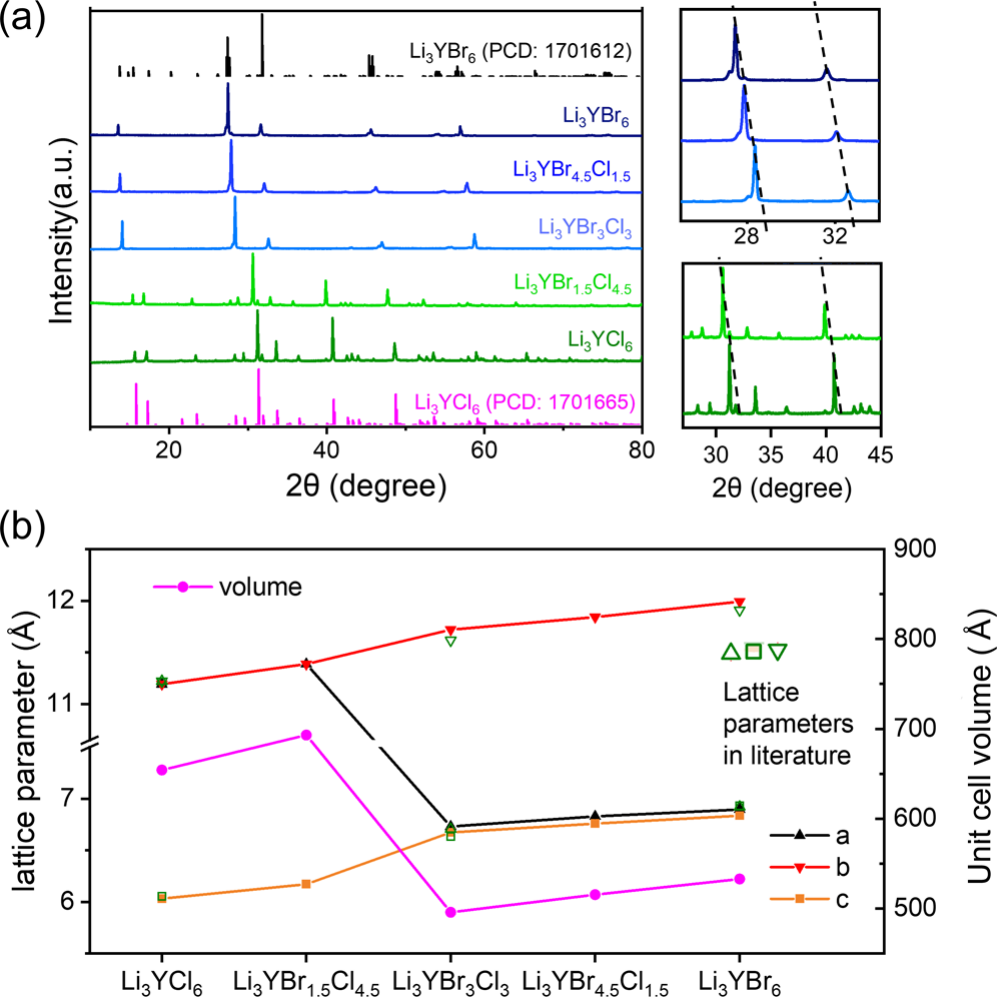
(a) X-ray diffraction patterns of the series of Li_3_YBr_*x*_Cl_6–*x*_ (x = 0–6) solid electrolytes. Magnified images of a group of characteristic peaks from the diffraction patterns of the Li_3_YBr_*x*_Cl_6–*x*_ samples are shown. (b) Evolution of the lattice parameters of Li_3_YBr_*x*_Cl_6–*x*_ as a function of *x* as obtained from the Le Bail refinement of the X-ray diffraction data. For comparison, the lattice parameters of each halide achieved from previous reports are shown (Li_3_YBr_6_/Cl_6_ from ref (2), Li_3_YBr_3_Cl_3_ from ref (10)).

A series of Li_3_YBr_*x*_Cl_6–*x*_ (*x* = [0, 6] steps of 1.5) solid electrolytes were synthesized by direct co-melting of the precursors in evacuated silica ampules at 650 °C. The powders obtained were phase-pure for all compositions. The X-ray diffraction patterns shown in Figure 1a clearly show that the materials crystallize in two different phases, depending on the ratio between Cl and Br.

**Figure 2:**
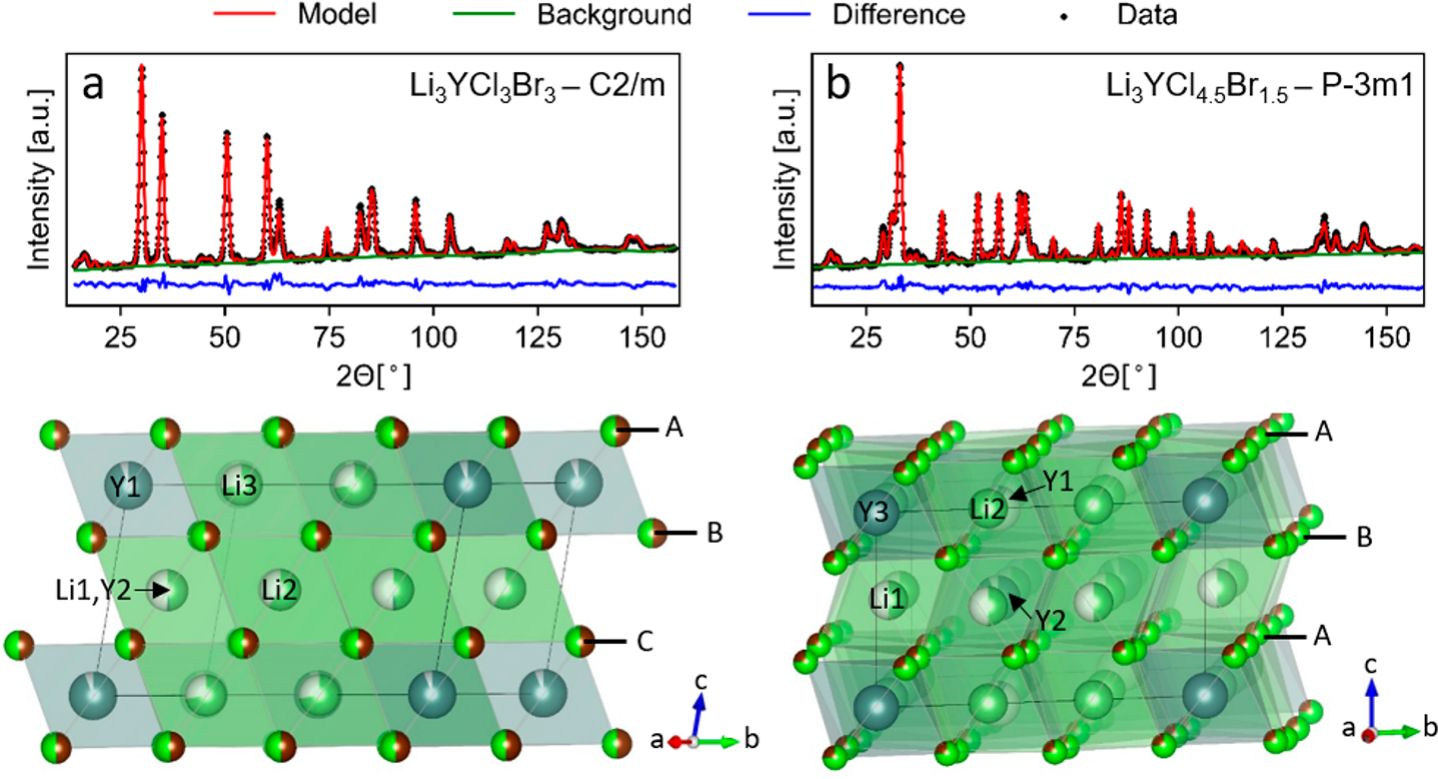
Rietveld refinement of the neutron diffraction data (top) and crystal structure (bottom) for the monoclinic (a) and trigonal (b) phase of Li_3_YBr_*x*_Cl_6–*x*_. (a) Structure solution of Li_3_YBr_*x*_Cl_6–*x*_ for *x* = 3 The cubic close packing-like framework is highlighted along the *c*-direction, as illustrated by the alternating layers A, B, and C. The Y atoms mostly occupy the Y1 (2a) site, with some on the Y2(4h) site. The 4h site is shared with Li. The lithium is distributed across the 4h site and the other empty octahedra. (b) Structure solution for Li_3_YBr_*x*_Cl_6–*x*_ for *x* = 1.5. The hexagonal close packing like framework is along the *c*-direction, as indicated by the alternating A and B layers. There is one fully occupied Y site, i.e., Y3(1a). The Y2(2d) site is almost fully occupied, while the Y1(2d) site is only 7% occupied.

The Li_3_YCl_6_ sample crystallized in the trigonal *P*3̅*m*1 (#164)^24^ space group, as does Li_3_YBr_*x*_Cl_6–*x*_ with *x* = 1.5. The materials with *x* > 1.5, as well as the full bromide Li_3_YBr_6_, crystallize in monoclinic *C*2/*m* (#12)^25^ space group. Due to the larger anion radius of Br, the lattice parameters increase as Br is added to the system (Figure 1b). Between the compositions Li_3_YBr_*x*_Cl_6–*x*_, *x* = 1.5 and *x* = 3, where the phase transition occurs, the volume of the unit cell decreases due to the smaller unit cell size of the monoclinic phase. The volume per chemical formula unit increases linearly with bromine content (SI Figure S1). With conventional Rietveld refinement, it was not possible to refine the X-ray data due to mismatch in peak intensities, especially the peaks between 2θ = 18–25° for the *C*2/*m* phase. To investigate this effect, different synthesis methods were applied (mechanochemical synthesis, mechanochemical synthesis + annealing step, co-melting) comparing the resulting morphology and diffraction patterns (SI Figures 2 and S3 and Text 1). While mechanochemical synthesis leads to a fine powder with no distinct features in the microstructure, heat treatment above 500 °C leads to the formation of platelets (SI Figure 4), which corresponds to the disappearance of the Bragg peaks between 2θ = 18–25° (SI Figure 2). This suggests that the anisotropic, platelet-like morphology, which can show as preferred orientation due to nonrandom orientation of the powder when the X-ray diffraction data is measured in Bragg–Brentano geometry, could be at least a source of the discrepancy. However, recent literature has reported the occurrence of stacking faults in Li_3_YCl_6_^26^ and Li_3_HoBr_*x*_I_6–*x*_,^8^ presenting an alternative origin for the discrepancy (SI Text 1). As described in ref (26), neutron diffraction data are less sensitive to stacking faults compared to X-ray diffraction in Li_3_YCl_6_, due to the different contrast. Further, due to the larger amount of sample and the transmission geometry, the neutron diffraction data are also less sensitive to preferred orientation, compared to X-ray diffraction data measured in Bragg–Brentano geometry. Therefore, neutron diffraction was performed to confirm the monoclinic average symmetry and to learn more about the structural effect of anion substitution in Li_3_YBr_*x*_Cl_6–*x*_ by means of Rietveld refinement (Figure 2a,b, SI Figures S5–S14 including visual models, and SI Tables S1–S5).

The crystal structures of the trigonal and monoclinic phases have distinct structural characteristics (Figure 2). The trigonal structure with the compositions Li_3_YBr_*x*_Cl_6–*x*_ at *x* = 0 and 1.5 is built by hexagonal close-packing (hcp)-like arrangement of the halogen atoms, with the Y and Li in octahedral positions. The Rietveld refinement shows that the Cl and Br atoms distribute statistically across the halogen sites (as consistent with literature; see refs (8−10)) and small differences in the Y and Li occupancies (SI Tables S4 and S5). The monoclinic phase with the compositions Li_3_YBr_*x*_Cl_6–*x*_ at *x* = 3, 4.5, and 6 on the other hand, shows cubic close packing (ccp) of the halogen atoms. Similar to the trigonal phase, the Cl and Br distribute statistically across the halogen sites and the Y and Li reside on octahedral sites. Also here, there appear to be small redistributions of the Li and Y, but there is no clear trend as a function of *x* (SI Tables S1–S3). No tetrahedral sites were necessary for a physically consistent refinement as reported in isostructural chlorides.^27,28^ Despite that the tetrahedral voids are not occupied on average, they are still available as interstitial sites for the diffusion pathways.

**Figure 3:**
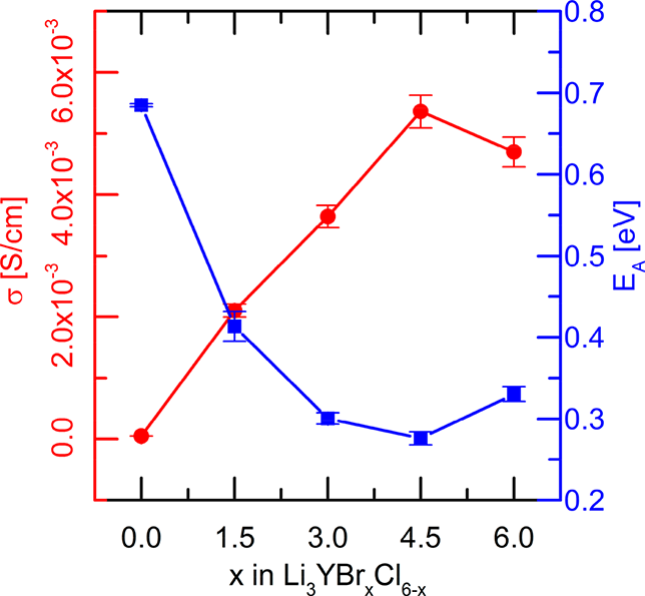
Ionic conductivity σ [S/cm] at 30 °C and activation energies extracted from the Arrhenius relationship (see SI Figures S15–S19) of Li_3_YBr_*x*_Cl_6–*x*_.

The ionic conductivities of the various Li_3_YBr_*x*_Cl_6–*x*_ solid electrolytes as a function of temperature are determined by broadband AC impedance spectroscopy (Figure 3; for fits of individual measurements and validation of the circuit model by the Kramers–Kronig relationship, see supplementary files). The ionic conductivity at 30 °C is lowest for Li_3_YCl_6_ at 0.05 × 10^–3^ S/cm. Already the small amount of Br in Li_3_YBr_1.5_Cl_4.5_ leads to a large increase in ionic conductivity to 2.1 × 10^–3^ S/cm, where the material is still in the trigonal phase. The ionic conductivity increases further as *x* is increased and reaches a maximum of 5.36 × 10^–3^ S/cm for Li_3_YBr_4.5_Cl_1.5_ in the monoclinic phase. The activation energy reflects a similar trend: it has the highest value for the chloride end-member Li_3_YCl_6_ (0.685 eV), reduces to 0.413 eV for Li_3_YBr_1.5_Cl_4.5_, and reaches its minimum of 0.276 eV for Li_3_YBr_4.5_Cl_1.5_ (Figure 3; see SI Figures S15–S19 for the complete conductivity datasets and Arrhenius relationship fits). There are many reasons that could cause differences in ionic conductivities in this substitution series:

- The association energy of Li with Br is smaller than that with Cl, due to the smaller electronegativity/larger lattice polarizability.^29−31^
- As the crystal structure transitions from the trigonal to the monoclinic phase, the anionic framework transforms from hexagonal close packing (hcp) to cubic close packing (ccp). In the hexagonal close packing, lithium can jump through face-sharing octahedral–octahedral (oct–oct), octahedral–tetrahedral–octahedral (oct–tet–oct), and tetrahedral–tetrahedral (tet–tet) pathways. In the cubic close packed framework, only oct–tet–oct paths exist.^32^ Calculations have shown that hcp chlorides have slightly lower activation energies (0.25 eV oct–oct and 0.29 oct–tet–oct) compared to ccp bromides (0.28 oct–tet–oct).^14^
- With the introduction of more (larger) Br, the lattice expands, rendering larger polyhedral faces (“bottlenecks”) for the Li to jump through.^33,34^
- (Small) changes in the Y and/or Li site occupancies may affect the ionic conductivity.^35,36^
- Finally, there are reports that the increase in configurational entropy in mixed (poly)anion substituted samples may alter the ionic conductivity.^37,38^

While it is not possible to deconvolute these factors based on the data presented here, some general observations can be made. First of all, if the increase in ionic conductivity due to the larger lattice polarizability was the only/main factor at play, the activation energy should decrease monotonically with *x*, which is not the case. A similar argument can be made for the configurational entropy, if it was the only/main factor at play the maximum in ionic conductivity should be found at *x* = 3 in Li_3_YBr_*x*_Cl_6–*x*_. The trigonal phase has much higher activation energies (0.685 eV for *x* = 0, 0.413 eV for *x* = 1.5) compared to the monoclinic phase (0.300 eV for *x* = 3, 0.276 eV for *x* = 4.5, 0.330 eV for *x* = 6), it is therefore rather likely that the ccp-like packing is more favorable, as also postulated theoretically in literature for sulfides (where, however, Li preferentially resides in tetrahedral coordination).^32^ Considering the large change in ionic conductivity from Li_3_YCl_6_ to Li_3_YBr_1.5_Cl_4.5_, it is likely that the increase of the lattice parameters aids the conductivity in the *P*3̅*m*1 phase. To investigate the effect of site occupancies, more compositions should be considered and X-ray diffraction data should be refined simultaneously with the neutron diffraction data for more accurate site occupancies. The deconvolution of these different effects and determination of detailed site occupancies are subject of future research.

**Figure 4:**
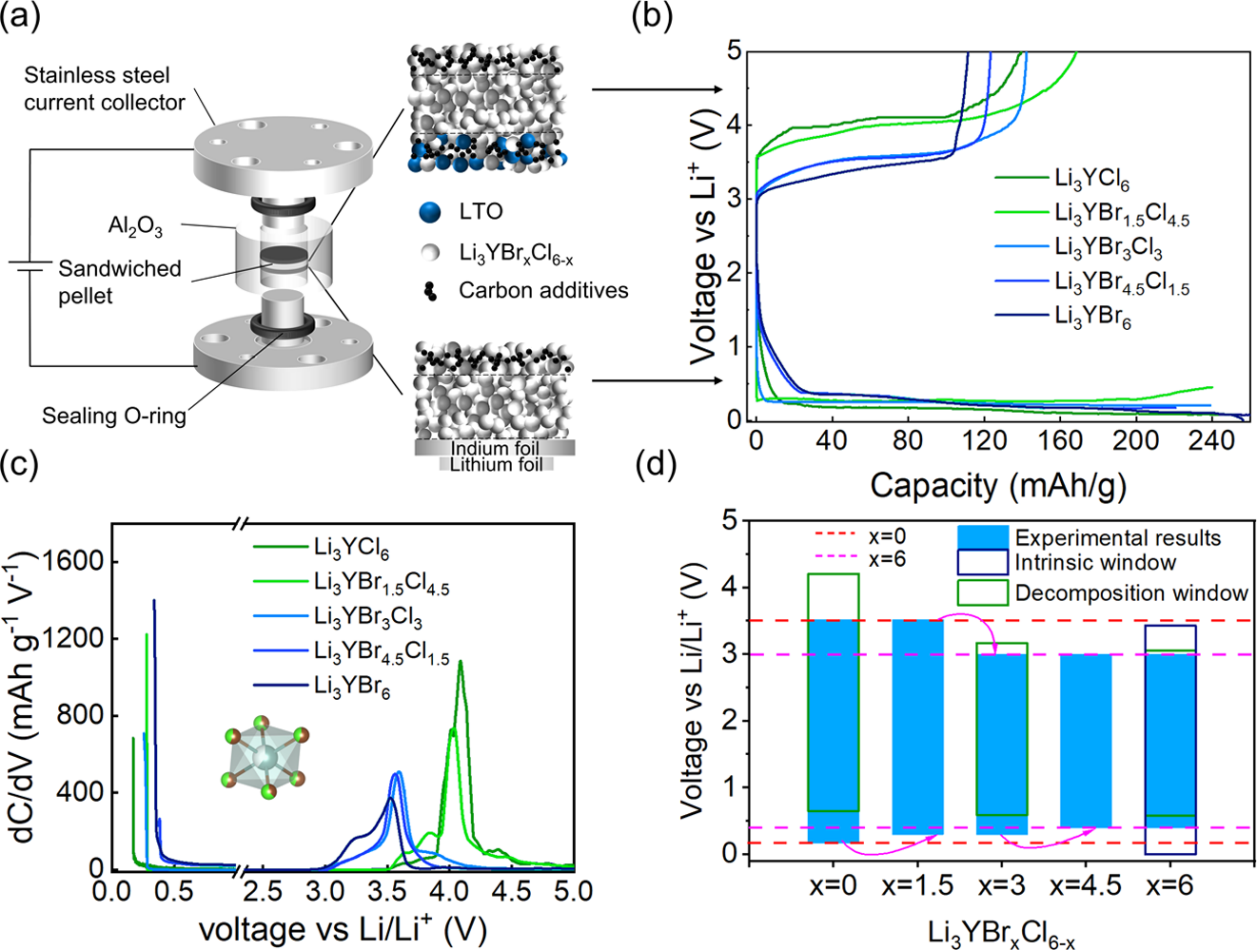
Electrochemical stability assessment in ASSBs with Li_3_YBr_*x*_Cl_6–*x*_ (a) Schematic view of the cell configuration. Different configurations of pellets are pressed between the blocking electrodes. Carbon and Li_3_YBr_*x*_Cl_6–*x*_ composites are used as working electrodes, while Li_4_Ti_5_O_12_ (LTO)-C and In–Li are used as counter electrodes, respectively. (b) Galvanostatic charge and discharge curves of Li_3_YBr_*x*_Cl_6–*x*_-C|Li_3_YBr_*x*_Cl_6–*x*_|LTO-C and Li_3_YBr_*x*_Cl_6–*x*_-C|Li_3_YBr_*x*_Cl_6–*x*_|In–Li ASSBs, respectively. (c) Differential capacity d*C*/d*V* curves of the redox activities of Li_3_YBr_*x*_Cl_6–*x*_ solid electrolytes. (d) Electrochemical stability window inferred from the experimental voltage profiles. The dashed lines indicate the redox potential barriers of Li_3_YCl_6_ and Li_3_YBr_6_, and the arrows indicate where the barriers shift within the Li_3_YBr_*x*_Cl_6–*x*_ solid electrolytes. The thermodynamic decomposition windows^14^ and intrinsic windows predicted based on DFT calculation^16^ are also plotted for comparison. The thermodynamic decomposition window is the voltage at which the material decomposes to the most stable known products, whereas the intrinsic window represents the voltage at which the solid electrolytes de/lithiate, allowing the formation of reaction intermediates.

Experimentally measuring the solid electrolyte stability window is challenging. Previous reports have often determined the electrochemical stability window using cyclic voltammetry (CV), which shows a pair of peaks to reflect the reduction and oxidation reactions.^10,39,40^ However, the short exposure time of a CV scan makes it challenging to probe the electrochemical stability window of solid electrolytes, in which the kinetics of decomposition are sluggish due to the limited contact area, poor electronic conductivity, and charge transfer.^15^ To circumvent these problems, the electrochemical stability was measured as reported in Schwietert et al.^15^ Electrochemical cells were assembled using a simultaneous reference and counter electrode (in this case a Li intercalation (Li_4_Ti_5_O_12_ (LTO-C)) and alloying compound (Li–In) with constant potential for the Li concentrations measured) and a composite of the solid electrolyte mixed with carbon black as a working electrode (see schematic Figure 4a). The composite was then oxidized and reduced using galvanostatic measurements at 6.5 μA/cm^2^, so that the oxidation and reduction potential could be measured with minimal overpotential. The voltage curve of these cells is shown in Figure 4b. The curves show clear plateaus with large specific capacities (100–240 mAh/g of solid electrolyte), indicating that there is significant oxidation and reduction of the solid electrolytes.

The onset of the voltage plateaus corresponds to the onset voltages of the peaks in the differential capacity curves (Figure 4c). This onset voltage is where decomposition is initiated, and is the limiting value used for the decomposition window that is plotted in Figure 4d. The plot shows that the oxidative stability of Li_3_YBr_*x*_Cl_6–*x*_ can be split into two groups, one with an oxidation onset at 3.0 V for 3 < *x* ≤ 6 and 3.5 V for *x* ≤ 3. This two groups can be distinguished in two ways, namely their crystal structure (*P*3̅*m*1 vs *C*2/*m*), as well as the dominant anion (Cl-rich vs Br-rich). Oxidation of the halogen would, according to thermodynamic calculations, lead to the formation of halogen gas. Therefore, two Br atoms would need to be in close proximity to each other. Low enough Br concentrations could ensure that the Br are enough separate in space that the necessity for to Br atoms in close proximity is not given and oxidation cannot happen. On the other hand, it could also be due to the trigonal crystal structure (or the hcp-like arrangement of the anions) which could be less prone to oxidize compared to the monoclinic structure (or ccp-like arrangement of the anions).

For Li_3_YBr_6_, the oxidation onset value from the dC/dV curve is at 3 V which is smaller than the calculated thermodynamic decomposition window (3.15 V) and the intrinsic (oxidation and reduction) window (3.43 V). For Li_3_YCl_6_, the oxidation onset at 3.5 V is also smaller compared to the calculated thermodynamic decomposition window (4.21 V).

It is interesting to consider the position of the maxima of the dC/dV curves, as in principle it could be more representative of bulk values, should interface effects lower the onset potential. For Li_3_YBr_6_, the maximum of the d*C*/d*V* curve is at 3.4 V, which corresponds with the voltage predicted by the calculations of the intrinsic window. For Li_3_YCl_6_, the maximum of the d*C*/d*V* curve is at 4.2 V, the value predicted for the thermodynamic decomposition window.

To conclude, the experimental data confirm the trend in oxidation potential predicted by theoretical calculations between Li_3_YBr_6_ and Li_3_YCl_6_, but neither calculation method accurately predicts the values observed here.

Considering the mixed halide samples Li_3_YBr_*x*_Cl_6–*x*_ (*x* = 1.5, 3, 4.5), there is a clear trend. Low Br concentrations do not affect the oxidation potential, as trigonal Li_3_YBr_1.5_Cl_4.5_ follows the behavior of the trigonal chloride end-member Li_3_YCl_6_. All other compositions, crystallizing in the monoclinic phase, Li_3_YBr_*x*_Cl_6–*x*_ (*x* = 3, 4.5) show similar behavior to monoclinic bromide end-member Li_3_YBr_6_ (see Figure 4d).

The electrolytes Li_3_YBr_*x*_Cl_6–*x*_ (*x* = 1.5, 4.5) were tested in full cells using single crystal NCM811/halide SE composite as cathode, halide SE as electrolyte, a Li_6_PS_5_Cl interlayer, and Li–In as anode (see Figure S20). Li_3_YBr_*x*_Cl_6–*x*_ with *x* = 1.5 showed a first cycle Coulombic efficiency of 79.95%, compared to 73.05% for *x* = 4.5 (in comparison, the same cathode tested in a liquid cell had 83.79% Coulombic efficiency). This is an indicator for less SE decomposition during the first cycle.

Concluding, results of the ionic conductivity and electrochemical window measurements show that there is a trade-off in the Li_3_YBr_*x*_Cl_6–*x*_ substitution range. While the ionic conductivity is highest at *x* = 4.5 in the monoclinic phase, the electrolyte starts to oxidize at 3 V, similar to Li_3_YBr_6_. Small Br concentrations, here represented by *x* = 1.5, increase the ionic conductivity significantly in the *P*3̅*m*1 phase but maintain the high oxidation potential of Li_3_YCl_6_.

## Conclusions

The substitution series Li_3_YBr_*x*_Cl_6–*x*_ (*x* = 0, 1.5, 3, 4.5, 6) were synthesized by co-melting of the precursors. The proposed crystal structures (trigonal *P*3̅*m*1/monoclinic *C*2/*m*) for Li_3_YCl_6_/Li_3_YBr_6_ are a good fit to neutron diffraction data as demonstrated by Rietveld refinement. The composition Li_3_YCl_4.5_Br_1.5_ crystallized in trigonal *P*3̅*m*1 (like Li_3_YCl_6_), and the compositions Li_3_YBr_*x*_Cl_6–*x*_ with *x* = 3, 4.5 in monoclinic *C*2/*m* (like Li_3_YBr_6_),

Already a small Br content (*x* = 1.5) increases the ionic conductivity by 2 orders of magnitude compared to Li_3_YCl_6_ (2.02 compared to 0.047 × 10^–3^ S/cm at 30 °C). The maximum ionic conductivity of 5.18 × 10^–3^ S/cm at 30 °C is reached for the monoclinic Li_3_Ybr_*x*_Cl_6–*x*_ with *x* = 4.5. The investigation of the oxidative stability confirm that the solid electrolytes with high Cl content have a higher oxidation potential compared to the ones with high Br content. A small amount of Br substitution (*x* = 1.5) does not affect the oxidation potential and the measurement shows similar behavior to stable Li_3_YCl_6_, whereas larger Br contents (*x* = 3, 4.5) show behavior similar to the less electrochemically stable Li_3_YBr_6_. These results show a clear trade-off between ionic conductivity and electrochemical stability in this substitution series.

We highlight the lightly Br-substituted Li_3_YCl_4.5_Br_1.5_ as the best compromise, achieving the “best of both end-members”, with a conductivity ∼200 higher than trigonal Li_3_YCl_6_ and an oxidative stability ∼0.5 V higher than monoclinic Li_3_YBr_6_. This work highlights that careful optimization of composition and substitutions in the anion framework provide prolific and rational avenues for designing the properties of future solid electrolytes.

## Supporting Information Available

Dependence of the synthesis method on X-ray diffraction pattern and microstructure; relationship volume per formula unit vs Br/Cl ratio; Rietveld refinement of neutron diffraction data, corresponding structure solution and visualized crystal structure for Li_3_YBr_*x*_Cl_6–*x*_ (*x* = 0, 1.5, 3, 4.5, 6); Arrhenius relationship obtained from AC impedance measurements for Li_3_YBr_*x*_Cl_6–*x*_ (*x* = 0, 1.5, 3, 4.5, 6); battery performance using electrolytes Li_3_YBr_*x*_Cl_6–*x*_ (*x* = 1.5, 4.5) (PDF)
Reports to the equivalent circuit fits of the impedance spectra of Li_3_YCl_3_Br_3_ (PDF)
Reports to the equivalent circuit fits of the impedance spectra of Li_3_YBr_6_ (PDF)
Reports to the equivalent circuit fits of the impedance spectra of Li_3_YCl_6_ (PDF)
Reports to the equivalent circuit fits of the impedance spectra of Li_3_YCl_15_Br_45_ (PDF)
Reports to the equivalent circuit fits of the impedance spectra of Li_3_YCl_45_Br_15_ (PDF)
Kramers–Kronig analysis of Li_3_YBr_6_ (PDF)
Kramers–Kronig analysis of Li_3_YCl_3_Br_3_ (PDF)
Kramers–Kronig analysis of Li_3_YCl_6_ (PDF)
Kramers–Kronig analysis of Li_3_YCl_15_Br_45_ (PDF)
Kramers–Kronig analysis of Li_3_YCl_45_Br_15_ (PDF)
