# Supplementary material for: Investigation of Structure, Ionic Conductivity, and Electrochemical Stability of Halogen Substitution in Solid-State Ion Conductor Li3YBrxCl6–x
Source: J Phys Chem C Nanomater Interfaces. 2022 Dec 16;127(1):125–32. doi: 10.1021/acs.jpcc.2c07910 (PMC9841563; doi:10.1021/acs.jpcc.2c07910)
Supplement: Supplementary file 1 — jp2c07910_si_001.pdf [file jp2c07910_si_001.pdf]

Supporting information

Investigation of Structure, Ionic Conductivity and Electrochemical Stability of Halogen-Substitution in Solid-State Ion Conductor $Li_3YBr_xCl_{6-x}$

Eveline van der Maas[ᵼ], Wenxuan Zhao[ᵼ], Zhu Cheng, Theodosios Famprikis, Michel Thijs, Steven R. Parnell, Swapna Ganapathy*, Marnix Wagemaker*

Department of Radiation Science and Technology, Faculty of Applied Sciences, Delft University of Technology, 2629JB Delft, The Netherlands

*E-mail: s.ganapathy@tudelft.nl, m.wagemaker@tudelft.nl,

[ᵼ]These authors contributed equally

SI Text 1

The difference between the diffraction patterns of the ball milled plus annealed samples and the co-melted samples (Figure S3) can arise from multiple factors: 1) the formation of a phase with higher symmetry; 2) preferred orientation of powders in the sample holder, 3) anisotropic crystallite size; 4) stacking faults. Considering the good fits of the refinement based on the neutron diffraction data (SI Figure 5, 7, 9, 11, 13), the formation of a higher symmetry phase is unlikely. To understand how the microstructures of these materials are influenced by the synthetic procedure, scanning electron microscope (SEM) images were taken to probe the morphology of the samples above (Figure S4). It can be observed that the $Li_3YBr_3Cl_3$ ball milled for 8h (BM-LYBC) shows a microstructure in which small spheres with a dimension of around 1 μm agglomerate. During the crystallization occurring during the annealing step at 400

℃, the microspheres are sintered, thereby the particle size increases to the order of 10 μm. On the other hand, increasingly laminar microstructures are shown in SEM images of $Li_3YBr_3Cl_3$ samples prepared by ball milling followed by annealing at 500 ℃ (AN500-LYBC) and only high temperature melting at 650 ℃ (HT-LYBC). The thin-flake morphology observed here may suggest an anisotropic growth of the crystal, which could be an indicator for preferred orientation. However, considering recent contributions in literature, it is likely that stacking faults are the reason[2,3] for the observed mismatch in peak intensities at low angles in the X-ray diffraction data. All these aspects show distinctively in the diffraction pattern, preferential orientation will change relative intensities depending on the degree of the averaging across the different orientations. Anisotropic size will preferentially broaden certain peaks, and stacking faults will lead to a more triangular peak shape. From the diffraction patterns in Figure S3, it is difficult to draw conclusions.

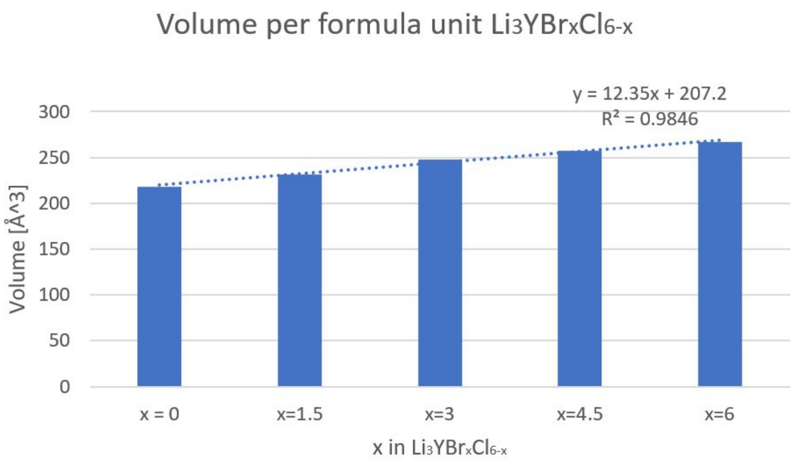

Figure S1:. Unit cell volume per formula unit as a function of composition

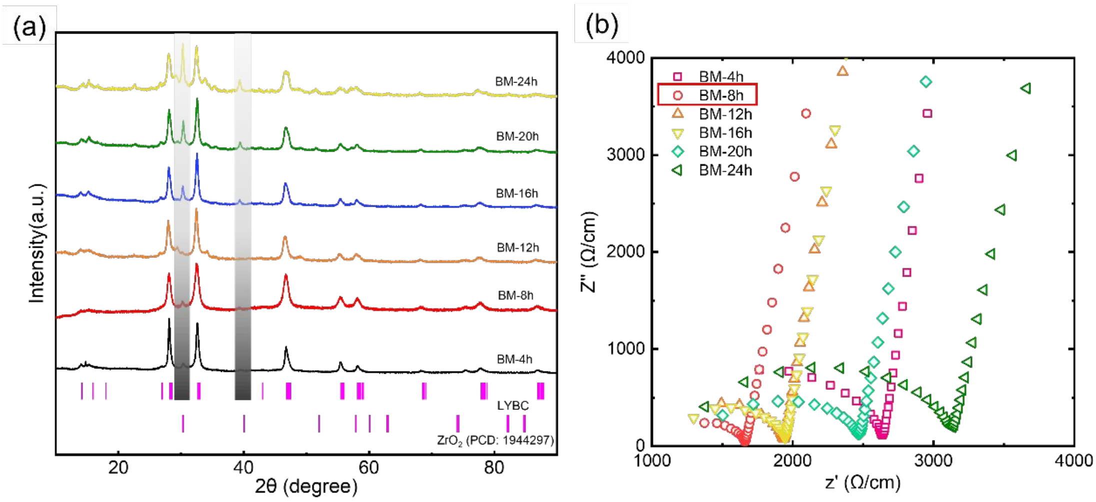

Figure S2: (a) XRD patterns of ball milled (BM) of $Li_3YBr_3Cl_3$ samples as a function of ball milling duration (4-24h). The highlighted peaks (grey) reflect the formation of the ZrO_2 phase that grows with increasing ball milling time, due to degradation/shedding from the ball milling balls. The materials prepared show broadened peaks, as is characteristic for ball milled-samples, and some peaks at low angles (2θ<20°) (b) Evolution of room temperature ionic conductivity of $Li_3YBr_3Cl_3$ (BM-LYBC) over the ball milling time is shown. The highest ionic conductivity was achieved after 8h of ball milling.

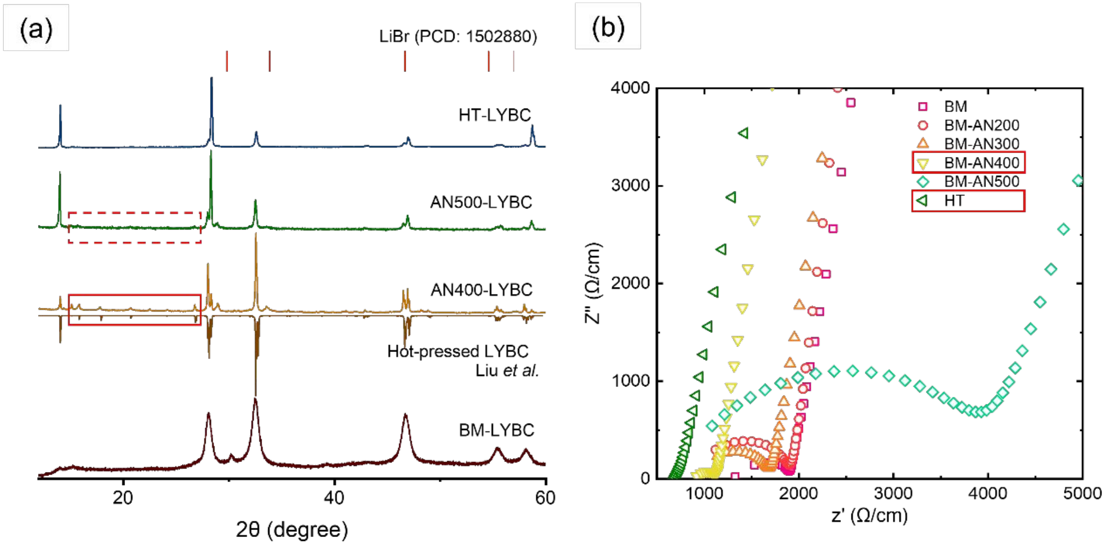

Figure S3: (a) XRD patterns of $Li_3YBr_3Cl_3$ (LYBC) samples synthesized by different methods (HT – melted at 650, AN-T annealed at temperature T in °C following the ball milling step, BM – mechanochemical synthesis by ball milling for 8h). The diffraction pattern of the LYBC with high crystallinity reported by Liu et al[1]. is also shown for comparison. Ball milling and annealing at 400-500°C both show some bragg peaks at 2θ<20°, which disappear for the HT sample (b) Comparison of Nyquist plots of the LYBC samples at room temperature. The highest ionic conductivity was achieved by comelting the precursors at 650°C (HT).

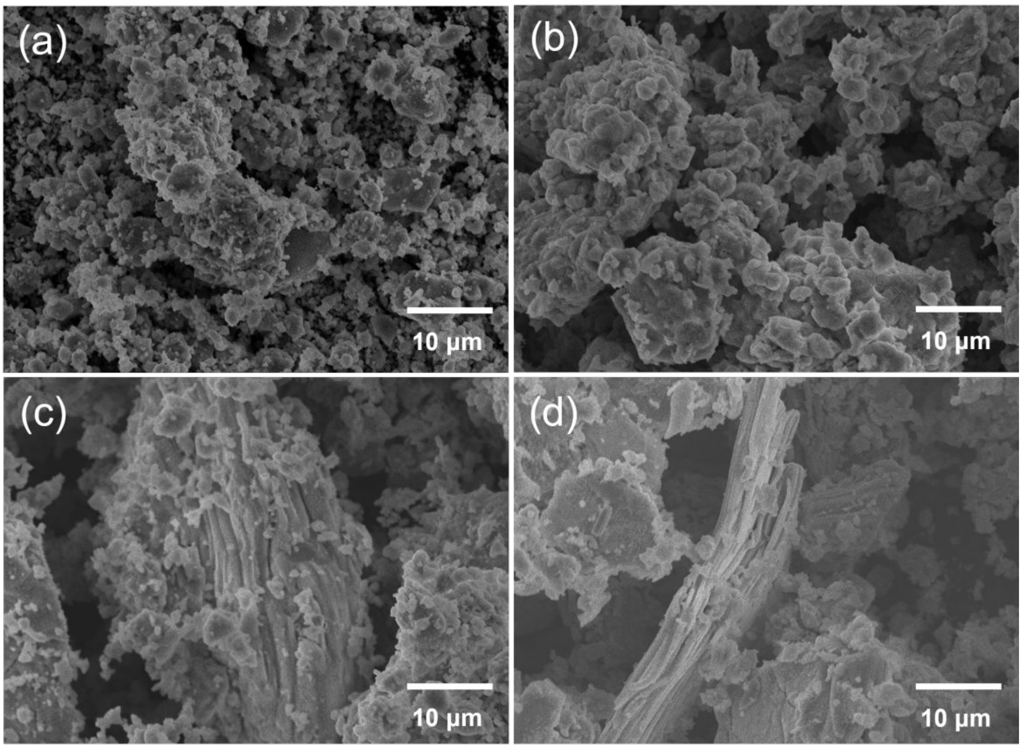

Figure S4: Scanning electron microscope (SEM) images of (a) BM-LYBC (b) AN400-LYBC (c) AN500-LYBC and (d) HT-LYBC. For abbreviations see SI Text 1.

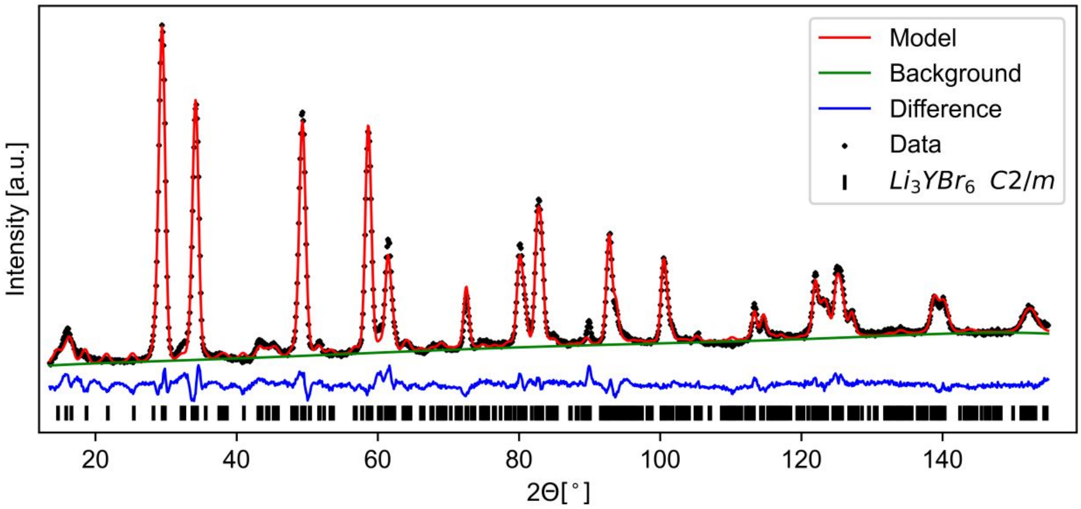

Figure S5: Neutron diffraction pattern with Rietveld refinement of Li_3YBr_6.

**a =**	6.9084	**b =**	11.9626	**c =**	6.8374	
**beta =**	109.527	**V =**	532.557	Space group	C2/m (12)	
**Site**	**x**	**y**	**z**	**Fractional Occupancy**	**Uiso**	**Wykhoff site**
Br1	0.2379	0.1664	0.2423	1	0.026	8j
Br2	0.7466	0	0.2471	1	0.026	4i
Li1	0	0.1840	0.5	0.65	0.093	4h
Li3	0.5	0	0.5	0.40	0.093	2d
Li2	0	0.3512	0	0.65	0.093	4g
Y2	0	0.1840	0.5	0.09	0.032	4h
Y1	0	0	0	0.82	0.032	2a

Table S1: Crystal structure obtained from the Rietveld refinement of the neutron diffraction data of Li_3YBr_6

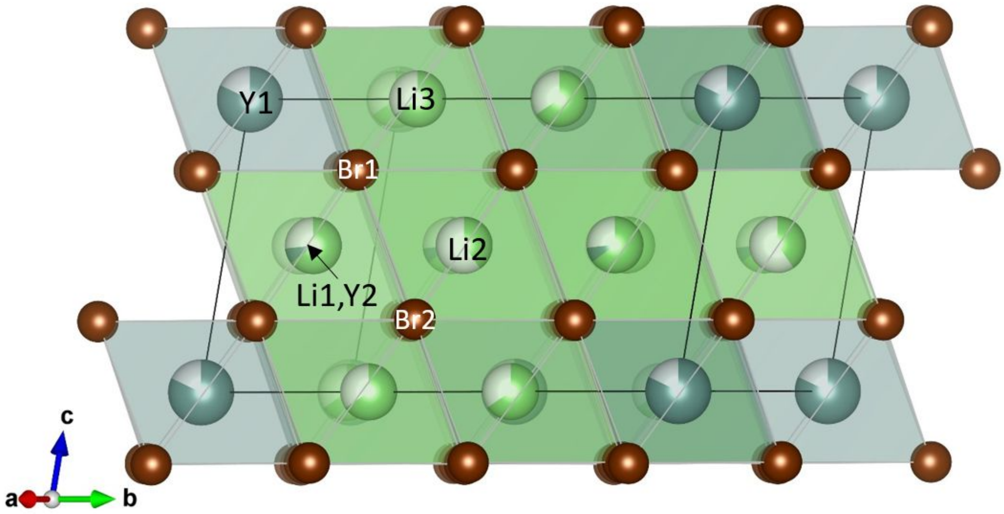

Figure S6: Crystal structure of Li_3YBr_6 visualized.

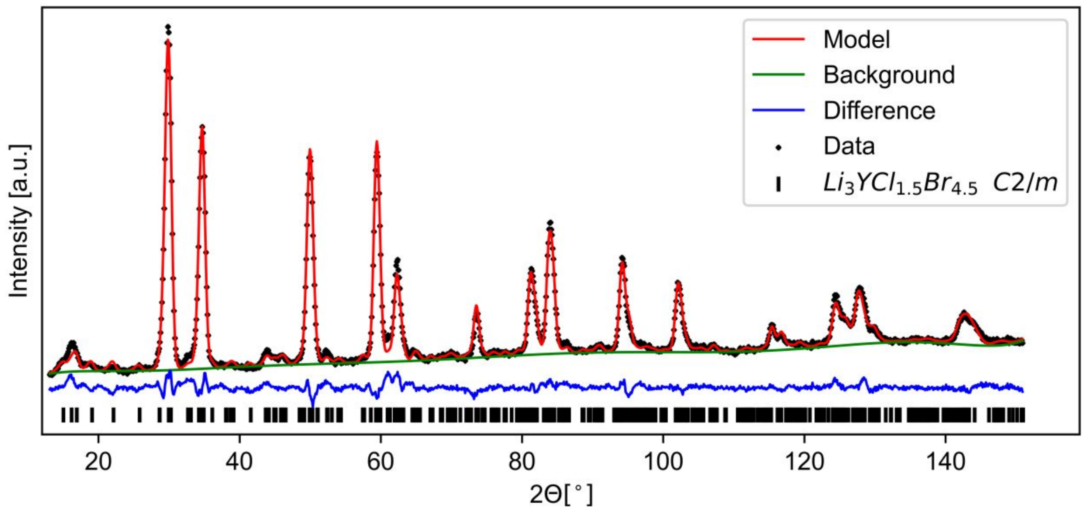

Figure S7: Neutron diffraction pattern with Rietveld refinement of $Li_3YCl_{1.5}Br_{4.5}$.

**a =**	6.8455	**b =**	11.8600	**c =**	6.7752	
**beta =**	109.442	**V =**	518.695	Space group	C2/m (12)	

Site	x	y	z	Fractional Occupancy	Uiso	Wykhoff site
Br1	0.2464	0.1662	0.2438	0.75	0.031	8j
Br2	0.7492	0	0.2409	0.75	0.031	4i
Cl1	0.2464	0.1662	0.2438	0.25	0.031	8j
Cl2	0.7492	0.0000	0.2409	0.25	0.031	4i
Li1	0	0.1840	0.5	0.60	0.035	4h
Li3	0.5	0	0.5	0.48	0.035	2d
Li2	0	0.3512	0	0.67	0.035	4g
Y2	0	0.1840	0.5	0.12	0.012	4h
Y1	0	0	0	0.77	0.012	2a

Table S2: Crystal structure obtained from the Rietveld refinement of the neutron diffraction data of $Li_3YCl_{1.5}Br_{4.5}$.

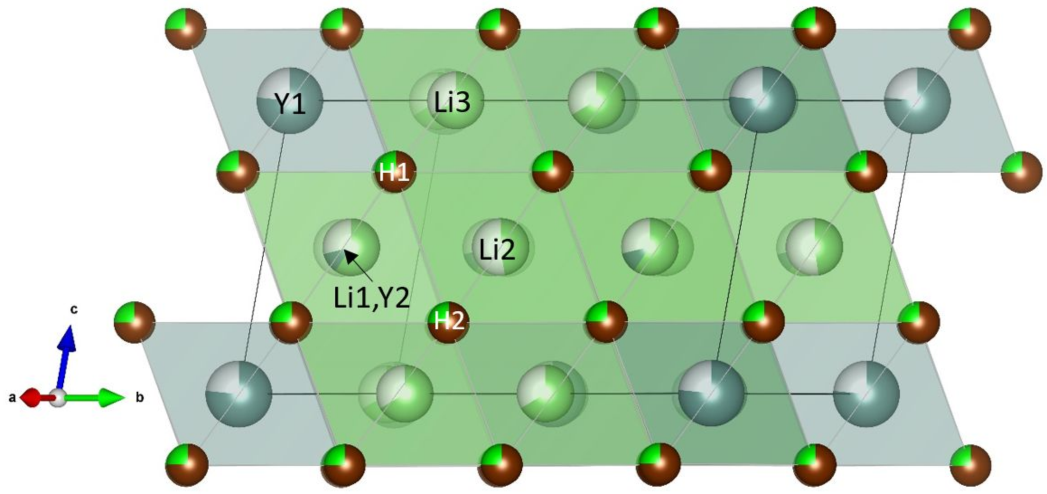

Figure S8: Crystal structure of $Li_3YCl_{1.5}Br_{4.5}$visualized. H=Cl, Br.

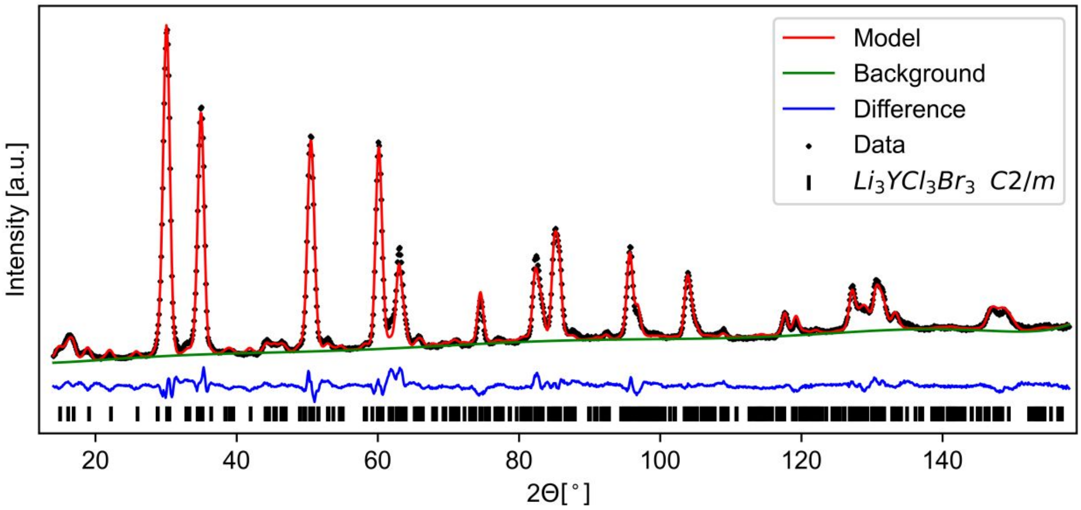

Figure S9: Neutron diffraction pattern with Rietveld refinement of $Li_3YCl_3Br_3$.

**a =**	6.7436	**b =**	11.6891	6.6687		
**beta =**	109.508	**V =**	495.490	Space group	C2/m (12)	
**Site**	**x**	**y**	**z**	**Fractional Occupancy**	**Uiso**	**Wykhoff site**
Br1	0.2426	0.1636	0.2420	0.50	0.031	8j
Br2	0.7495	0	0.2499	0.50	0.031	4i
Cl1	0.2426	0.1636	0.2420	0.50	0.031	8j
Cl2	0.7495	0	0.2499	0.50	0.031	4i
Li1	0	0.1670	0.5	0.47	0.109	4h
Li3	0.5	0	0.5	0.61	0.109	2d
Li2	0	0.3330	0	0.72	0.109	4g
Y2	0	0.1670	0.5	0.03	0.031	4h
Y1	0	0	0	0.94	0.031	2a

Table S3: Crystal structure obtained from the Rietveld refinement of the neutron diffraction data of $Li_3YCl_3Br_3$.

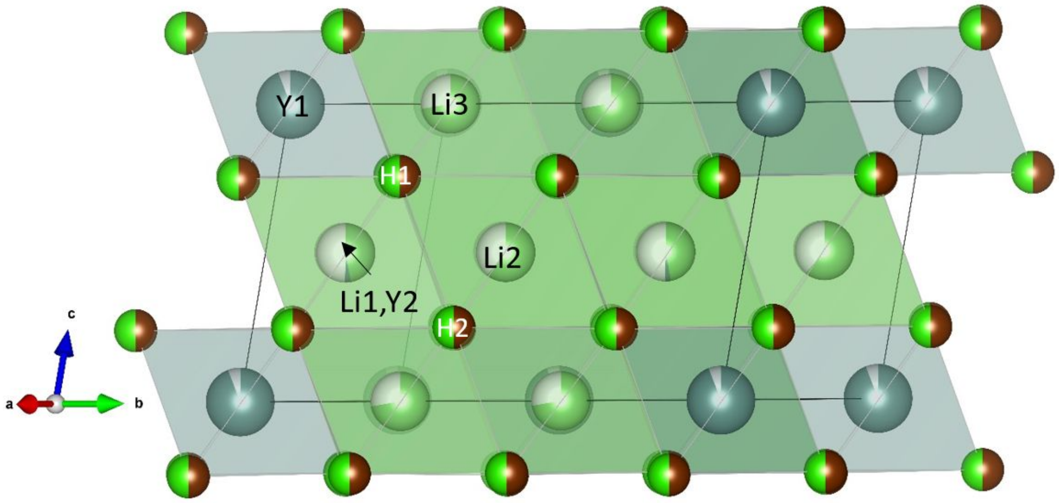

Figure S10: Crystal structure of $Li_3YCl_3Br_3$ visualized. H=Cl, Br.

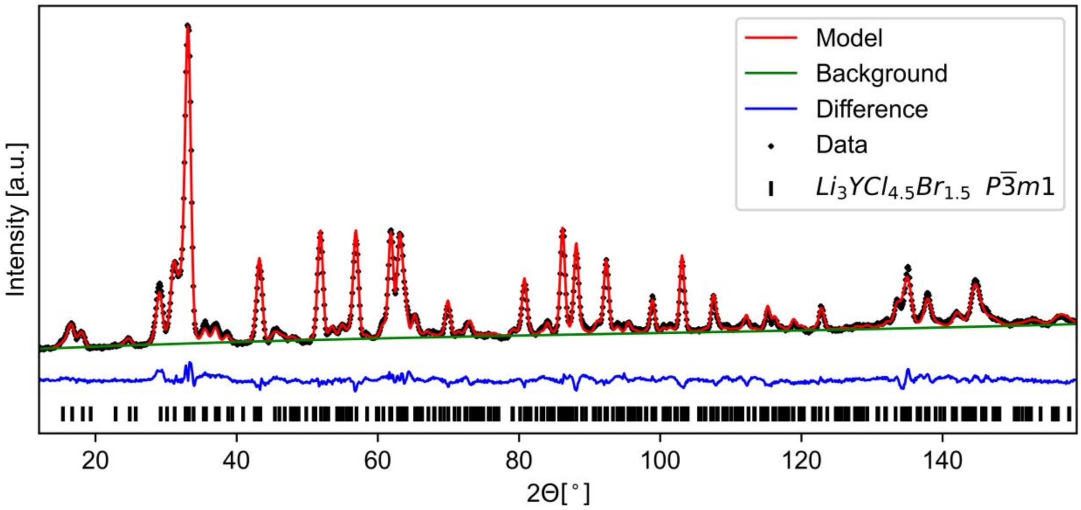

Figure S11: Neutron diffraction pattern with Rietveld refinement of $Li_3YCl_{4.5}Br_{1.5}$.

**a =**	11.3923	**b =**	11.3929	**c =**	6.1708	
**V =**	693.575			Space group	P-3m1 (164)	

Site	x	y	z	Fractional Occupancy	Uiso	Wykhoff site
Cl1	0.2215	0.7785	0.2676	0.75	0.025	6i
Cl2	0.5546	0.4454	0.2441	0.75	0.025	6i
Cl3	0.8865	0.1135	0.2319	0.75	0.025	6i
Br1	0.2215	0.7785	0.2676	0.25	0.165	6i
Br2	0.5546	0.4454	0.2441	0.25	0.165	6i
Br3	0.8865	0.1135	0.2319	0.25	0.040	6i
Li1	0.3397	0	0.5	0.5	0.040	6h
Li2	0.3397	0	0	1	0.040	6g
Y1	0.3333	0.6667	0.0506	0.07	0.025	2d
Y2	0.3333	0.6667	0.5100	0.93	0.025	2d
Y3	0	0	0	1	0.025	1a

Table S4: Crystal structure obtained from the Rietveld refinement of the neutron diffraction data of $Li_3YCl_{4.5}Br_{1.5}$.

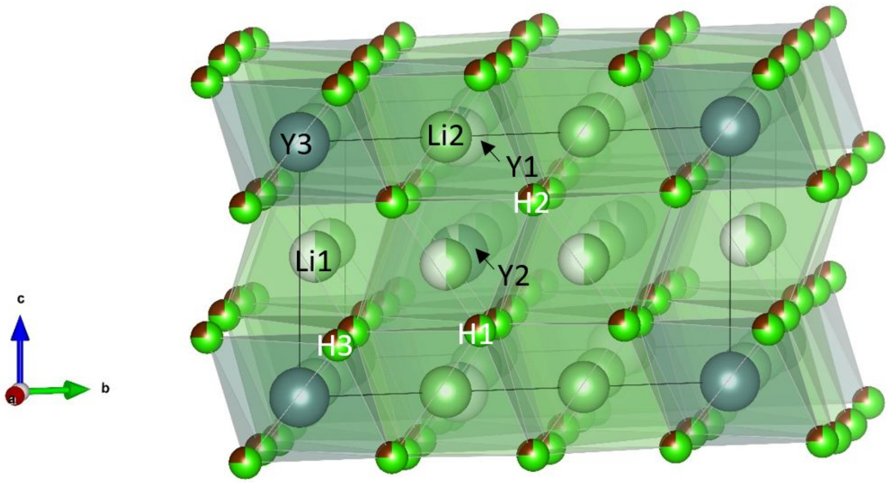

Figure S12: Crystal structure of $Li_3YCl_3Br_3$ visualized. H=Cl, Br.

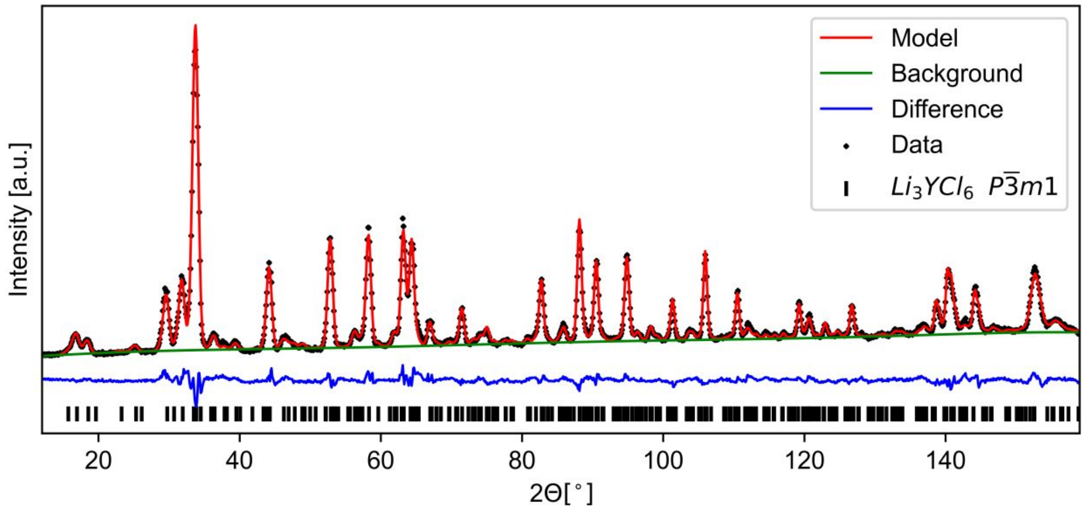

Figure S13: Neutron diffraction pattern with Rietveld refinement of Li_3YCl_6.

**a =**	11.1821	**b =**	11.1821	**c =**	6.0278
**V =**	653.911			Space group	P-3m1 (164)

Site	x	y	z	Fractional Occupancy	Uiso	Wykhoff site
Cl1	0.2215	0.7785	0.2676	1	0.022	6i
Cl2	0.5546	0.4454	0.2441	1	0.022	6i
Cl3	0.8865	0.1135	0.2319	1	0.022	6i
Li1	0.3397	0	0.5	0.66	0.075	6h
Li2	0.3397	0	0	0.84	0.075	6g
Y1	0.3333	0.6667	0.0506	0.07	0.020	2d
Y2	0.3333	0.6667	0.5100	1.00	0.020	2d
Y3	0	0	0	0.86	0.020	1a

Table S5: Crystal structure obtained from the Rietveld refinement of the neutron diffraction data of Li_3YCl_6.

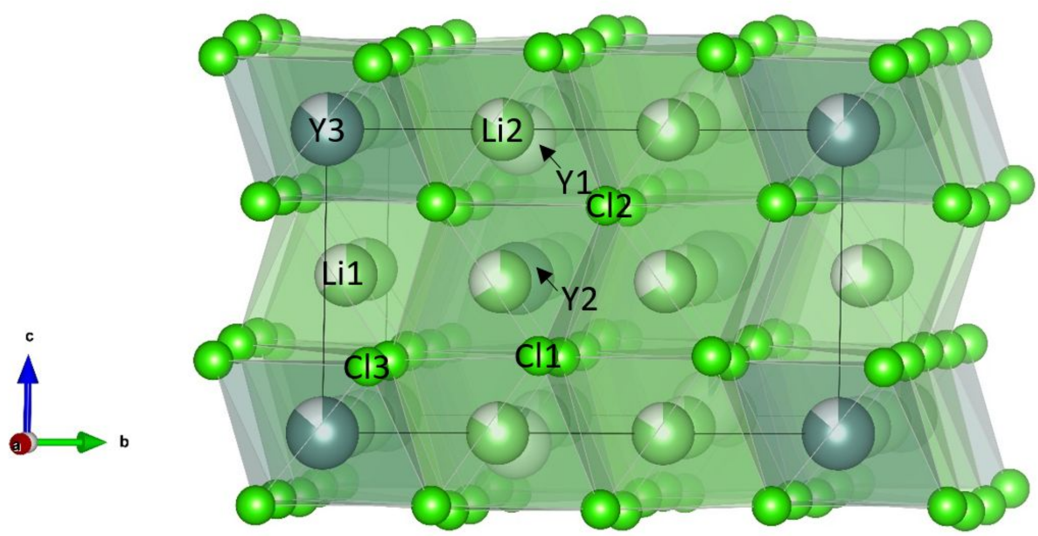

Figure S14: Crystal structure of Li_3YCl_6 visualized.

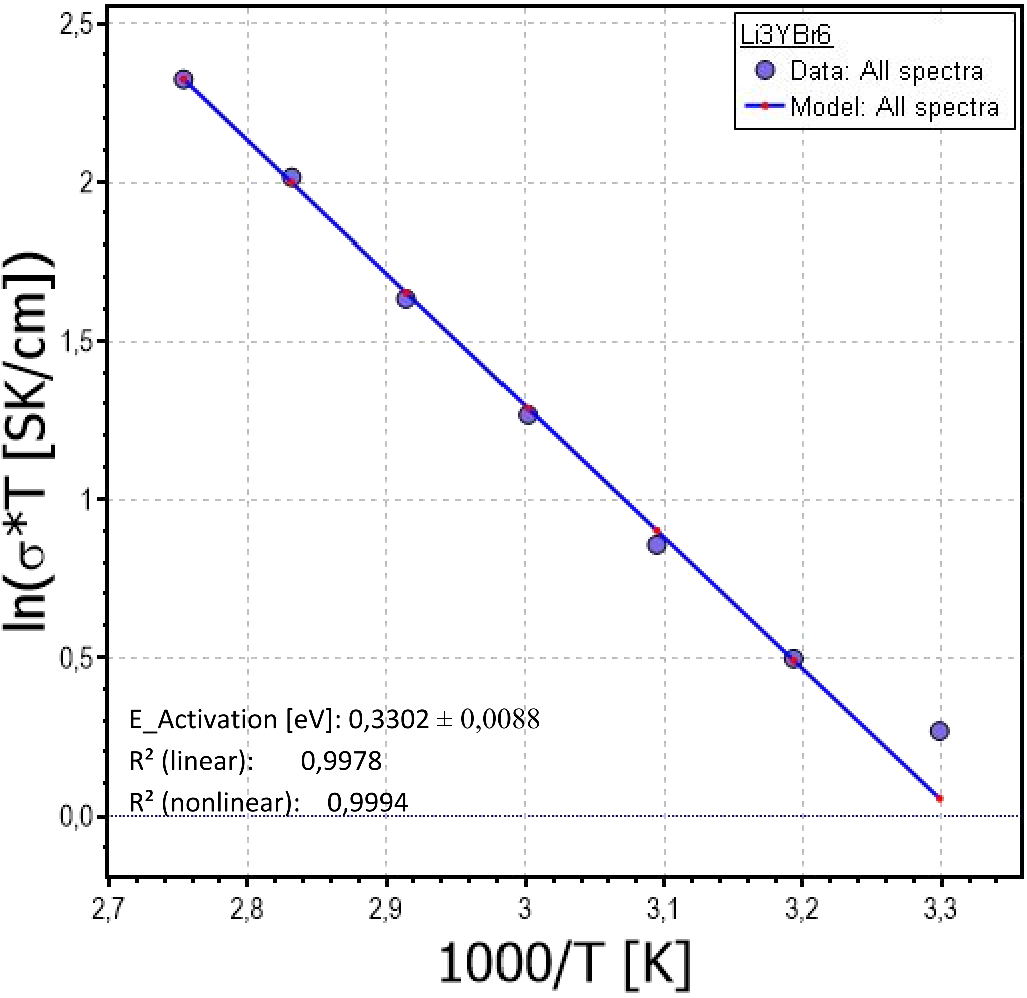

Figure S15: Arrhenius relationship of Li_3YBr_6 as measured by AC-impedance spectroscopy.

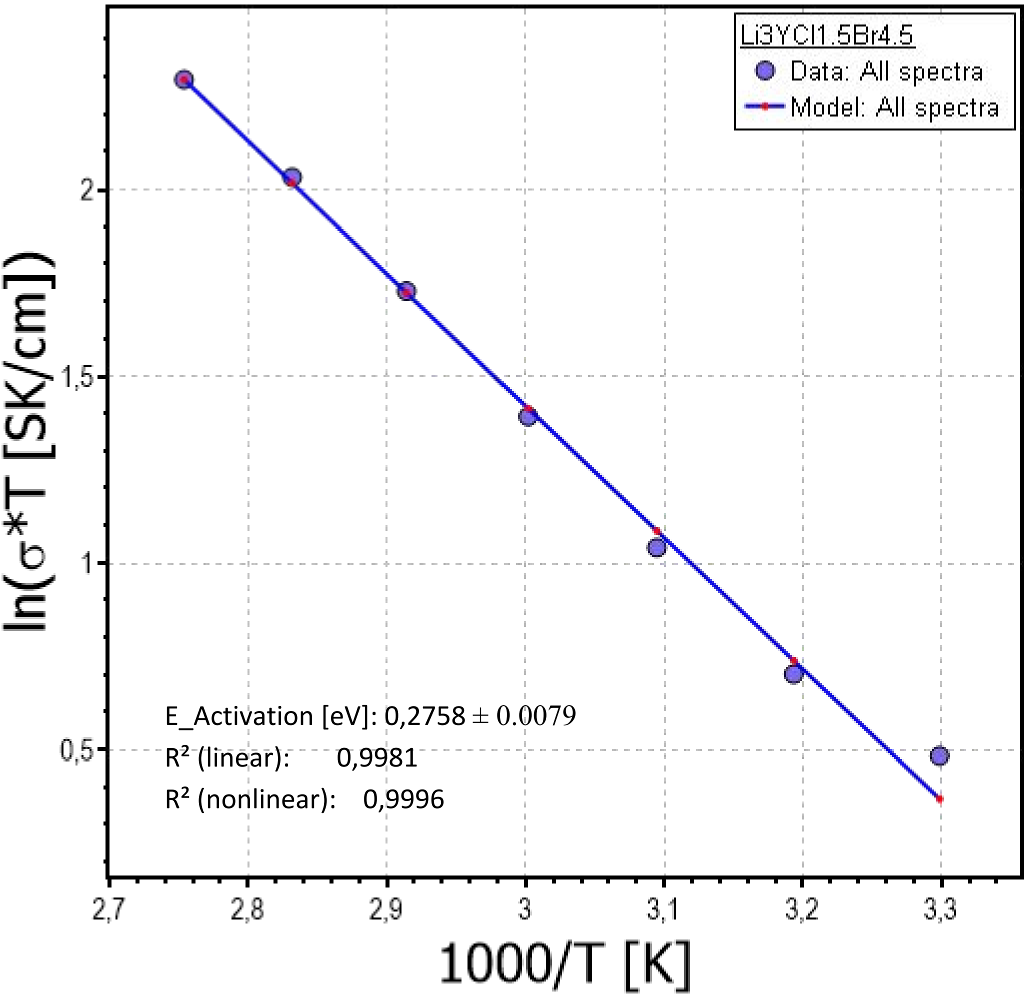

Figure S16: Arrhenius relationship of $Li_3YBr_{4.5}Cl_{1.5}$ as measured by AC-impedance spectroscopy.

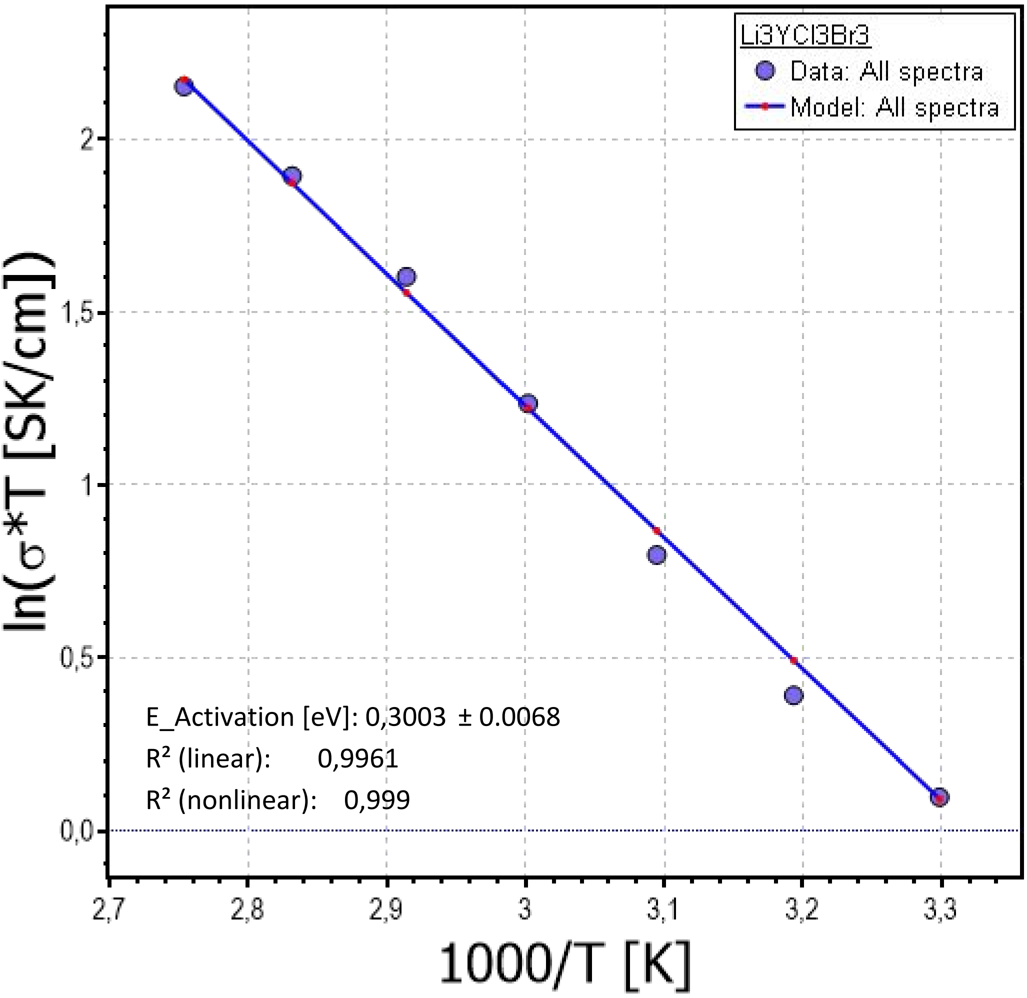

Figure S17: Arrhenius relationship of $Li_3YBr_3Cl_3$ as measured by AC-impedance spectroscopy.

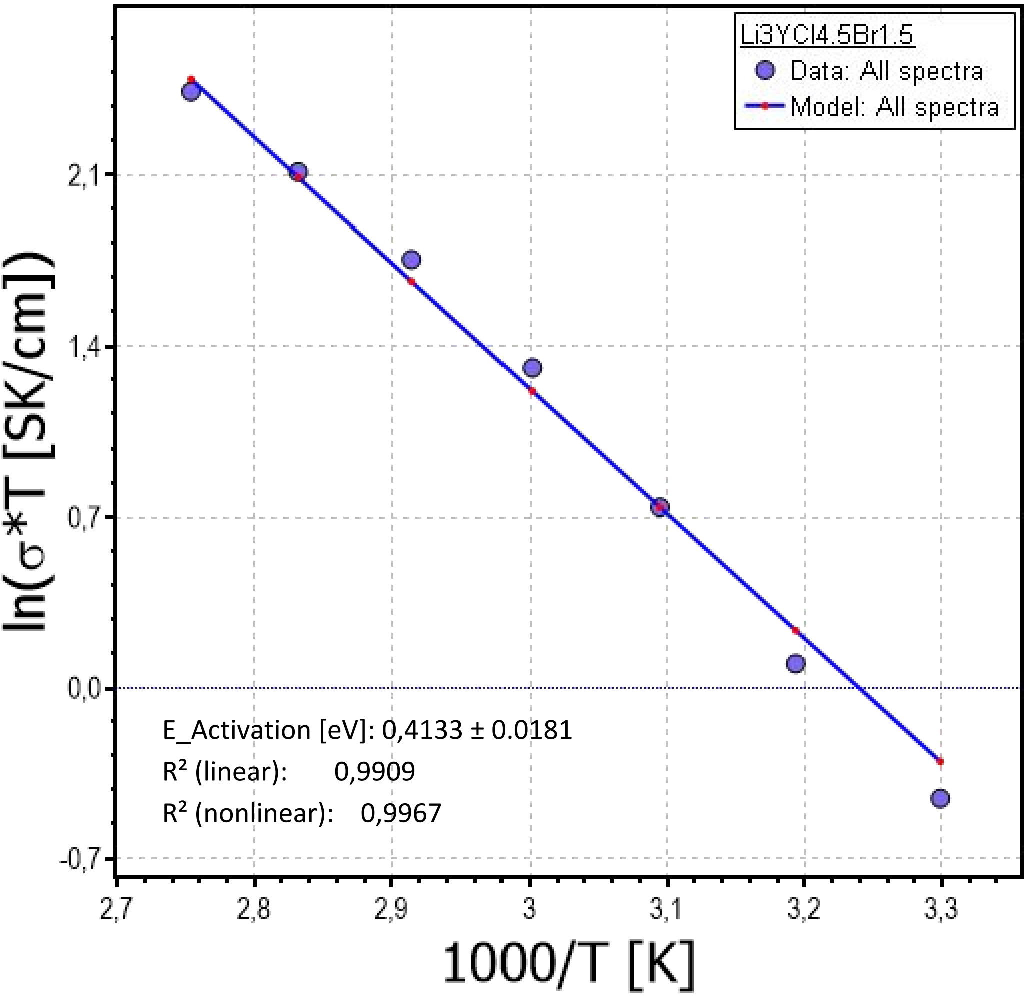

Figure S18: Arrhenius relationship of $Li_3YBr_{1.5}Cl_{4.5}$ as measured by AC-impedance spectroscopy.

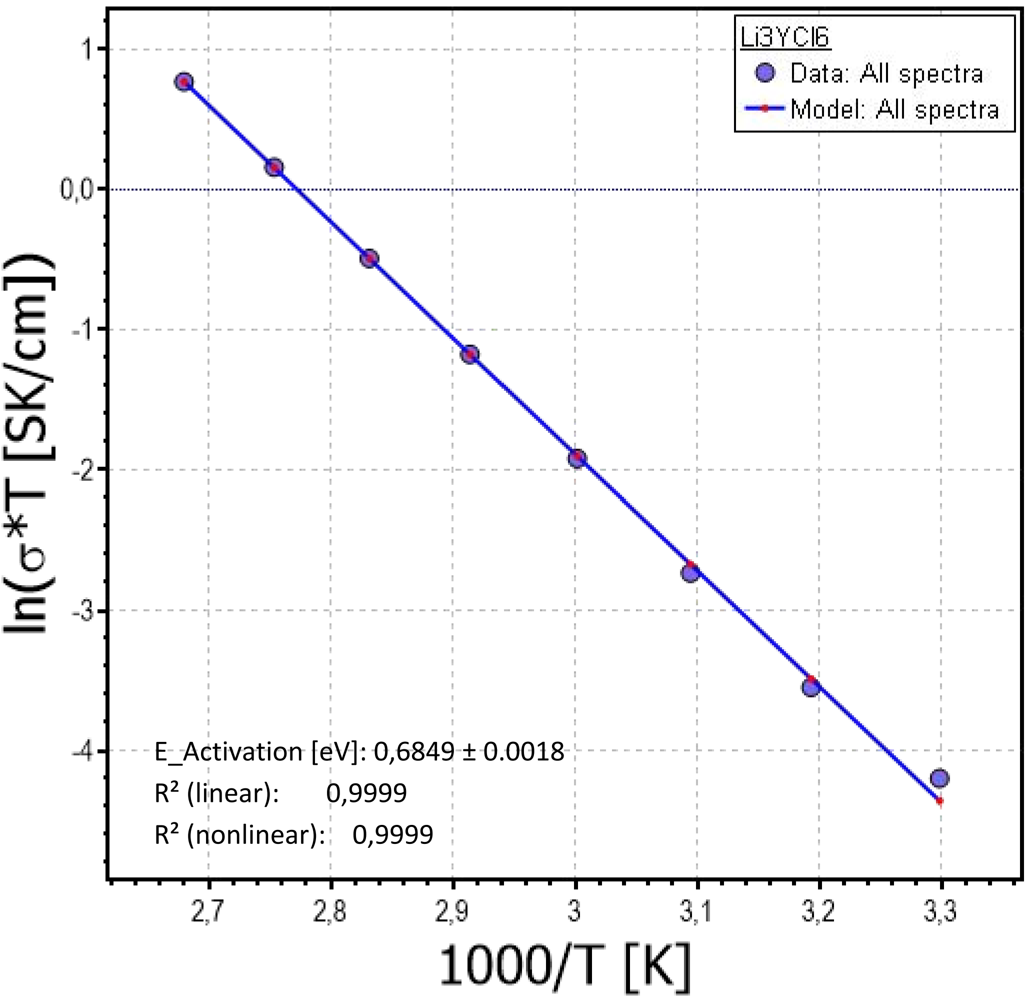

Figure S19: Arrhenius relationship of Li_3YCl_6 as measured by AC-impedance spectroscopy.

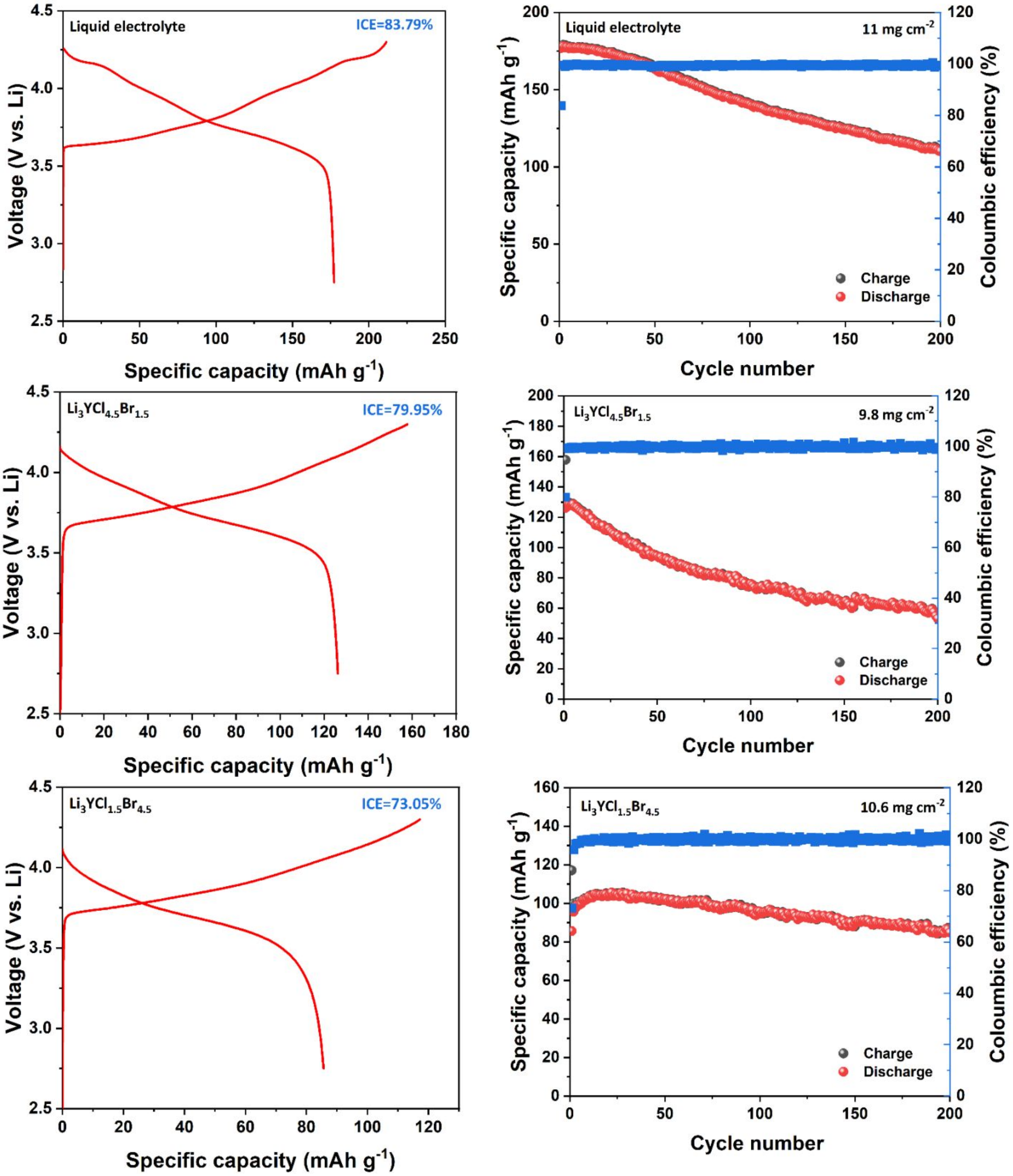

Figure S20: First cycle data (left) and long term cycling performance (right) of full cells using NCM811 – Halide SE – Sulphide Argyrodite – Li-In. Top: reference cathode performance in liquid electrolyte. Middle: $Li_3YCl_{4.5}Br_{1.5}$ as halide solid electrolyte. Bottom: $Li_3YCl_{1.5}Br_{4.5}$ as halide solid electrolyte. The batteries were cycled at C/10. The authors note that long-term cycling of these electrolytes also depend on the halide-sulphide solid electrolyte interface, as well as on mechanical properties and the reached capacities.

References

(1) Liu, Z.; Ma, S.; Liu, J.; Xiong, S.; Ma, Y.; Chen, H. High Ionic Conductivity Achieved in $Li_3Y(Br_3Cl_3)$ Mixed Halide Solid Electrolyte via Promoted Diffusion Pathways and Enhanced Grain Boundary. *ACS Energy Lett.* **2021**, *6* (1), 298–304.

(2) Plass, M. A.; Bette, S.; Dinnebier, R. E.; Lotsch, B. V. Enhancement of Superionic Conductivity by Halide Substitution in Strongly Stacking Faulted Li3HoBr6– xIx Phases. *Chem. Mater.* **2022**, 34 (7), 3227-3235.

(3) Sebti, E.; Evans, H. A.; Chen, H.; Richardson, P. M.; White, K. M.; Giovine, R.; Koirala, K. P.; Xu, Y.; Gonzalez-Correa, E.; Wang, C.; Brown, C. M.; Cheetham, A. K.; Canepa, P.; Clément, R. J. Stacking Faults Assist Lithium-Ion Conduction in a Halide-Based Superionic Conductor. *J. Am. Chem. Soc.* **2022**, *144*(13), 5795-5811.
