# Supplementary material for: Investigation of Structure, Ionic Conductivity, and Electrochemical Stability of Halogen Substitution in Solid-State Ion Conductor Li3YBrxCl6–x
Source: J Phys Chem C Nanomater Interfaces. 2022 Dec 16;127(1):125–32. doi: 10.1021/acs.jpcc.2c07910 (PMC9841563; doi:10.1021/acs.jpcc.2c07910)
Supplement: Supplementary file 2 — jp2c07910_si_002.pdf [file jp2c07910_si_002.pdf]

RelaxIS 3.0.20.16 - Report

Datasource: LYBC-HT_30C.txt_1

Circuit: I-(R)-(P)

Type	Value
**Temperature:**	30,000000
**Free variable:**	N/A
**DC Voltage:**	N/A
**AC Voltage:**	N/A
**Time:**	N/A
**Harmonic:**	N/A
**Free Variable 2:**	N/A
**Area:**	0,7853982
**Thickness:**	0,0584900

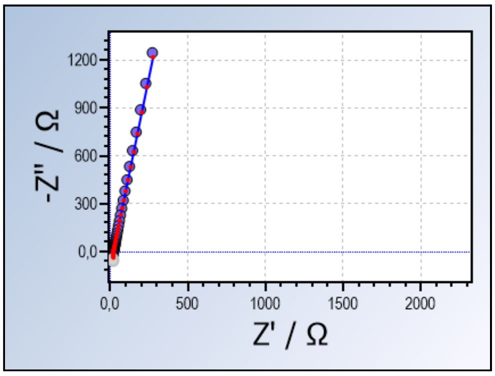

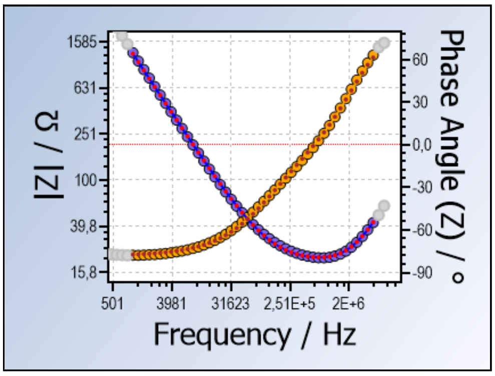

Fit Parameters:

Fix?	Name	Value	Error (Relative)
	Inductance 1	1,33E-006	9,04E-009 (0,6813741 %)
	Resistance 1	20,449686	0,0821332 (0,4016354 %)
	CPE Q 1	4,05E-007	6,67E-009 (1,6454085 %)
	CPE Alpha 1	0,8680582	0,0014698 (0,1693182 %)

RelaxIS 3.0.20.16 - Report

Datasource: LYBC-HT_40C.txt_1

Circuit: I-(R)-(P)

Type	Value
**Temperature:**	40,000000
**Free variable:**	N/A
**DC Voltage:**	N/A
**AC Voltage:**	N/A
**Time:**	N/A
**Harmonic:**	N/A
**Free Variable 2:**	N/A
**Area:**	0,7853982
**Thickness:**	0,0584900

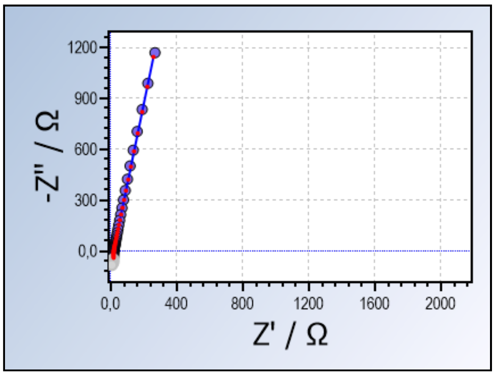

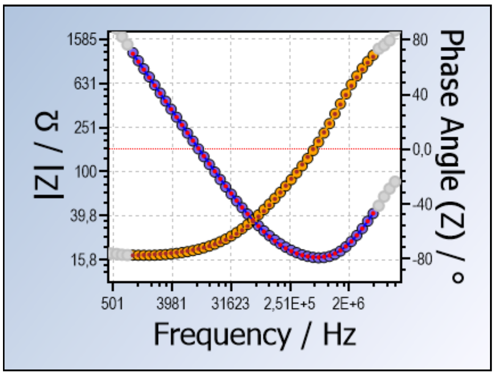

Fit Parameters:

Fix?	Name	Value	Error (Relative)
	Inductance 1	1,36E-006	7,53E-009 (0,5552356 %)
	Resistance 1	15,773357	0,0628715 (0,3985928 %)
	CPE Q 1	4,33E-007	6,40E-009 (1,4769479 %)
	CPE Alpha 1	0,8674757	0,0013073 (0,1506996 %)

RelaxIS 3.0.20.16 - Report

Datasource: LYBC-HT_50C.txt_1

Circuit: I-(R)-(P)

Type	Value
**Temperature:**	50,000000
**Free variable:**	N/A

**DC Voltage:**	N/A
**AC Voltage:**	N/A
**Time:**	N/A
**Harmonic:**	N/A
**Free Variable 2:**	N/A
**Area:**	0,7853982
**Thickness:**	0,0584900

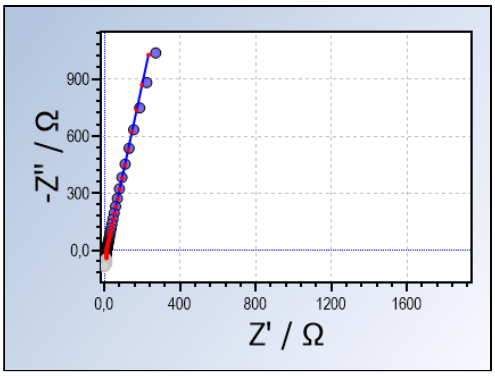

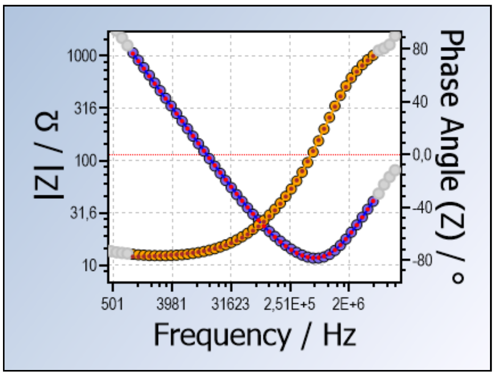

FIT PARAMETERS:

Fix?	Name	Value	Error (Relative)
	Inductance 1	1,38E-006	6,26E-009 (0,4521988 %)
	Resistance 1	10,834973	0,0457904 (0,4226164 %)
	CPE Q 1	4,96E-007	6,65E-009 (1,3403915 %)
	CPE Alpha 1	0,8643185	0,0011718 (0,1355761 %)

RelaxIS 3.0.20.16 - Report

Datasource: LYBC-HT_60C.txt_1

Circuit: I-(R)-(P)

Type	Value
**Temperature:**	60,000000
**Free variable:**	N/A
**DC Voltage:**	N/A
**AC Voltage:**	N/A
**Time:**	N/A
**Harmonic:**	N/A
**Free Variable 2:**	N/A
**Area:**	0,7853982
**Thickness:**	0,0584900

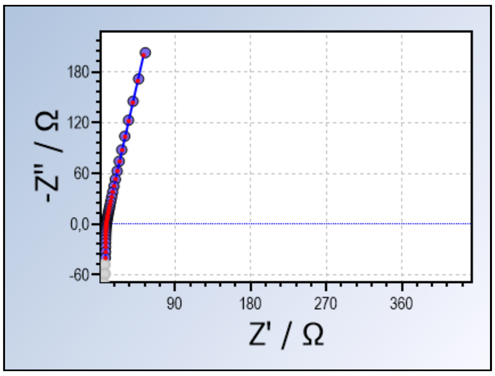

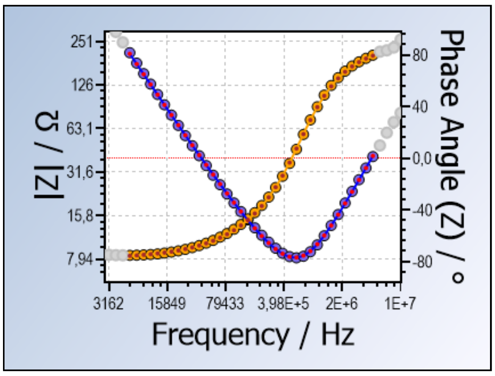

FIT PARAMETERS:

Fix?	Name	Value	Error (Relative)
	Inductance 1	1,40E-006	3,75E-009 (0,2679009 %)
	Resistance 1	7,2078225	0,0252281 (0,3500094 %)
	CPE Q 1	6,10E-007	8,37E-009 (1,3707727 %)
	CPE Alpha 1	0,8580490	0,0011096 (0,1293217 %)

RelaxIS 3.0.20.16 - Report

Datasource: LYBC-HT_70C.txt_1

Circuit: I-(R)-(P)

Type	Value
**Temperature:**	70,000000
**Free variable:**	N/A
**DC Voltage:**	N/A
**AC Voltage:**	N/A
**Time:**	N/A
**Harmonic:**	N/A
**Free Variable 2:**	N/A
**Area:**	0,7853982
**Thickness:**	0,0584900

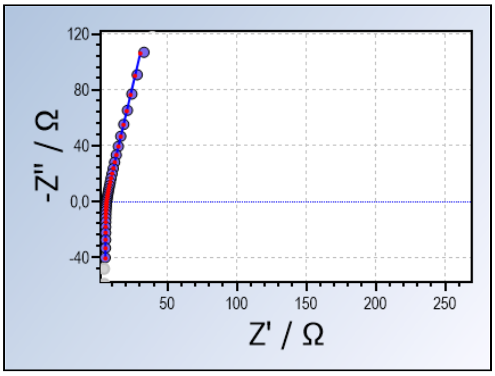

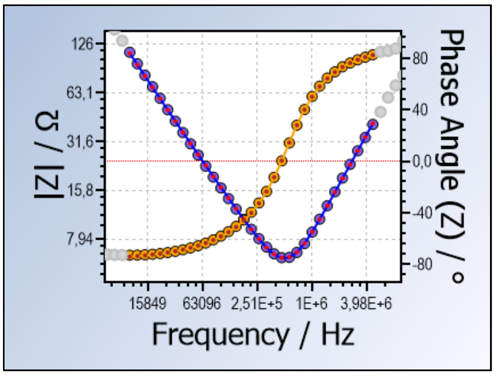

Fit Parameters:

Fix?	Name	Value	Error (Relative)
	Inductance 1	1,41E-006	2,79E-009 (0,1982960 %)
	Resistance 1	5,1409273	0,0173515 (0,3375169 %)
	CPE Q 1	7,52E-007	9,66E-009 (1,2852667 %)
	CPE Alpha 1	0,8515547	0,0010131 (0,1189709 %)

RelaxIS 3.0.20.16 - Report

Datasource: LYBC-HT_80C.txt_1

Circuit: I-(R)-(P)

Type	Value
**Temperature:**	80,000000
**Free variable:**	N/A
**DC Voltage:**	N/A
**AC Voltage:**	N/A
**Time:**	N/A
**Harmonic:**	N/A
**Free Variable 2:**	N/A
**Area:**	0,7853982
**Thickness:**	0,0584900

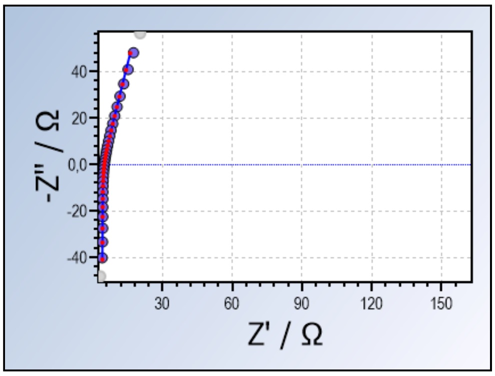

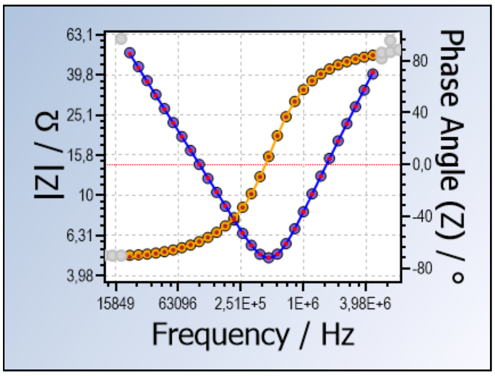

Fit Parameters:

Fix?	Name	Value	Error (Relative)
	Inductance 1	1,42E-006	2,77E-009 (0,1959435 %)
	Resistance 1	3,9574274	0,0170873 (0,4317777 %)
	CPE Q 1	9,47E-007	1,65E-008 (1,7385530 %)
	CPE Alpha 1	0,8431875	0,0013316 (0,1579272 %)

RelaxIS 3.0.20.16 - Report

Datasource: LYBC-HT_90C.txt_1

Circuit: I-(R)-(P)

Type	Value
**Temperature:**	90,000000
**Free variable:**	N/A
**DC Voltage:**	N/A
**AC Voltage:**	N/A
**Time:**	N/A
**Harmonic:**	N/A
**Free Variable 2:**	N/A
**Area:**	0,7853982
**Thickness:**	0,0584900

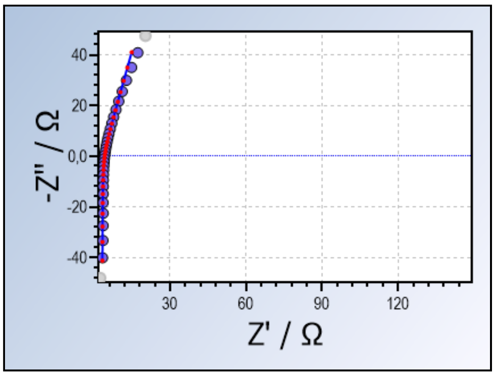

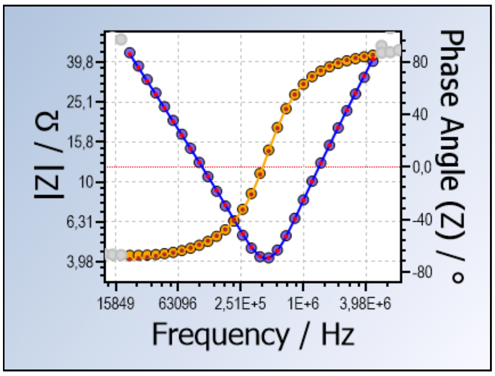

Fit Parameters:

Fix?	Name	Value	Error (Relative)
	Inductance 1	1,43E-006	5,86E-009 (0,4098898 %)
	Resistance 1	3,1406396	0,0343226 (1,0928534 %)
	CPE Q 1	1,37E-006	5,25E-008 (3,8285994 %)
	CPE Alpha 1	0,8243281	0,0029365 (0,3562332 %)
