# Supplementary material for: Investigation of Structure, Ionic Conductivity, and Electrochemical Stability of Halogen Substitution in Solid-State Ion Conductor Li3YBrxCl6–x
Source: J Phys Chem C Nanomater Interfaces. 2022 Dec 16;127(1):125–32. doi: 10.1021/acs.jpcc.2c07910 (PMC9841563; doi:10.1021/acs.jpcc.2c07910)
Supplement: Supplementary file 3 — jp2c07910_si_003.pdf [file jp2c07910_si_003.pdf]

RelaxIS 3.0.20.16 - Report

Datasource: LYB_30C.txt_1

Circuit: I-(R)-P

Type	Value
**Temperature:**	30,000000
**Free variable:**	N/A
**DC Voltage:**	N/A
**AC Voltage:**	N/A
**Time:**	N/A
**Harmonic:**	N/A
**Free Variable 2:**	N/A
**Area:**	N/A
**Thickness:**	N/A

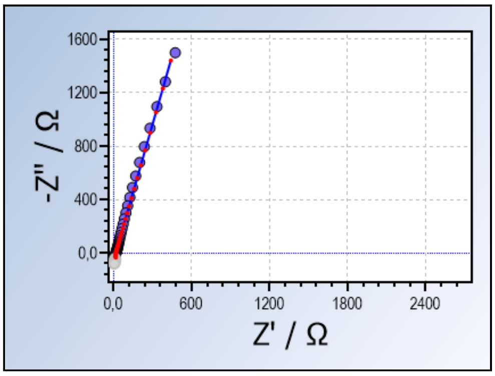

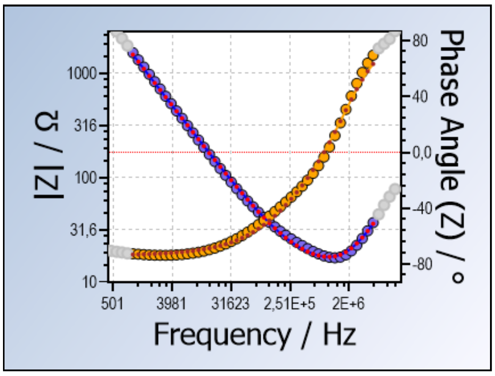

Fit Parameters:

Fix?	Name	Value	Error (Relative)
	Inductance 1	1,16E-006	2,14E-008 (1,8417179 %)
	Resistance 1	15,849283	0,2220044 (1,4007222 %)
	CPE Q 1	5,22E-007	2,29E-008 (4,3881904 %)
	CPE Alpha 1	0,8176592	0,0038151 (0,4665877 %)

RelaxIS 3.0.20.16 - Report

Datasource: LYB_40C.txt_1

Circuit: I-(R)-P

Type	Value
**Temperature:**	40,000000
**Free variable:**	N/A
**DC Voltage:**	N/A
**AC Voltage:**	N/A
**Time:**	N/A
**Harmonic:**	N/A
**Free Variable 2:**	N/A
**Area:**	N/A
**Thickness:**	N/A

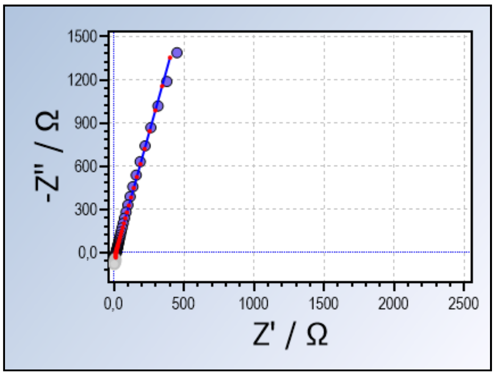

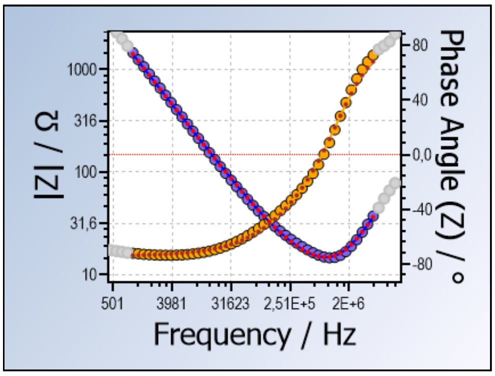

Fit Parameters:

Fix?	Name	Value	Error (Relative)
	Inductance 1	1,20E-006	1,67E-008 (1,3880687 %)
	Resistance 1	13,268290	0,1602863 (1,2080405 %)
	CPE Q 1	5,34E-007	1,90E-008 (3,5563299 %)
	CPE Alpha 1	0,8225023	0,0030791 (0,3743631 %)

RelaxIS 3.0.20.16 - Report

Datasource: LYB_50C.txt_1

Circuit: I-(R)-P

Type	Value
**Temperature:**	50,000000
**Free variable:**	N/A

**DC Voltage:**	N/A
**AC Voltage:**	N/A
**Time:**	N/A
**Harmonic:**	N/A
**Free Variable 2:**	N/A
**Area:**	N/A
**Thickness:**	N/A

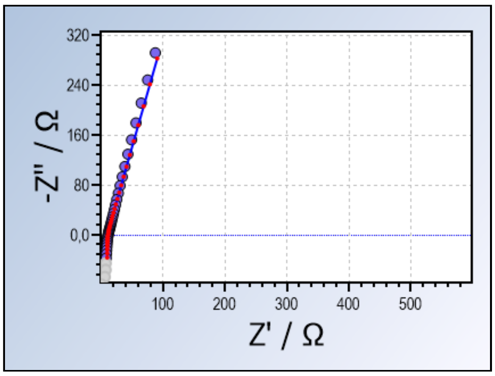

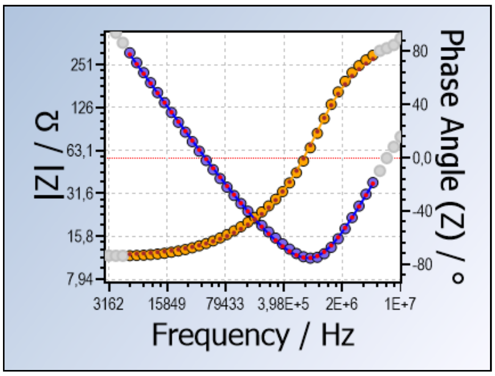

Fit Parameters:

Fix?	Name	Value	Error (Relative)
	Inductance 1	1,26E-006	1,12E-008 (0,8882804 %)
	Resistance 1	9,7136651	0,1001262 (1,0307769 %)
	CPE Q 1	6,17E-007	2,42E-008 (3,9208545 %)
	CPE Alpha 1	0,8224696	0,0031607 (0,3842921 %)

RelaxIS 3.0.20.16 - Report

Datasource: LYB_60C.txt_1

Circuit: I-(R)-P

Type	Value
**Temperature:**	60,000000
**Free variable:**	N/A
**DC Voltage:**	N/A
**AC Voltage:**	N/A
**Time:**	N/A
**Harmonic:**	N/A
**Free Variable 2:**	N/A
**Area:**	N/A
**Thickness:**	N/A

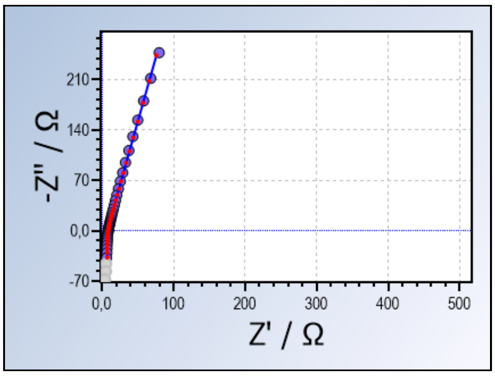

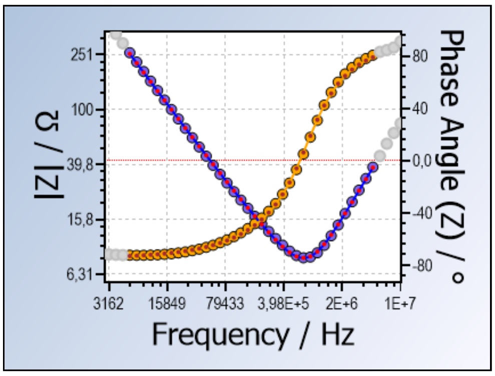

Fit Parameters:

Fix?	Name	Value	Error (Relative)
	Inductance 1	1,31E-006	6,49E-009 (0,4967353 %)
	Resistance 1	6,9317501	0,0503425 (0,7262600 %)
	CPE Q 1	7,01E-007	1,67E-008 (2,3855931 %)
	CPE Alpha 1	0,8245638	0,0019134 (0,2320509 %)

RelaxIS 3.0.20.16 - Report

Datasource: LYB_70C.txt_1

Circuit: I-(R)-P

Type	Value
**Temperature:**	70,000000
**Free variable:**	N/A
**DC Voltage:**	N/A
**AC Voltage:**	N/A
**Time:**	N/A
**Harmonic:**	N/A
**Free Variable 2:**	N/A
**Area:**	N/A
**Thickness:**	N/A

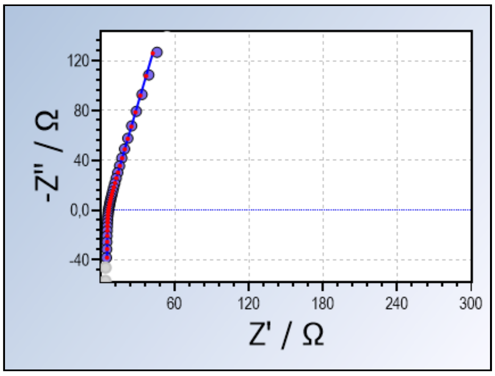

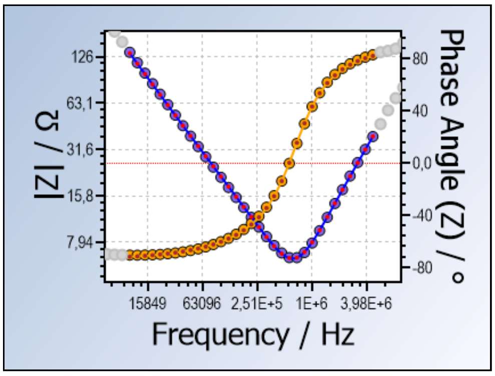

Fit Parameters:

Fix?	Name	Value	Error (Relative)
	Inductance 1	1,34E-006	3,81E-009 (0,2845498 %)
	Resistance 1	4,9126563	0,0270716 (0,5510575 %)
	CPE Q 1	9,10E-007	1,59E-008 (1,7455149 %)
	CPE Alpha 1	0,8174098	0,0013674 (0,1672835 %)

RelaxIS 3.0.20.16 - Report

Datasource: LYB_80C.txt_1

Circuit: I-(R)-P

Type	Value
**Temperature:**	80,000000
**Free variable:**	N/A
**DC Voltage:**	N/A
**AC Voltage:**	N/A
**Time:**	N/A
**Harmonic:**	N/A
**Free Variable 2:**	N/A
**Area:**	N/A
**Thickness:**	N/A

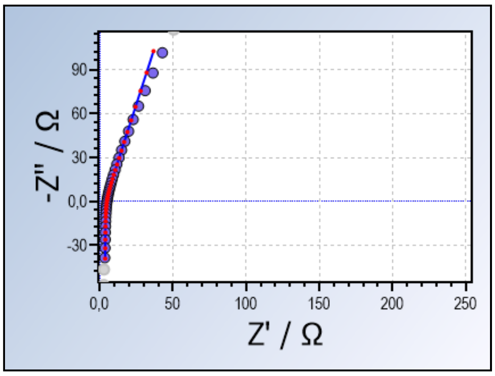

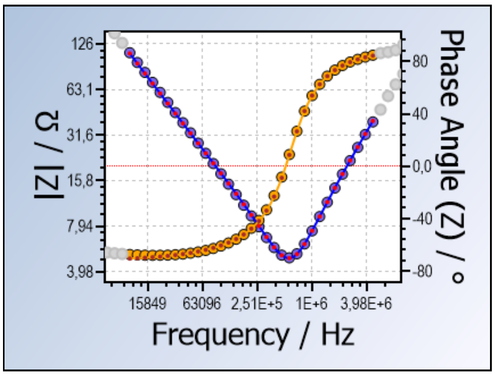

Fit Parameters:

Fix?	Name	Value	Error (Relative)
	Inductance 1	1,36E-006	5,37E-009 (0,3935548 %)
	Resistance 1	3,5228046	0,0347696 (0,9869857 %)
	CPE Q 1	1,33E-006	3,43E-008 (2,5812944 %)
	CPE Alpha 1	0,8008704	0,0020215 (0,2524104 %)

RelaxIS 3.0.20.16 - Report

Datasource: LYB_90C.txt_1

Circuit: I-(R)-P

Type	Value
**Temperature:**	90,000000
**Free variable:**	N/A
**DC Voltage:**	N/A
**AC Voltage:**	N/A
**Time:**	N/A
**Harmonic:**	N/A
**Free Variable 2:**	N/A
**Area:**	N/A
**Thickness:**	N/A

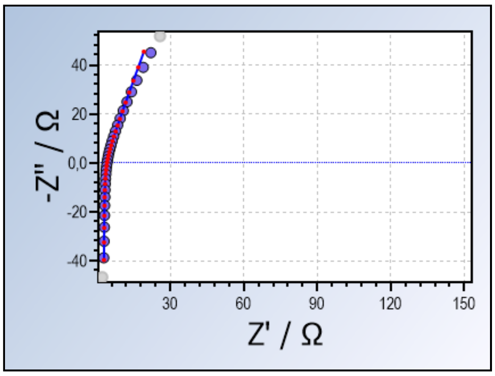

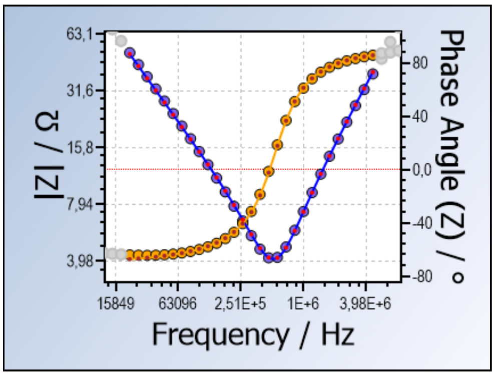

Fit Parameters:

Fix?	Name	Value	Error (Relative)
	Inductance 1	1,38E-006	5,91E-009 (0,4281810 %)
	Resistance 1	2,6445241	0,0393790 (1,4890751 %)
	CPE Q 1	2,07E-006	7,99E-008 (3,8552213 %)
	CPE Alpha 1	0,7788582	0,0029509 (0,3788735 %)
