# Supplementary material for: Investigation of Structure, Ionic Conductivity, and Electrochemical Stability of Halogen Substitution in Solid-State Ion Conductor Li3YBrxCl6–x
Source: J Phys Chem C Nanomater Interfaces. 2022 Dec 16;127(1):125–32. doi: 10.1021/acs.jpcc.2c07910 (PMC9841563; doi:10.1021/acs.jpcc.2c07910)
Supplement: Supplementary file 4 — jp2c07910_si_004.pdf [file jp2c07910_si_004.pdf]

RelaxIS 3.0.20.16 - Report

Datasource: LYC_30C.txt_1

Circuit: I-(R)(P)-P

Type	Value
**Temperature:**	30,000000
**Free variable:**	N/A
**DC Voltage:**	N/A
**AC Voltage:**	N/A
**Time:**	N/A
**Harmonic:**	N/A
**Free Variable 2:**	N/A
**Area:**	0,7854000
**Thickness:**	0,0584900

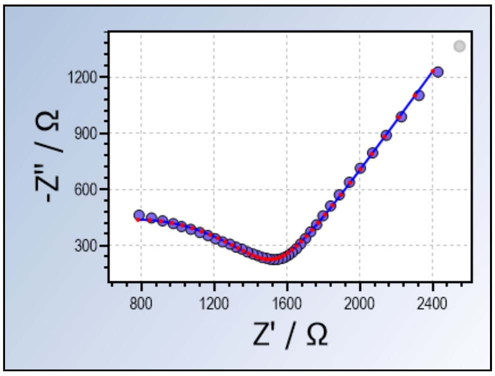

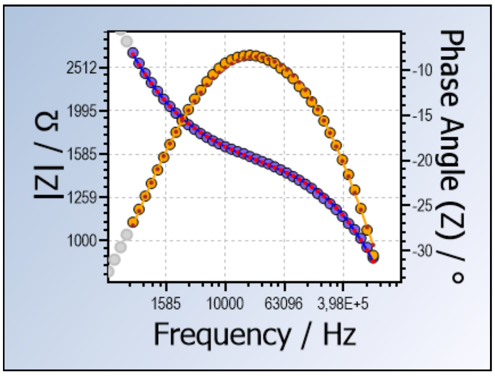

Fit Parameters:

Fix?	Name	Value	Error (Relative)
X	Inductance 1	0,0	0,0 (NaN %)
	Resistance 1	1500,6060	6,8433491 (0,4560390 %)
	CPE Q 1	2,22E-008	1,69E-009 (7,6000319 %)
	CPE Alpha 1	0,6561676	0,0048870 (0,7447766 %)
	CPE Q 2	5,10E-006	2,02E-007 (3,9689202 %)
	CPE Alpha 2	0,5947119	0,0048695 (0,8187931 %)

RelaxIS 3.0.20.16 - Report

Datasource: LYC_40C.txt_1

Circuit: I-(R)(P)-P

Type	Value
**Temperature:**	40,000000
**Free variable:**	N/A
**DC Voltage:**	N/A
**AC Voltage:**	N/A
**Time:**	N/A
**Harmonic:**	N/A
**Free Variable 2:**	N/A
**Area:**	0,7854000
**Thickness:**	0,0584900

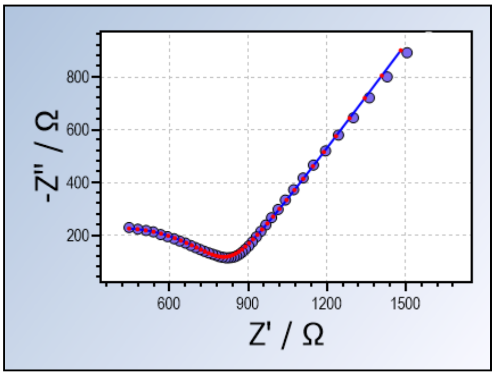

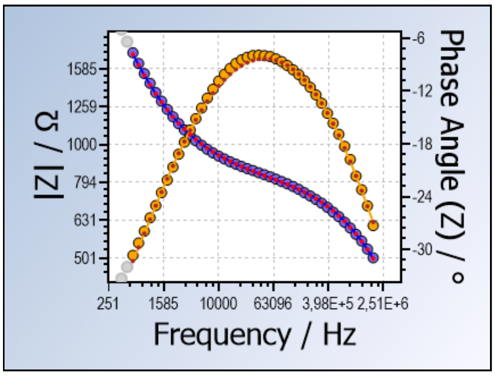

Fit Parameters:

Fix?	Name	Value	Error (Relative)
X	Inductance 1	0,0	0,0 (NaN %)
	Resistance 1	810,23377	2,9731009 (0,3669436 %)
	CPE Q 1	3,71E-008	2,66E-009 (7,1570006 %)
	CPE Alpha 1	0,6331920	0,0044260 (0,6990051 %)
	CPE Q 2	7,17E-006	1,93E-007 (2,6881314 %)
	CPE Alpha 2	0,5902428	0,0032300 (0,5472356 %)

RelaxIS 3.0.20.16 - Report

Datasource: LYC_50C.txt_1

Circuit: I-(R)(P)-P

Type	Value
**Temperature:**	50,000000
**Free variable:**	N/A
**DC Voltage:**	N/A
**AC Voltage:**	N/A
**Time:**	N/A
**Harmonic:**	N/A
**Free Variable 2:**	N/A
**Area:**	0,7854000
**Thickness:**	0,0584900

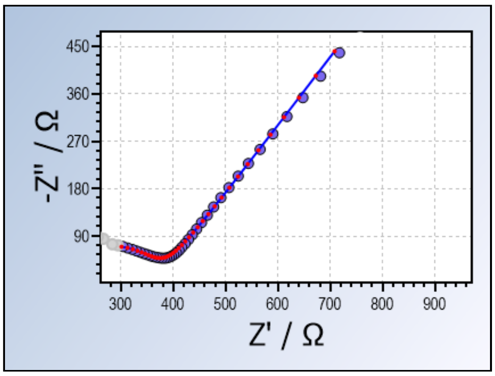

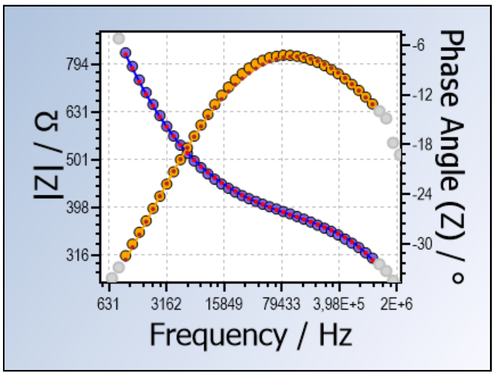

Fit Parameters:

Fix?	Name	Value	Error (Relative)
X	Inductance 1	4,12E-006	0,0 (0,0 %)
	Resistance 1	369,60995	1,3995194 (0,3786477 %)
	CPE Q 1	9,74E-009	1,44E-009 (14,747680 %)
	CPE Alpha 1	0,7381393	0,0093811 (1,2709174 %)
	CPE Q 2	1,10E-005	2,97E-007 (2,6943795 %)
	CPE Alpha 2	0,5825376	0,0030411 (0,5220444 %)

RelaxIS 3.0.20.16 - Report

Datasource: LYC_60C.txt_1

Circuit: I-(R)(P)-P

Type	Value
**Temperature:**	60,000000
**Free variable:**	N/A
**DC Voltage:**	N/A
**AC Voltage:**	N/A
**Time:**	N/A
**Harmonic:**	N/A
**Free Variable 2:**	N/A
**Area:**	0,7854000
**Thickness:**	0,0584900

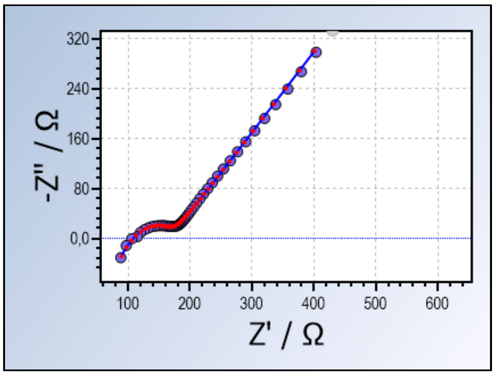

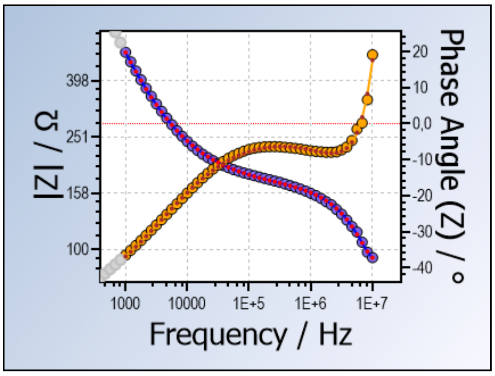

Fit Parameters:

Fix?	Name	Value	Error (Relative)
	Inductance 1	1,33E-006	1,38E-008 (1,0439667 %)
	Resistance 1	169,08253	0,6235830 (0,3688039 %)
	CPE Q 1	1,35E-008	1,82E-009 (13,499154 %)
	CPE Alpha 1	0,7204568	0,0077847 (1,0805297 %)
	CPE Q 2	1,63E-005	4,29E-007 (2,6352973 %)
	CPE Alpha 2	0,5816558	0,0028309 (0,4867011 %)

RelaxIS 3.0.20.16 - Report

Datasource: LYC_70C.txt_1

Circuit: I-(R)(P)-P

Type	Value
**Temperature:**	70,000000
**Free variable:**	N/A
**DC Voltage:**	N/A
**AC Voltage:**	N/A
**Time:**	N/A
**Harmonic:**	N/A
**Free Variable**	N/A

2:	
**Area:**	0,7854000
**Thickness:**	0,0584900

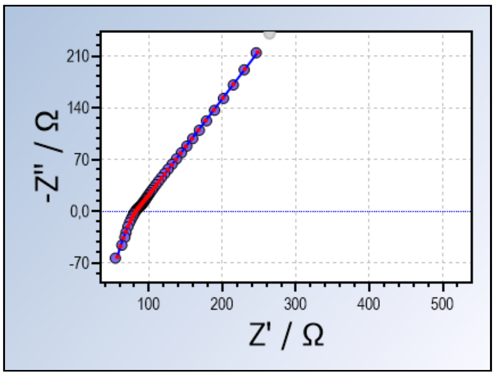

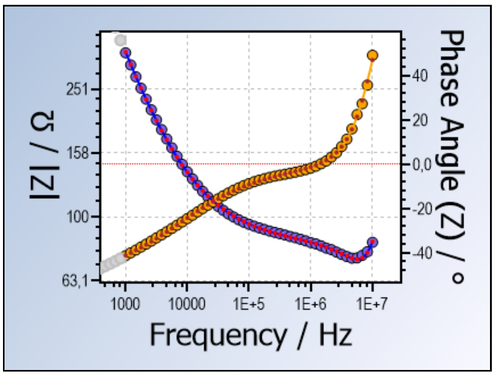

Fit Parameters:

Fix?	Name	Value	Error (Relative)
	Inductance 1	1,45E-006	1,36E-008 (0,9321554 %)
	Resistance 1	82,869704	0,2671902 (0,3224221 %)
	CPE Q 1	2,54E-009	5,71E-010 (22,466658 %)
	CPE Alpha 1	0,8299016	0,0134264 (1,6178360 %)
	CPE Q 2	2,25E-005	4,63E-007 (2,0561537 %)
	CPE Alpha 2	0,5829926	0,0021466 (0,3682084 %)

RelaxIS 3.0.20.16 - Report

Datasource: LYC_80C.txt_1

Circuit: I-(R)(P)-P

Type	Value
**Temperature:**	80,000000
**Free variable:**	N/A
**DC Voltage:**	N/A
**AC Voltage:**	N/A
**Time:**	N/A
**Harmonic:**	N/A
**Free Variable 2:**	N/A
**Area:**	0,7854000
**Thickness:**	0,0584900

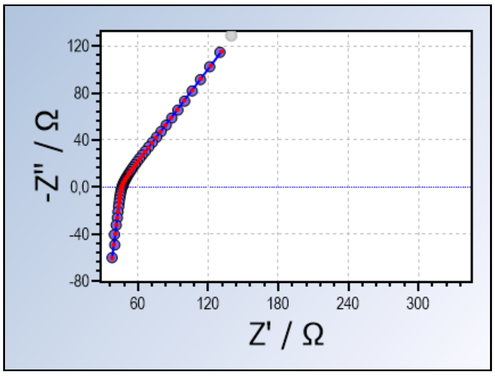

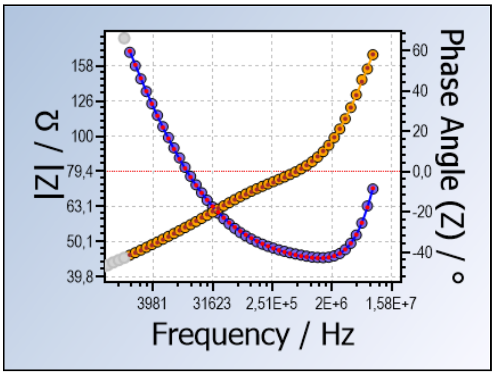

Fit Parameters:

Fix?	Name	Value	Error (Relative)
	Inductance 1	1,44E-006	1,33E-008 (0,9218182 %)
	Resistance 1	43,022785	0,0981427 (0,2281180 %)
	CPE Q 1	3,68E-010	1,00E-010 (27,153946 %)
	CPE Alpha 1	0,9559427	0,0176119 (1,8423620 %)
	CPE Q 2	2,98E-005	4,42E-007 (1,4842659 %)
	CPE Alpha 2	0,5838634	0,0014649 (0,2508936 %)

RelaxIS 3.0.20.16 - Report

Datasource: LYC_90C.txt_1

Circuit: I-(R)(P)-P

Type	Value
**Temperature:**	90,000000
**Free variable:**	N/A
**DC Voltage:**	N/A
**AC Voltage:**	N/A
**Time:**	N/A
**Harmonic:**	N/A
**Free Variable**	N/A

**2:**	
**Area:**	0,7854000
**Thickness:**	0,0584900

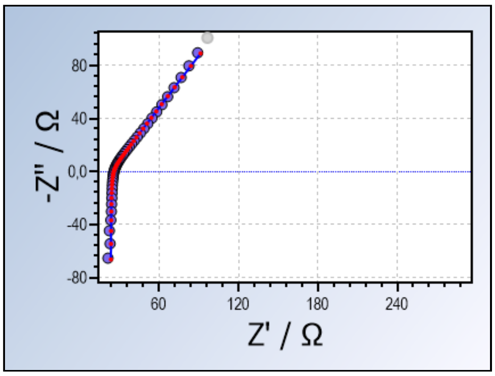

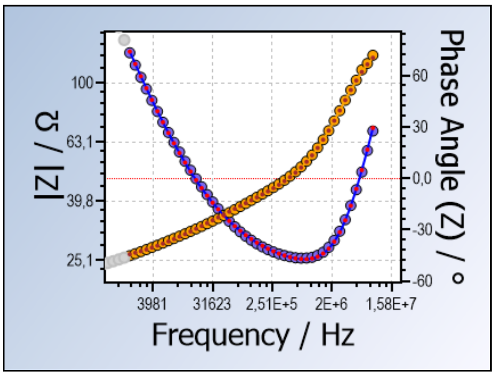

Fit Parameters:

Fix?	Name	Value	Error (Relative)
	Inductance 1	1,29E-006	4,25E-009 (0,3294012 %)
	Resistance 1	23,092017	0,0613401 (0,2656334 %)
X	CPE Q 1	0,0	0,0 (NaN %)
X	CPE Alpha 1	1,0000000	0,0 (0,0 %)
	CPE Q 2	3,82E-005	6,64E-007 (1,7378009 %)
	CPE Alpha 2	0,5848399	0,0016462 (0,2814872 %)

RelaxIS 3.0.20.16 - Report

Datasource: LYC_100C.txt_1

Circuit: I-(R)(P)-P

Type	Value
**Temperature:**	100,00000
**Free variable:**	N/A
**DC Voltage:**	N/A
**AC Voltage:**	N/A
**Time:**	N/A
**Harmonic:**	N/A
**Free Variable 2:**	N/A
**Area:**	0,7854000
**Thickness:**	0,0584900

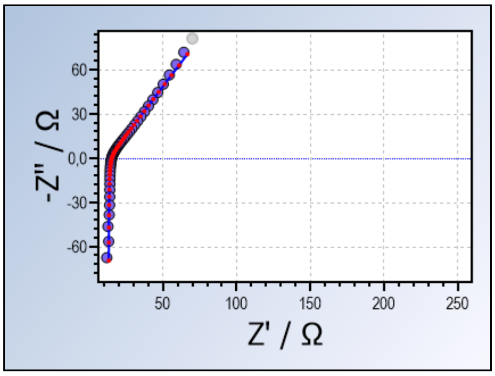

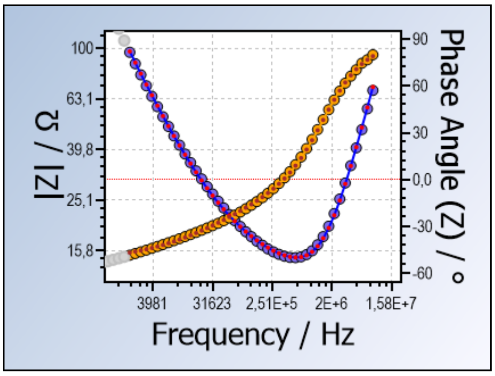

Fit Parameters:

Fix?	Name	Value	Error (Relative)
	Inductance 1	1,34E-006	4,67E-009 (0,3478952 %)
	Resistance 1	12,892716	0,0521779 (0,4047081 %)
X	CPE Q 1	0,0	0,0 (NaN %)
X	CPE Alpha 1	1,0000000	0,0 (0,0 %)
	CPE Q 2	4,75E-005	9,60E-007 (2,0218453 %)
	CPE Alpha 2	0,5866495	0,0018949 (0,3230081 %)
