# Supplementary material for: Investigation of Structure, Ionic Conductivity, and Electrochemical Stability of Halogen Substitution in Solid-State Ion Conductor Li3YBrxCl6–x
Source: J Phys Chem C Nanomater Interfaces. 2022 Dec 16;127(1):125–32. doi: 10.1021/acs.jpcc.2c07910 (PMC9841563; doi:10.1021/acs.jpcc.2c07910)
Supplement: Supplementary file 5 — jp2c07910_si_005.pdf [file jp2c07910_si_005.pdf]

RelaxIS 3.0.20.16 - Report

Datasource: LYB4.5C1.5_30C.txt_1

Circuit: I-(R)-(P)

Type	Value
**Temperature:**	30,000000
**Free variable:**	N/A
**DC Voltage:**	N/A
**AC Voltage:**	N/A
**Time:**	N/A
**Harmonic:**	N/A
**Free Variable 2:**	N/A
**Area:**	N/A
**Thickness:**	N/A

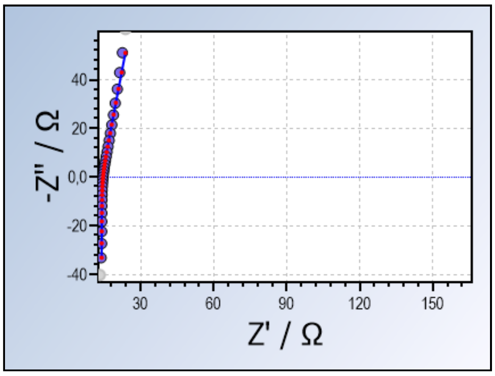

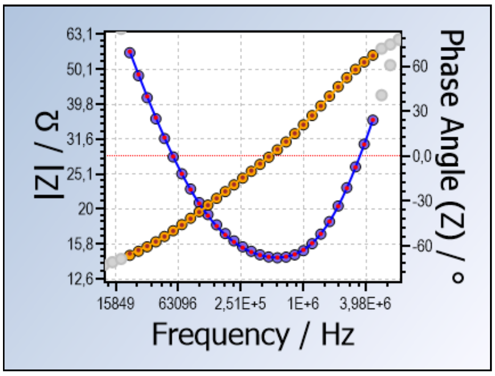

Fit Parameters:

Fix?	Name	Value	Error (Relative)
	Inductance 1	1,15E-006	3,03E-009 (0,2634081 %)
	Resistance 1	13,895778	0,0279537 (0,2011669 %)
	CPE Q 1	5,90E-007	1,20E-008 (2,0265283 %)
	CPE Alpha 1	0,8792852	0,0015938 (0,1812610 %)

RelaxIS 3.0.20.16 - Report

Datasource: LYB4.5C1.5_40C.txt_1

Circuit: I-(R)-(P)

Type	Value
**Temperature:**	40,000000
**Free variable:**	N/A
**DC Voltage:**	N/A
**AC Voltage:**	N/A
**Time:**	N/A
**Harmonic:**	N/A
**Free Variable 2:**	N/A
**Area:**	N/A
**Thickness:**	N/A

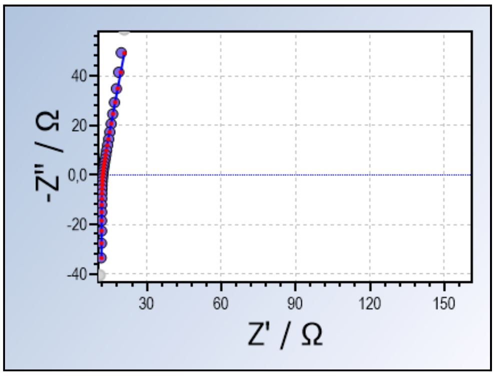

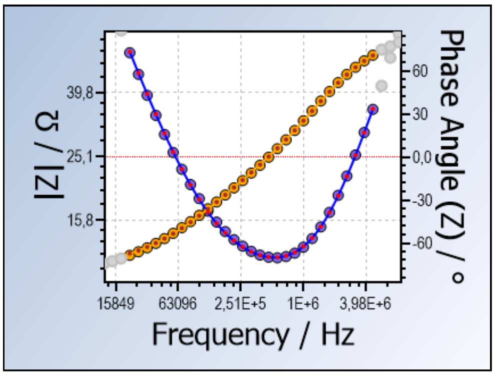

Fit Parameters:

Fix?	Name	Value	Error (Relative)
	Inductance 1	1,16E-006	3,06E-009 (0,2628626 %)
	Resistance 1	11,538652	0,0263966 (0,2287670 %)
	CPE Q 1	5,98E-007	1,25E-008 (2,0840452 %)
	CPE Alpha 1	0,8812140	0,0016329 (0,1853025 %)

RelaxIS 3.0.20.16 - Report

Datasource: LYB4.5C1.5_50C.txt_1

Circuit: I-(R)-(P)

Type	Value
**Temperature:**	50,000000
**Free variable:**	N/A

**DC Voltage:**	N/A
**AC Voltage:**	N/A
**Time:**	N/A
**Harmonic:**	N/A
**Free Variable 2:**	N/A
**Area:**	N/A
**Thickness:**	N/A

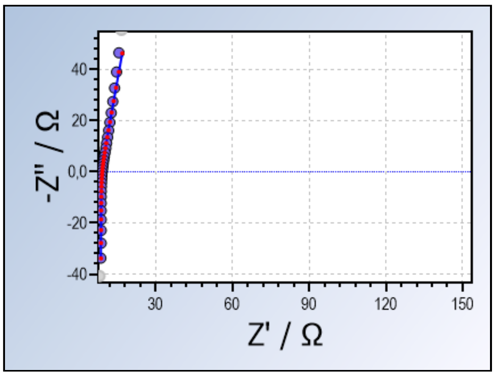

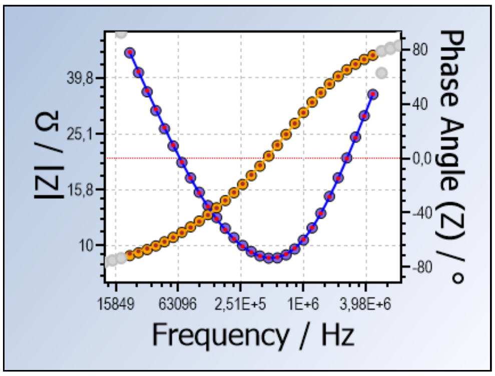

Fit Parameters:

Fix?	Name	Value	Error (Relative)
	Inductance 1	1,17E-006	3,40E-009 (0,2896343 %)
	Resistance 1	8,4852757	0,0261613 (0,3083141 %)
	CPE Q 1	6,01E-007	1,43E-008 (2,3822189 %)
	CPE Alpha 1	0,8860578	0,0018551 (0,2093670 %)

RelaxIS 3.0.20.16 - Report

Datasource: LYB4.5C1.5_60C.txt_1

Circuit: I-(R)-(P)

Type	Value
**Temperature:**	60,000000
**Free variable:**	N/A
**DC Voltage:**	N/A
**AC Voltage:**	N/A
**Time:**	N/A
**Harmonic:**	N/A
**Free Variable 2:**	N/A
**Area:**	N/A
**Thickness:**	N/A

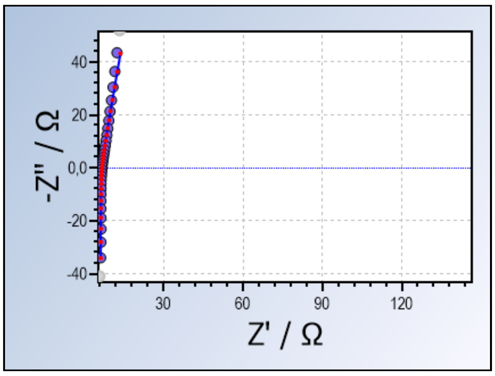

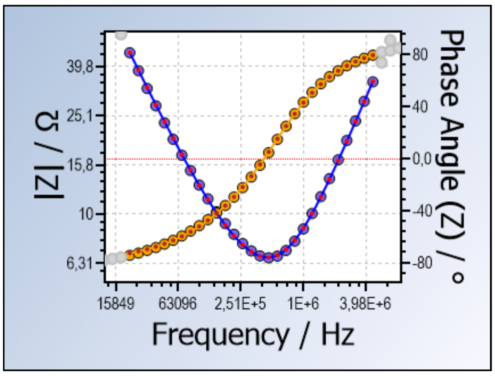

Fit Parameters:

Fix?	Name	Value	Error (Relative)
	Inductance 1	1,18E-006	3,68E-009 (0,3112124 %)
	Resistance 1	6,1532417	0,0249883 (0,4061005 %)
	CPE Q 1	6,07E-007	1,60E-008 (2,6431997 %)
	CPE Alpha 1	0,8910702	0,0020454 (0,2295490 %)

RelaxIS 3.0.20.16 - Report

Datasource: LYB4.5C1.5_70C.txt_1

Circuit: I-(R)-(P)

Type	Value
**Temperature:**	70,000000
**Free variable:**	N/A
**DC Voltage:**	N/A
**AC Voltage:**	N/A
**Time:**	N/A
**Harmonic:**	N/A
**Free Variable 2:**	N/A
**Area:**	N/A
**Thickness:**	N/A

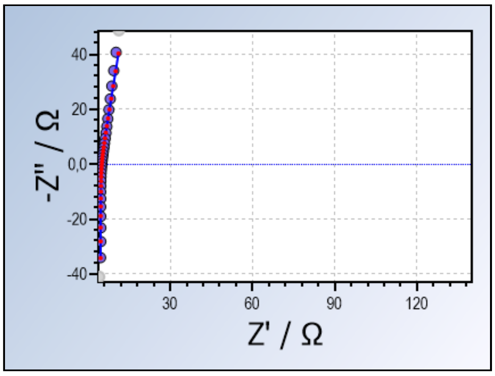

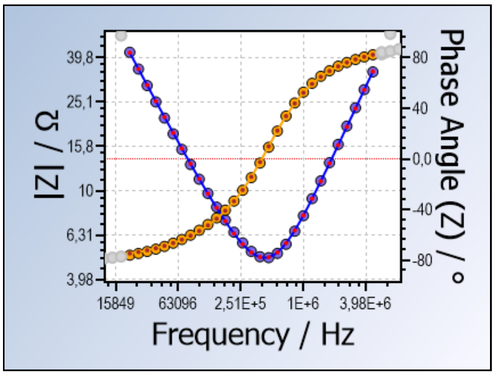

Fit Parameters:

Fix?	Name	Value	Error (Relative)
	Inductance 1	1,18E-006	3,33E-009 (0,2817373 %)
	Resistance 1	4,5292814	0,0200943 (0,4436532 %)
	CPE Q 1	6,04E-007	1,48E-008 (2,4571413 %)
	CPE Alpha 1	0,8975345	0,0018904 (0,2106231 %)

RelaxIS 3.0.20.16 - Report

Datasource: LYB4.5C1.5_80C.txt_1

Circuit: I-(R)-(P)

Type	Value
**Temperature:**	80,000000
**Free variable:**	N/A
**DC Voltage:**	N/A
**AC Voltage:**	N/A
**Time:**	N/A
**Harmonic:**	N/A
**Free Variable 2:**	N/A
**Area:**	N/A
**Thickness:**	N/A

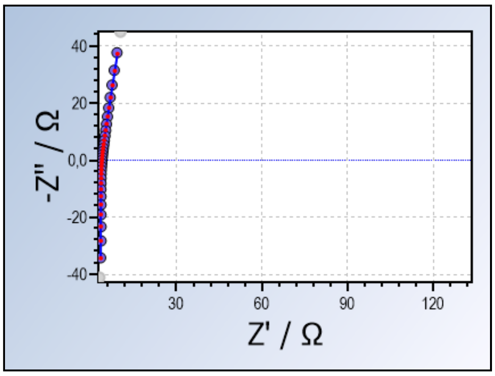

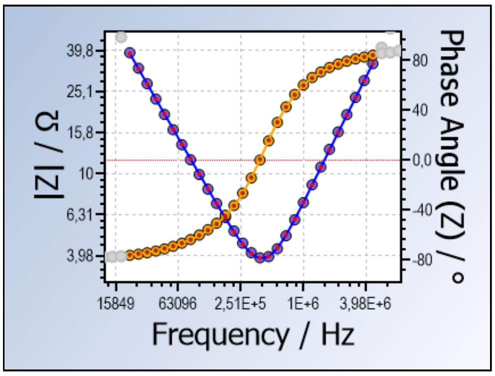

Fit Parameters:

Fix?	Name	Value	Error (Relative)
	Inductance 1	1,19E-006	2,21E-009 (0,1864575 %)
	Resistance 1	3,4395019	0,0120103 (0,3491877 %)
	CPE Q 1	6,30E-007	1,05E-008 (1,6698968 %)
	CPE Alpha 1	0,9004481	0,0012790 (0,1420381 %)

RelaxIS 3.0.20.16 - Report

Datasource: LYB4.5C1.5_90C.txt_1

Circuit: I-(R)-(P)

Type	Value
**Temperature:**	90,000000
**Free variable:**	N/A
**DC Voltage:**	N/A
**AC Voltage:**	N/A
**Time:**	N/A
**Harmonic:**	N/A
**Free Variable 2:**	N/A
**Area:**	N/A
**Thickness:**	N/A

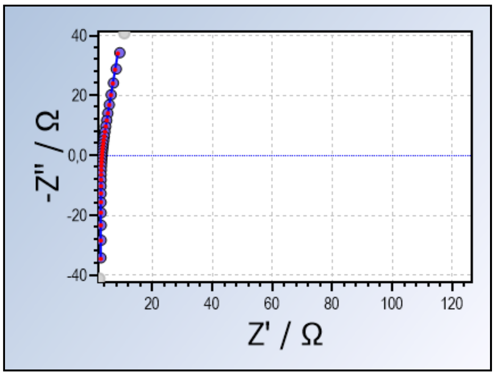

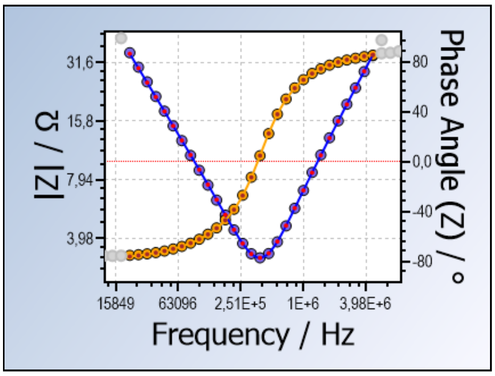

Fit Parameters:

Fix?	Name	Value	Error (Relative)
	Inductance 1	1,19E-006	1,75E-009 (0,1462819 %)
	Resistance 1	2,7231706	0,0087910 (0,3228235 %)
	CPE Q 1	7,46E-007	1,01E-008 (1,3525201 %)
	CPE Alpha 1	0,8939833	0,0010336 (0,1156199 %)
