# Supplementary material for: Investigation of Structure, Ionic Conductivity, and Electrochemical Stability of Halogen Substitution in Solid-State Ion Conductor Li3YBrxCl6–x
Source: J Phys Chem C Nanomater Interfaces. 2022 Dec 16;127(1):125–32. doi: 10.1021/acs.jpcc.2c07910 (PMC9841563; doi:10.1021/acs.jpcc.2c07910)
Supplement: Supplementary file 6 — jp2c07910_si_006.pdf [file jp2c07910_si_006.pdf]

RelaxIS 3.0.20.16 - Report

Datasource: LYB1.5C4.5_30C.txt_1

Circuit: I-(R)-P

Type	Value
**Temperature:**	30,000000
**Free variable:**	N/A
**DC Voltage:**	N/A
**AC Voltage:**	N/A
**Time:**	N/A
**Harmonic:**	N/A
**Free Variable 2:**	N/A
**Area:**	N/A
**Thickness:**	N/A

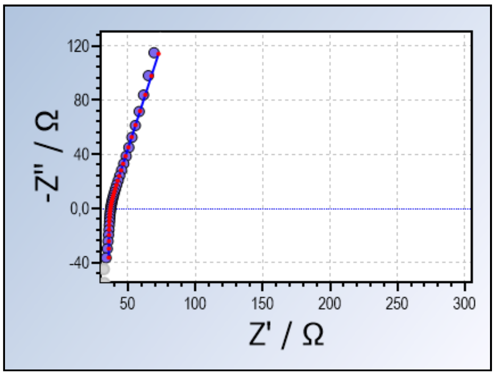

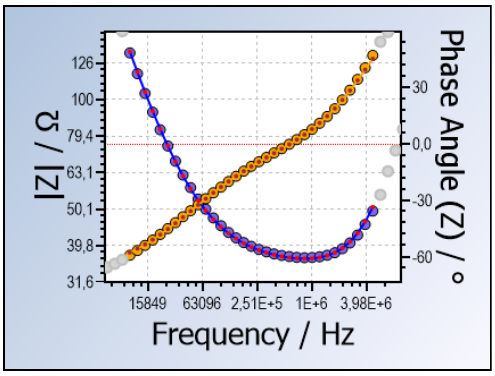

Fit Parameters:

Fix?	Name	Value	Error (Relative)
	Inductance 1	1,27E-006	7,48E-009 (0,5908283 %)
	Resistance 1	35,463290	0,0865826 (0,2441471 %)
	CPE Q 1	1,21E-006	3,27E-008 (2,7022050 %)
	CPE Alpha 1	0,7997882	0,0022555 (0,2820067 %)

RelaxIS 3.0.20.16 - Report

Datasource: LYB1.5C4.5_40C.txt_1

Circuit: I-(R)-P

Type	Value
**Temperature:**	40,000000
**Free variable:**	N/A
**DC Voltage:**	N/A
**AC Voltage:**	N/A
**Time:**	N/A
**Harmonic:**	N/A
**Free Variable 2:**	N/A
**Area:**	N/A
**Thickness:**	N/A

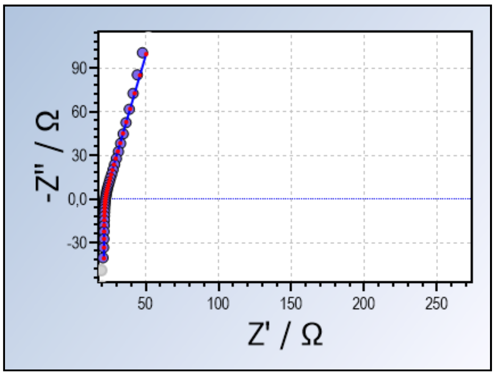

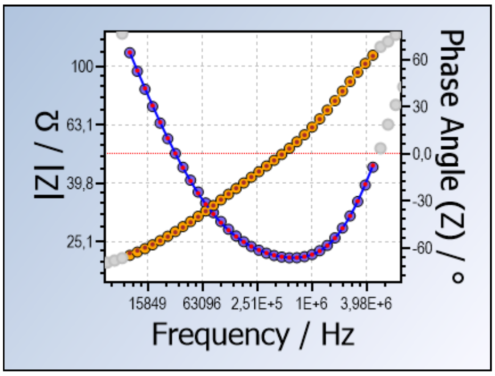

Fit Parameters:

Fix?	Name	Value	Error (Relative)
	Inductance 1	1,42E-006	4,33E-009 (0,3060129 %)
	Resistance 1	21,083925	0,0418776 (0,1986232 %)
	CPE Q 1	1,13E-006	1,99E-008 (1,7597247 %)
	CPE Alpha 1	0,8190321	0,0014524 (0,1773325 %)

RelaxIS 3.0.20.16 - Report

Datasource: LYB1.5C4.5_50C.txt_1

Circuit: I-(R)-P

Type	Value
**Temperature:**	50,000000
**Free variable:**	N/A

**DC Voltage:**	N/A
**AC Voltage:**	N/A
**Time:**	N/A
**Harmonic:**	N/A
**Free Variable 2:**	N/A
**Area:**	N/A
**Thickness:**	N/A

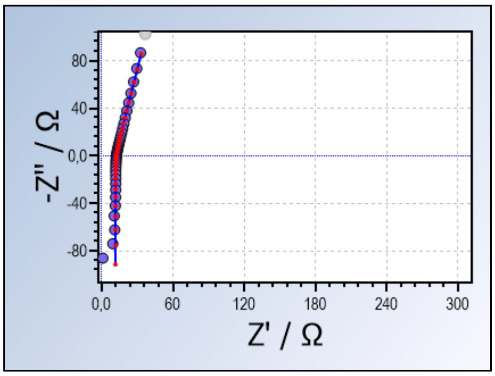

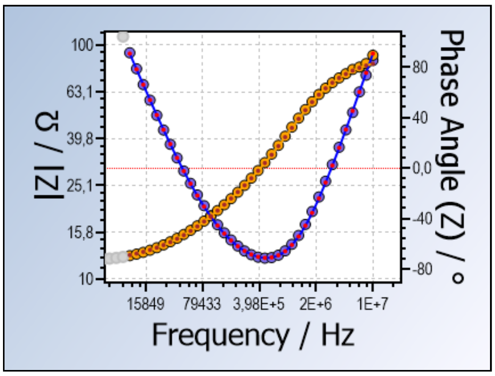

Fit Parameters:

Fix?	Name	Value	Error (Relative)
	Inductance 1	1,46E-006	8,01E-009 (0,5495647 %)
	Resistance 1	11,450243	0,0748855 (0,6540080 %)
	CPE Q 1	1,02E-006	4,54E-008 (4,4497882 %)
	CPE Alpha 1	0,8427789	0,0036162 (0,4290810 %)

RelaxIS 3.0.20.16 - Report

Datasource: LYB1.5C4.5_60C.txt_1

Circuit: I-(R)-P

Type	Value
**Temperature:**	60,000000
**Free variable:**	N/A
**DC Voltage:**	N/A
**AC Voltage:**	N/A
**Time:**	N/A
**Harmonic:**	N/A
**Free Variable 2:**	N/A
**Area:**	N/A
**Thickness:**	N/A

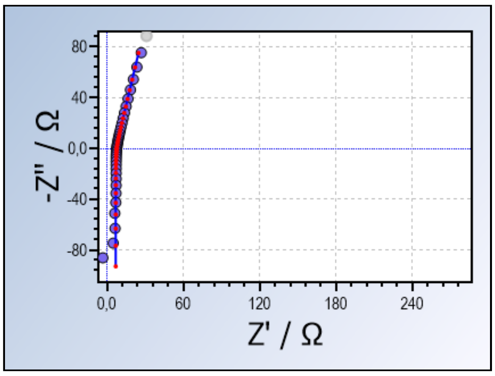

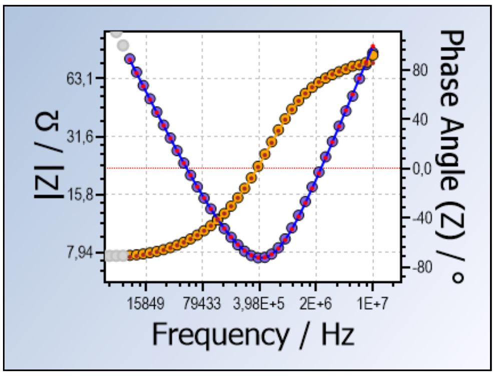

Fit Parameters:

Fix?	Name	Value	Error (Relative)
	Inductance 1	1,48E-006	7,74E-009 (0,5234711 %)
	Resistance 1	6,6672115	0,0557920 (0,8368113 %)
	CPE Q 1	1,07E-006	4,67E-008 (4,3585059 %)
	CPE Alpha 1	0,8507344	0,0034974 (0,4111078 %)

RelaxIS 3.0.20.16 - Report

Datasource: LYB1.5C4.5_70C.txt_1

Circuit: I-(R)-P

Type	Value
**Temperature:**	70,000000
**Free variable:**	N/A
**DC Voltage:**	N/A
**AC Voltage:**	N/A
**Time:**	N/A
**Harmonic:**	N/A
**Free Variable 2:**	N/A
**Area:**	N/A
**Thickness:**	N/A

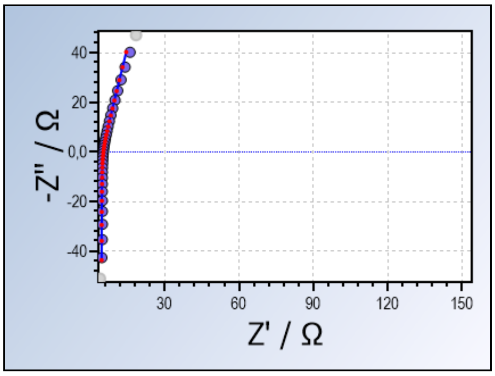

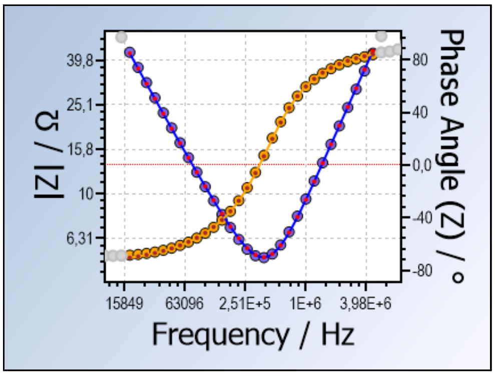

Fit Parameters:

Fix?	Name	Value	Error (Relative)
	Inductance 1	1,51E-006	4,59E-009 (0,3041415 %)
	Resistance 1	4,4078074	0,0254718 (0,5778785 %)
	CPE Q 1	1,30E-006	3,64E-008 (2,8078321 %)
	CPE Alpha 1	0,8452851	0,0021941 (0,2595731 %)

RelaxIS 3.0.20.16 - Report

Datasource: LYB1.5C4.5_80C.txt_1

Circuit: I-(R)-P

Type	Value
**Temperature:**	80,000000
**Free variable:**	N/A
**DC Voltage:**	N/A
**AC Voltage:**	N/A
**Time:**	N/A
**Harmonic:**	N/A
**Free Variable 2:**	N/A
**Area:**	N/A
**Thickness:**	N/A

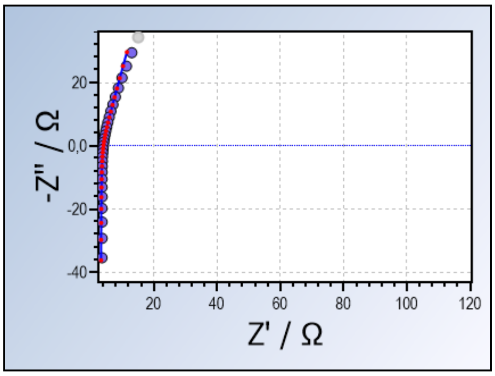

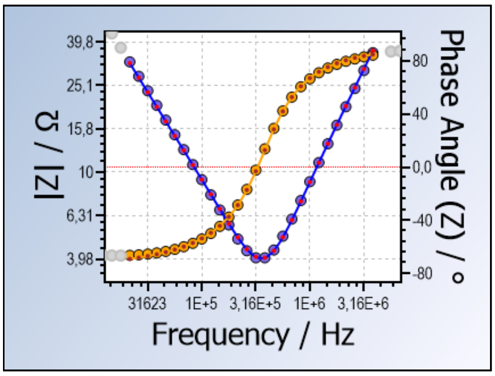

Fit Parameters:

Fix?	Name	Value	Error (Relative)
	Inductance 1	1,52E-006	6,31E-009 (0,4155928 %)
	Resistance 1	3,1736750	0,0318479 (1,0035023 %)
	CPE Q 1	1,82E-006	7,59E-008 (4,1651030 %)
	CPE Alpha 1	0,8277693	0,0032313 (0,3903618 %)

RelaxIS 3.0.20.16 - Report

Datasource: LYB1.5C4.5_90C.txt_1

Circuit: I-(R)-P

Type	Value
**Temperature:**	90,000000
**Free variable:**	N/A
**DC Voltage:**	N/A
**AC Voltage:**	N/A
**Time:**	N/A
**Harmonic:**	N/A
**Free Variable 2:**	N/A
**Area:**	N/A
**Thickness:**	N/A

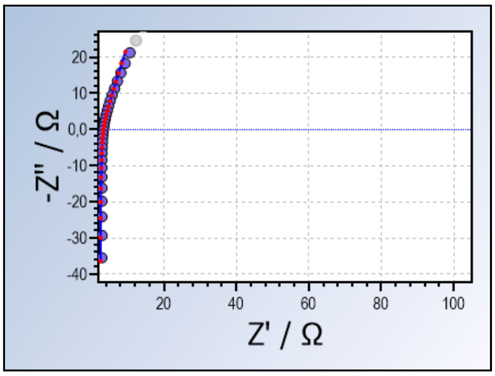

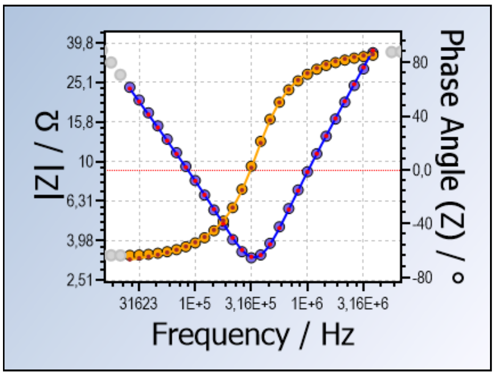

Fit Parameters:

Fix?	Name	Value	Error (Relative)
	Inductance 1	1,53E-006	7,40E-009 (0,4848501 %)
	Resistance 1	2,3496628	0,0367644 (1,5646679 %)
	CPE Q 1	2,87E-006	1,59E-007 (5,5321677 %)
	CPE Alpha 1	0,8023499	0,0042744 (0,5327297 %)

RelaxIS 3.0.20.16 - Report

Datasource: LYB1.5C4.5_0C.txt_1

Circuit: I-(R)(P)-P

Type	Value
**Temperature:**	0,0
**Free variable:**	N/A
**DC Voltage:**	N/A
**AC Voltage:**	N/A
**Time:**	47,007415
**Harmonic:**	N/A
**Free Variable 2:**	N/A
**Area:**	N/A
**Thickness:**	N/A

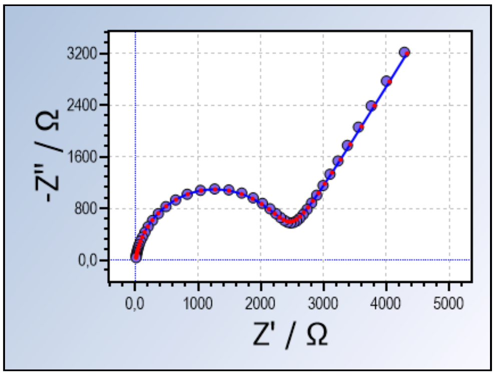

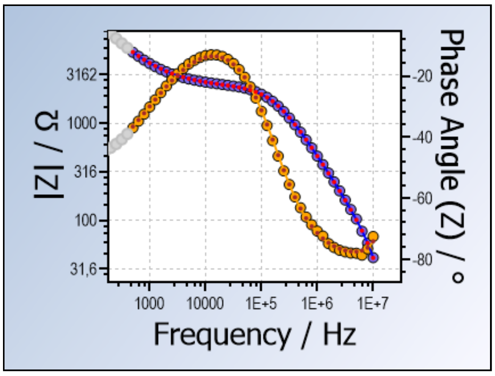

Fit Parameters:

Fix?	Name	Value	Error (Relative)
	Inductance 1	3,06E-007	7,95E-009 (2,5958189 %)
	Resistance 1	2301,7206	11,015249 (0,4785659 %)
	CPE Q 1	1,19E-009	3,26E-011 (2,7410749 %)
	CPE Alpha 1	0,9224873	0,0018059 (0,1957634 %)
	CPE Q 2	1,53E-006	3,38E-008 (2,2057197 %)
	CPE Alpha 2	0,6395470	0,0029525 (0,4616611 %)

RelaxIS 3.0.20.16 - Report

RelaxIS 3 Impedance Spectrum Analysis

Datasource: LYB1.5C4.5_-25C.txt_1

Circuit: I-(R)(P)-P

Type	Value
**Temperature:**	-25,00000
**Free variable:**	N/A
**DC Voltage:**	N/A
**AC Voltage:**	N/A
**Time:**	47,690989
**Harmonic:**	N/A
**Free Variable 2:**	N/A
**Area:**	N/A
**Thickness:**	N/A

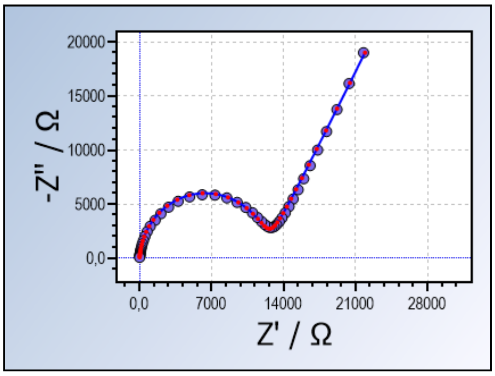

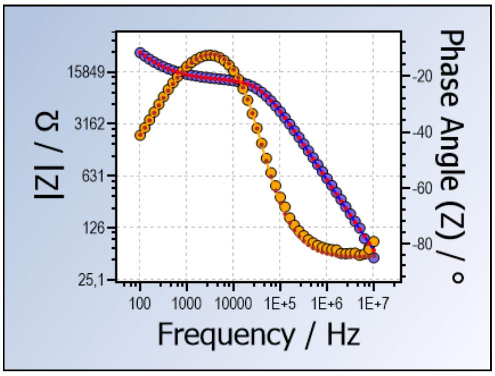

Fit Parameters:

Fix?	Name	Value	Error (Relative)
X	Inductance 1	0,0	0,0 (NaN %)
	Resistance 1	1,20E+004	197,58784 (1,6491932 %)
	CPE Q 1	4,81E-010	3,63E-011 (7,5496888 %)
	CPE Alpha 1	0,9707864	0,0053823 (0,5544302 %)
	CPE Q 2	5,38E-007	6,32E-008 (11,762574 %)
	CPE Alpha 2	0,6928205	0,0168895 (2,4377888 %)

RelaxIS 3.0.20.16 - Report

RelaxIS 3 Impedance Spectrum Analysis

Datasource: LYB1.5C4.5_-50C.txt_1

Circuit: I-(R)(P)-P

Type	Value
**Temperature:**	-50,00000
**Free variable:**	N/A
**DC Voltage:**	N/A

**AC Voltage:**	N/A
**Time:**	48,782965
**Harmonic:**	N/A
**Free Variable 2:**	N/A
**Area:**	N/A
**Thickness:**	N/A

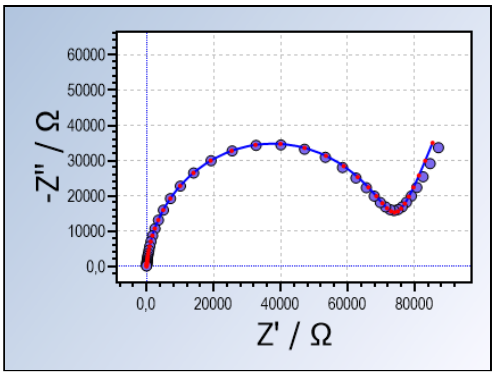

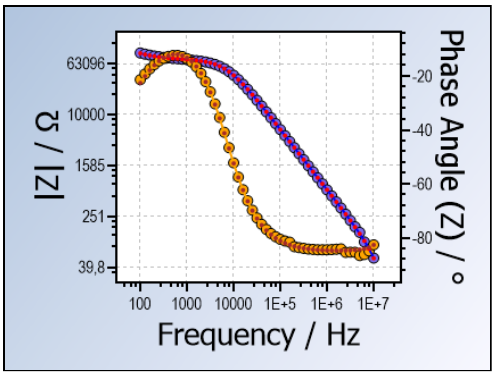

Fit Parameters:

Fix?	Name	Value	Error (Relative)
	Inductance 1	3,21E-007	1,01E-008 (3,1351607 %)
	Resistance 1	7,17E+004	392,18311 (0,5466330 %)
	CPE Q 1	5,04E-010	9,79E-012 (1,9434444 %)
	CPE Alpha 1	0,9561095	0,0016156 (0,1689810 %)
	CPE Q 2	2,11E-007	1,58E-008 (7,4832355 %)
	CPE Alpha 2	0,7550419	0,0114973 (1,5227366 %)
