# Supplementary figures and images for: Investigation of Structure, Ionic Conductivity, and Electrochemical Stability of Halogen Substitution in Solid-State Ion Conductor Li3YBrxCl6–x
Source: J Phys Chem C Nanomater Interfaces. 2022 Dec 16;127(1):125–32. doi: 10.1021/acs.jpcc.2c07910 (PMC9841563; doi:10.1021/acs.jpcc.2c07910)

30C

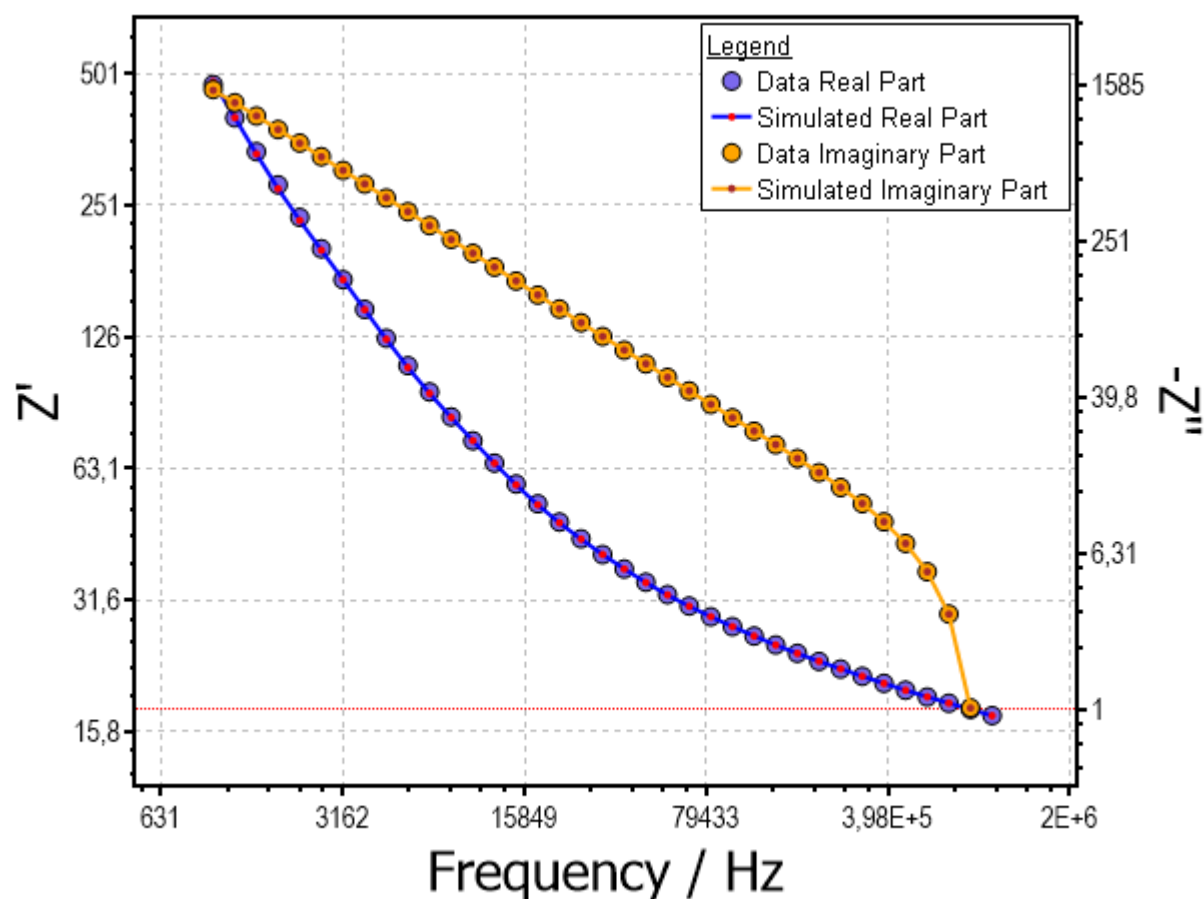

40C

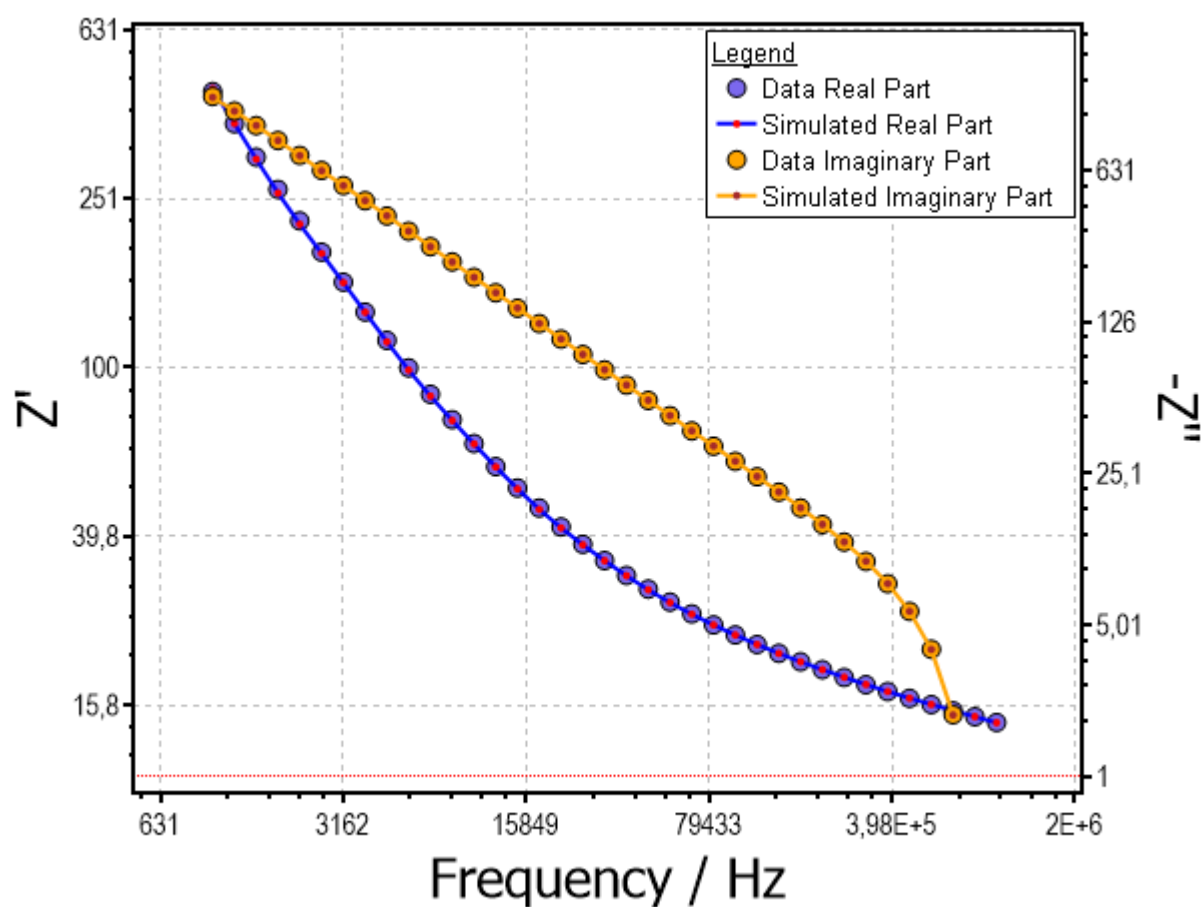

50C

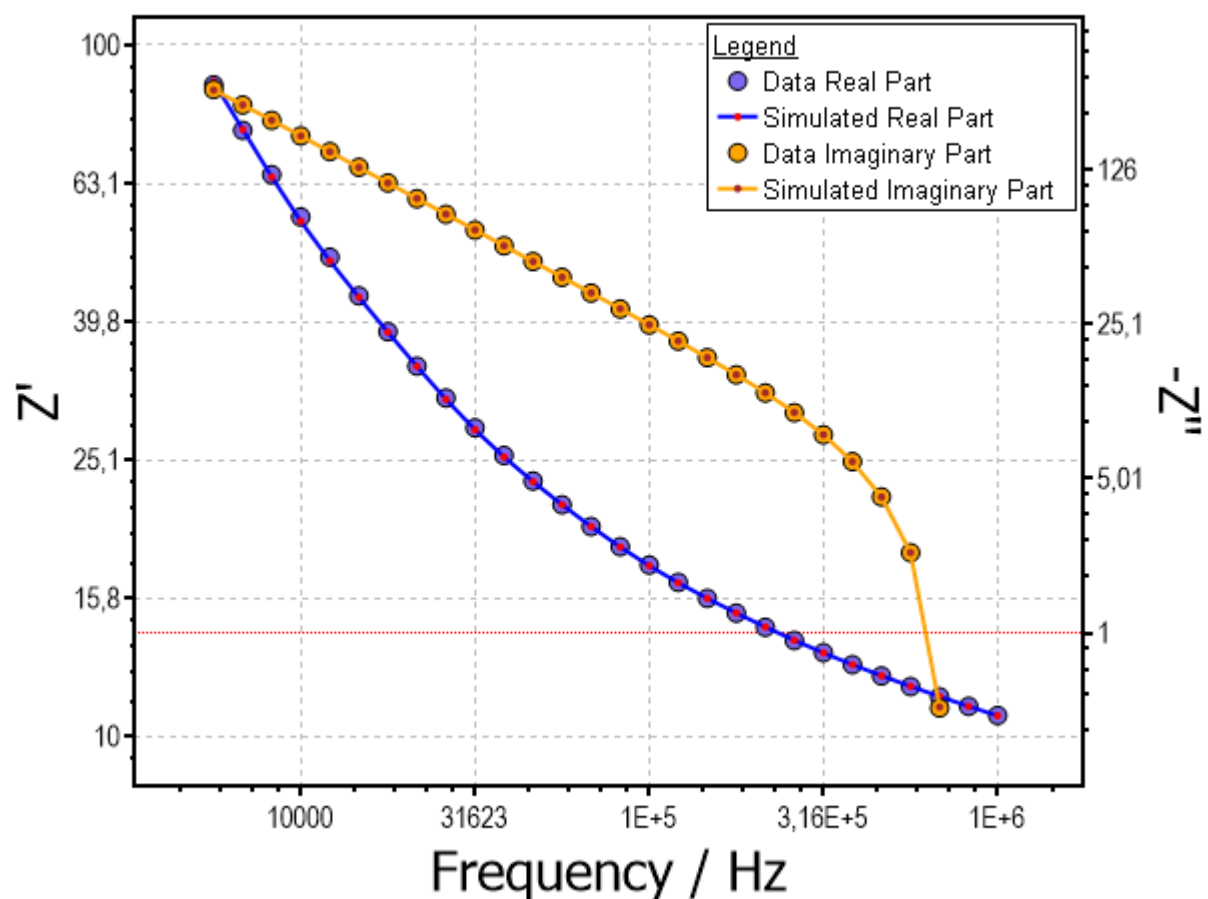

60C

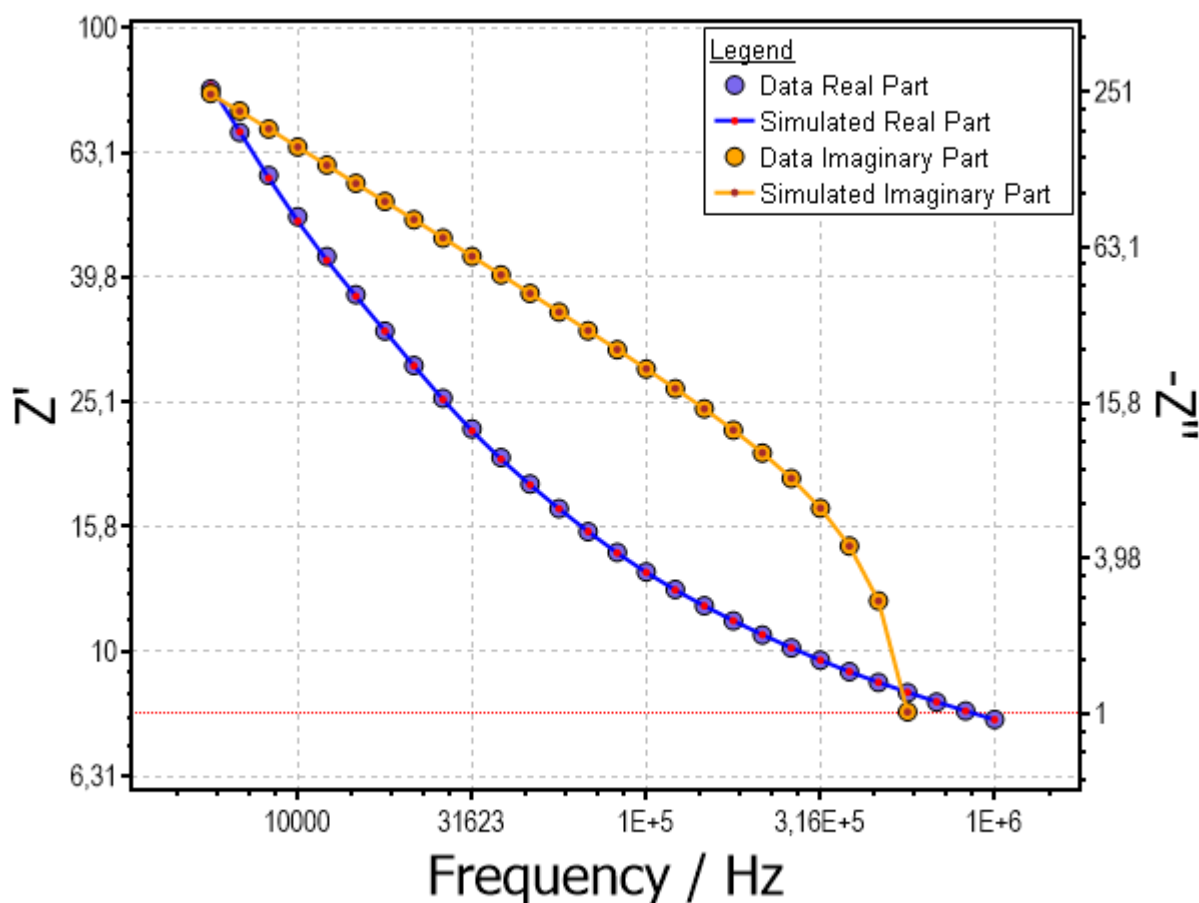

70C

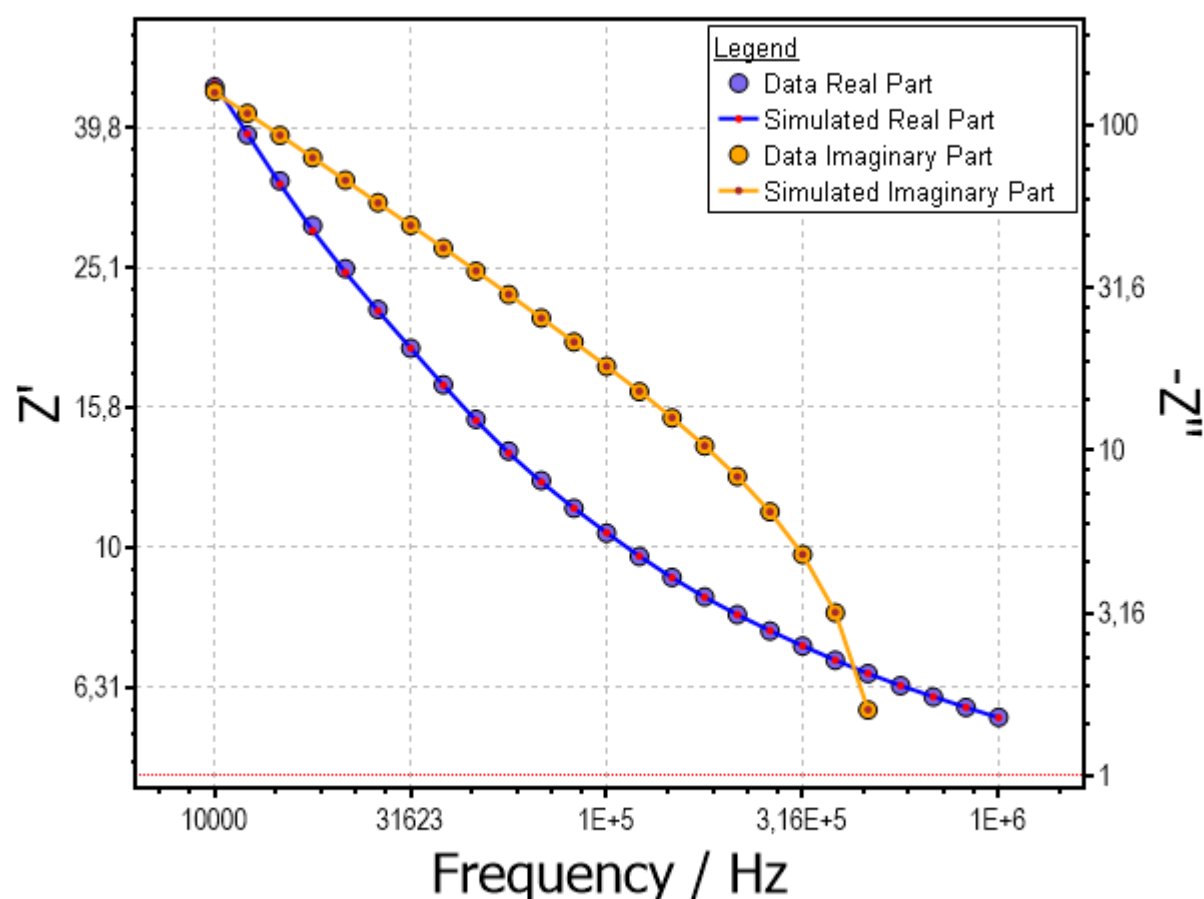

80C

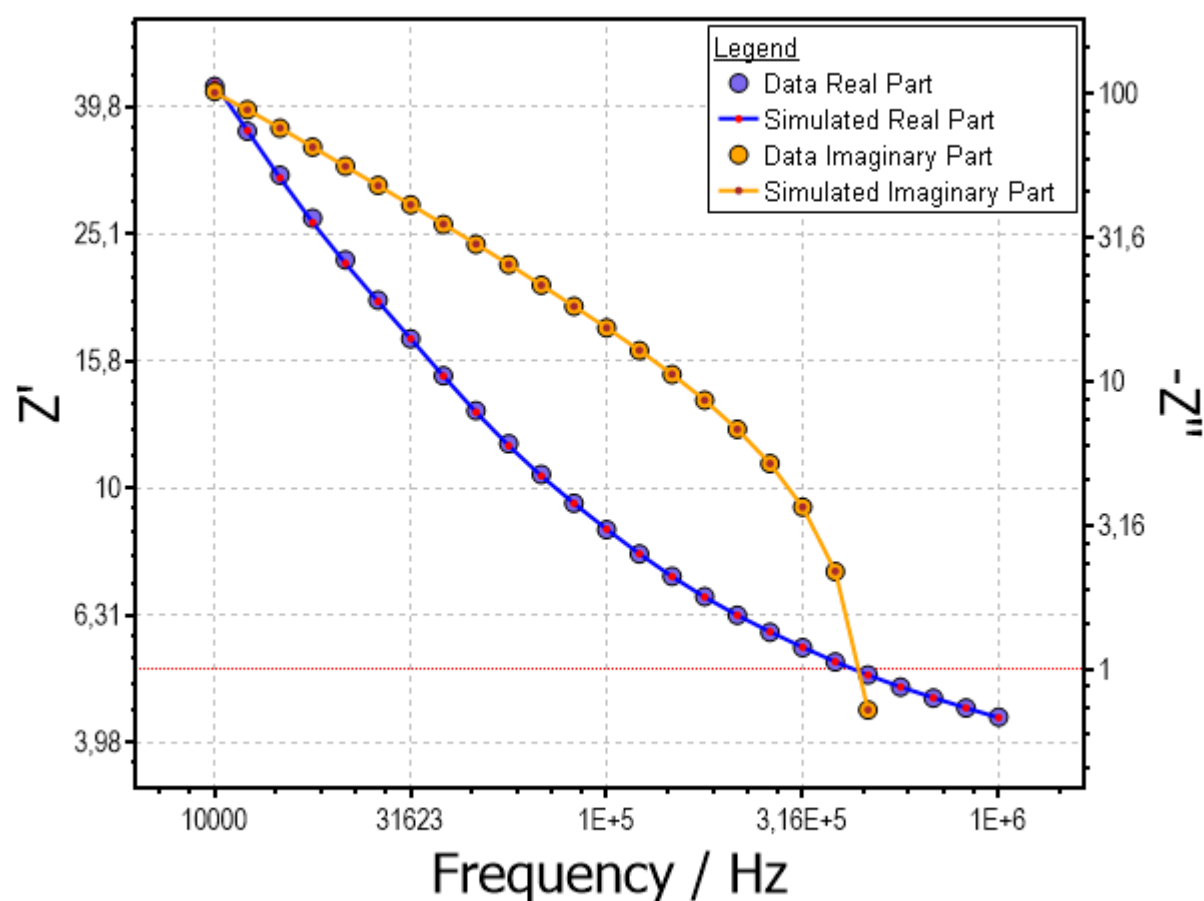

90C

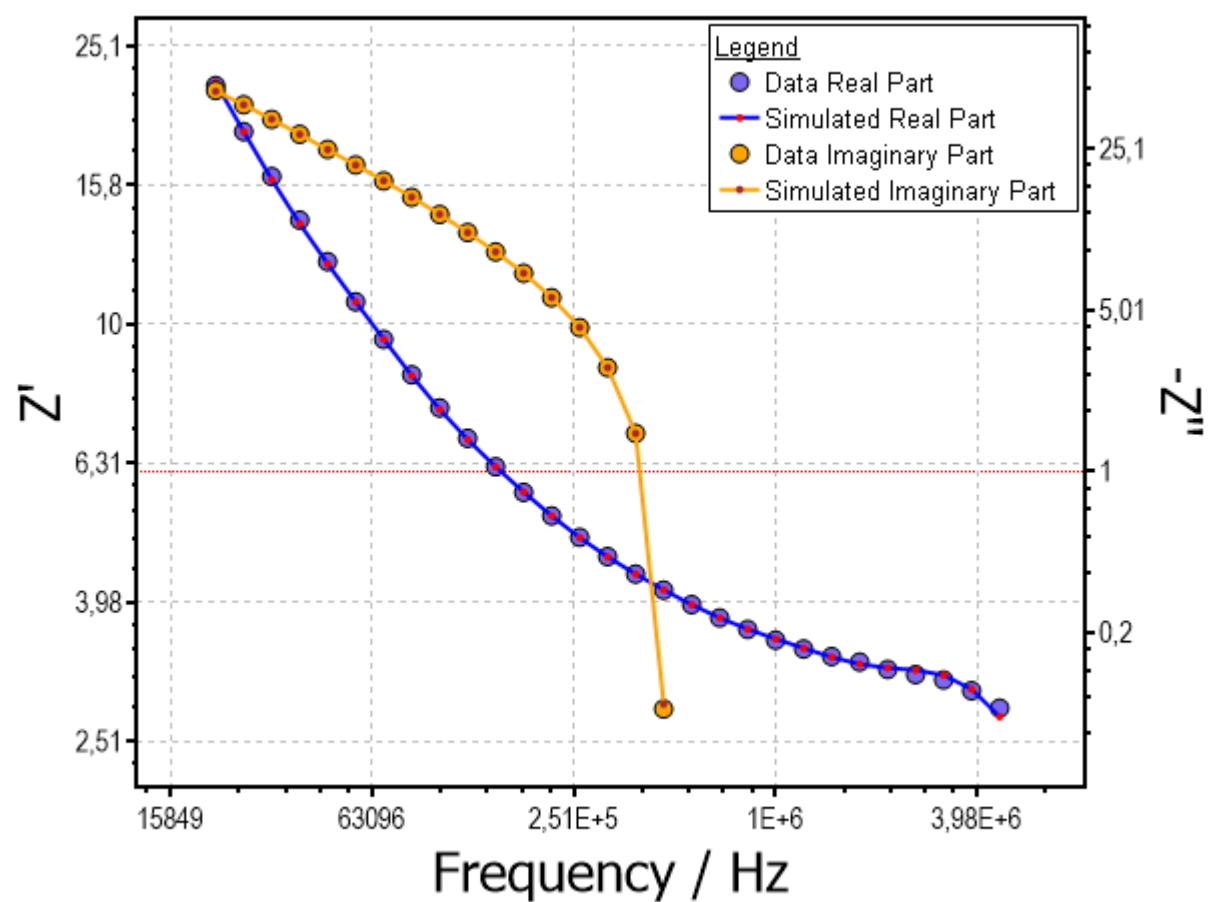

Supplement: Supplementary file 7 — jp2c07910_si_007.pdf [file jp2c07910_si_007.pdf]

30C, Li<sub>3</sub>YCl<sub>3</sub>Br<sub>3</sub>

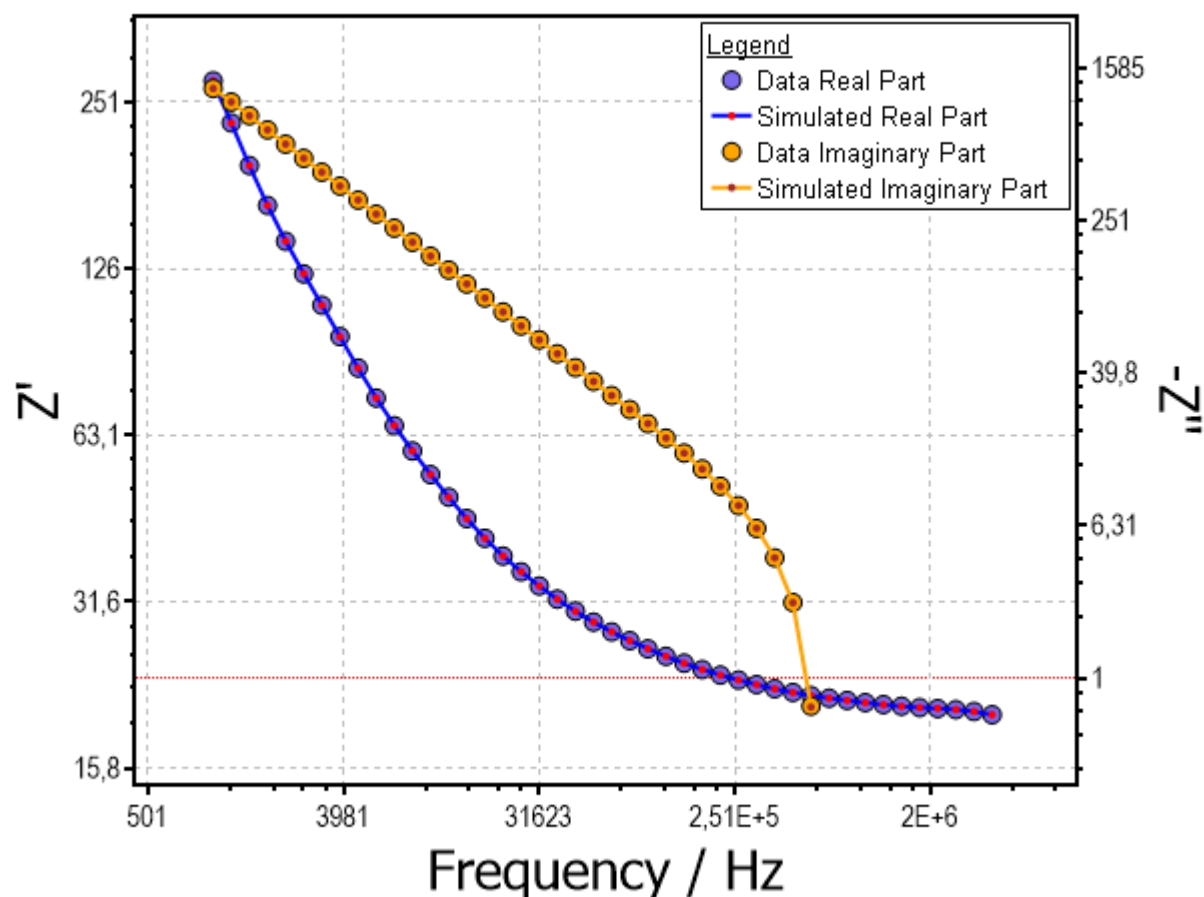

40C

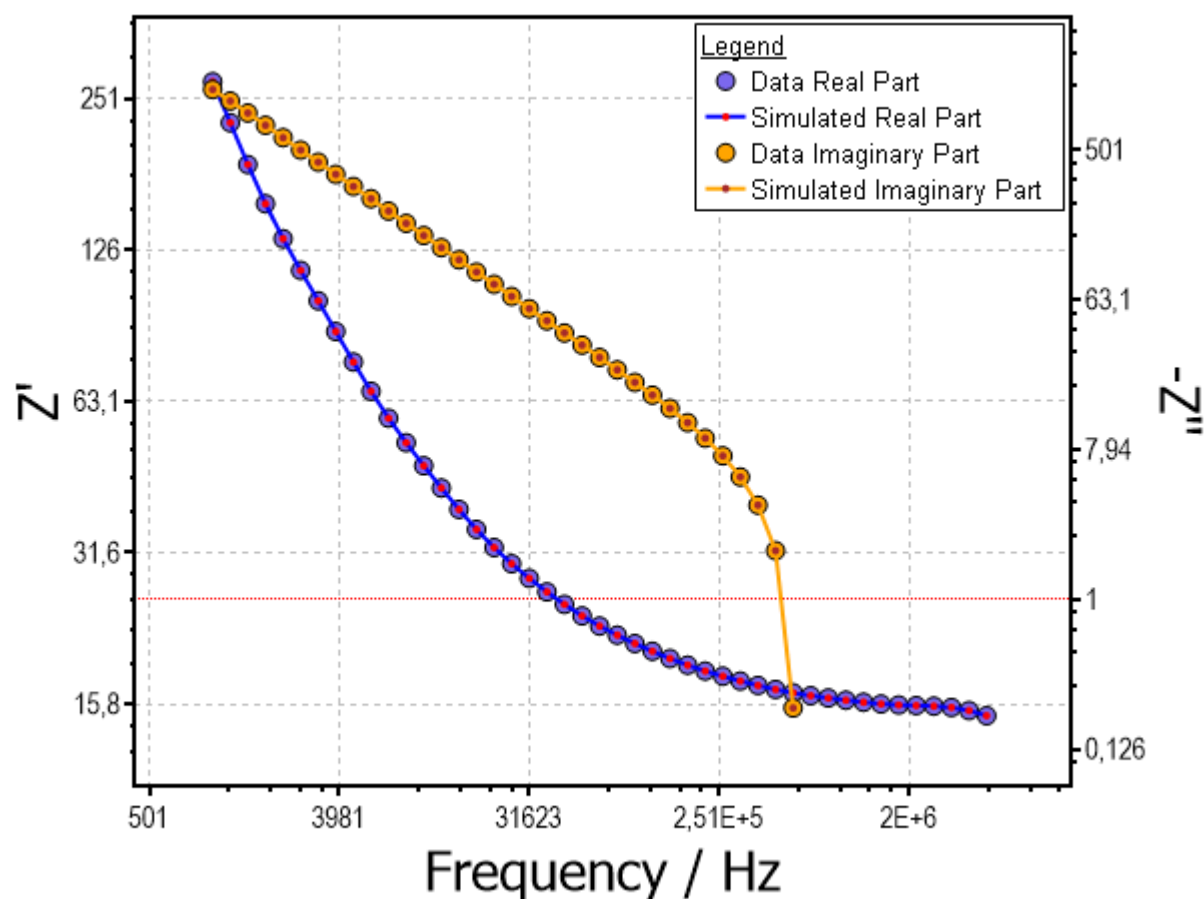

50C

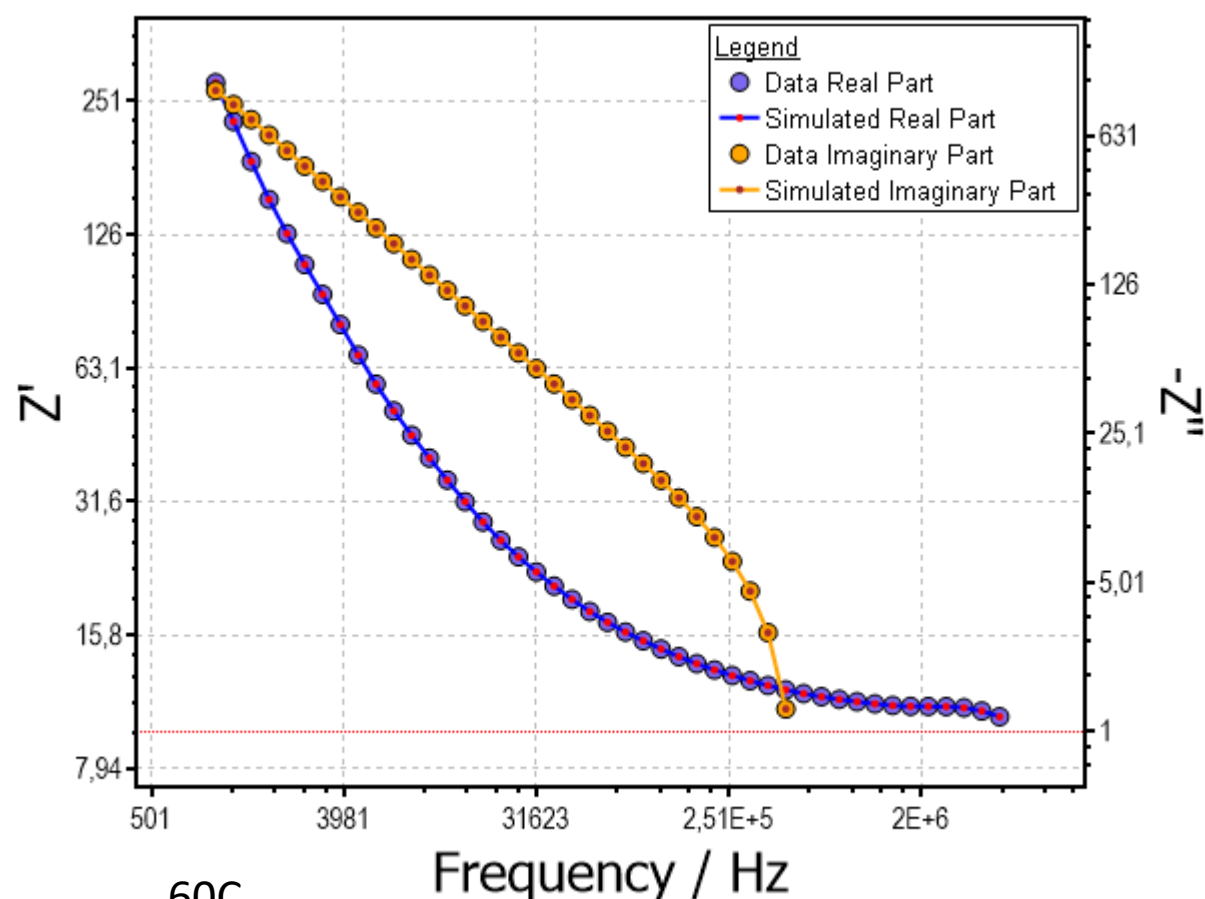

60C

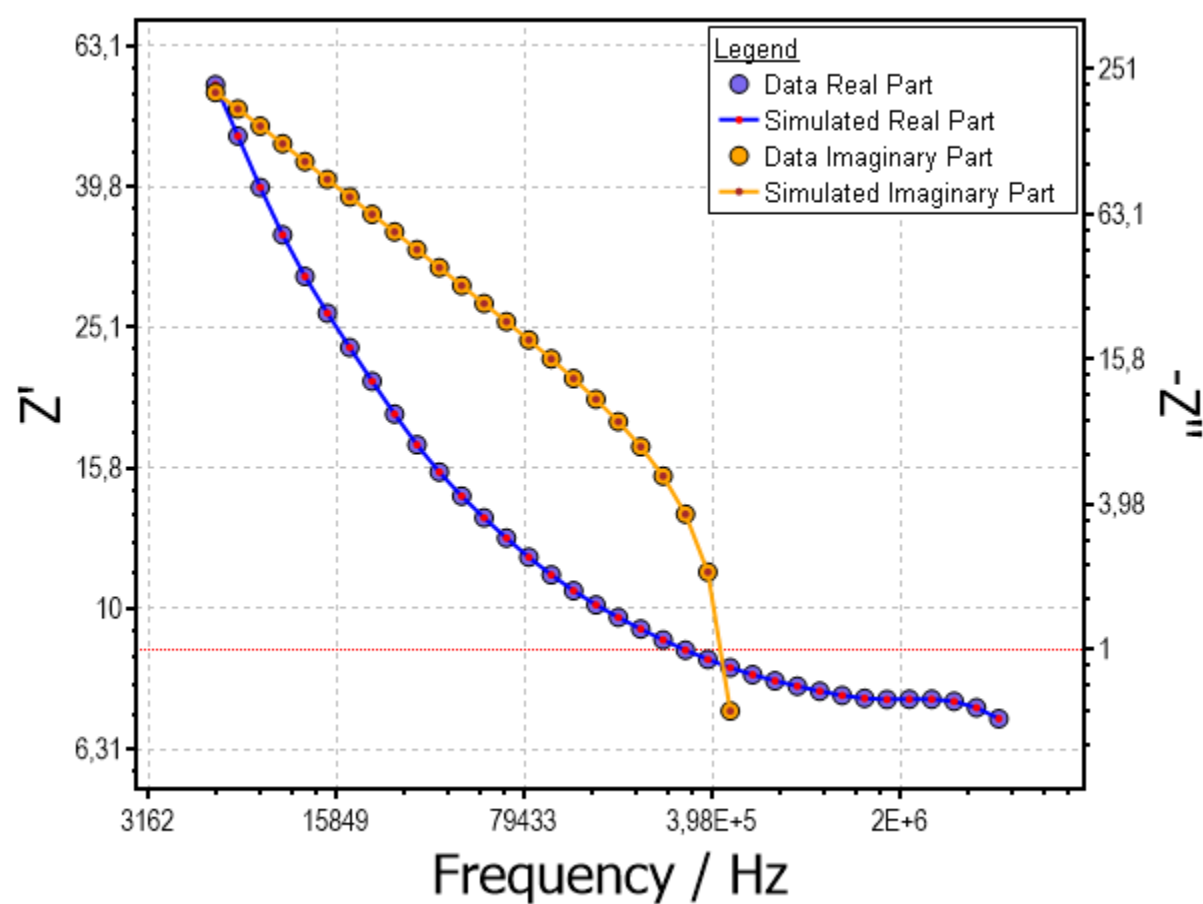

70C

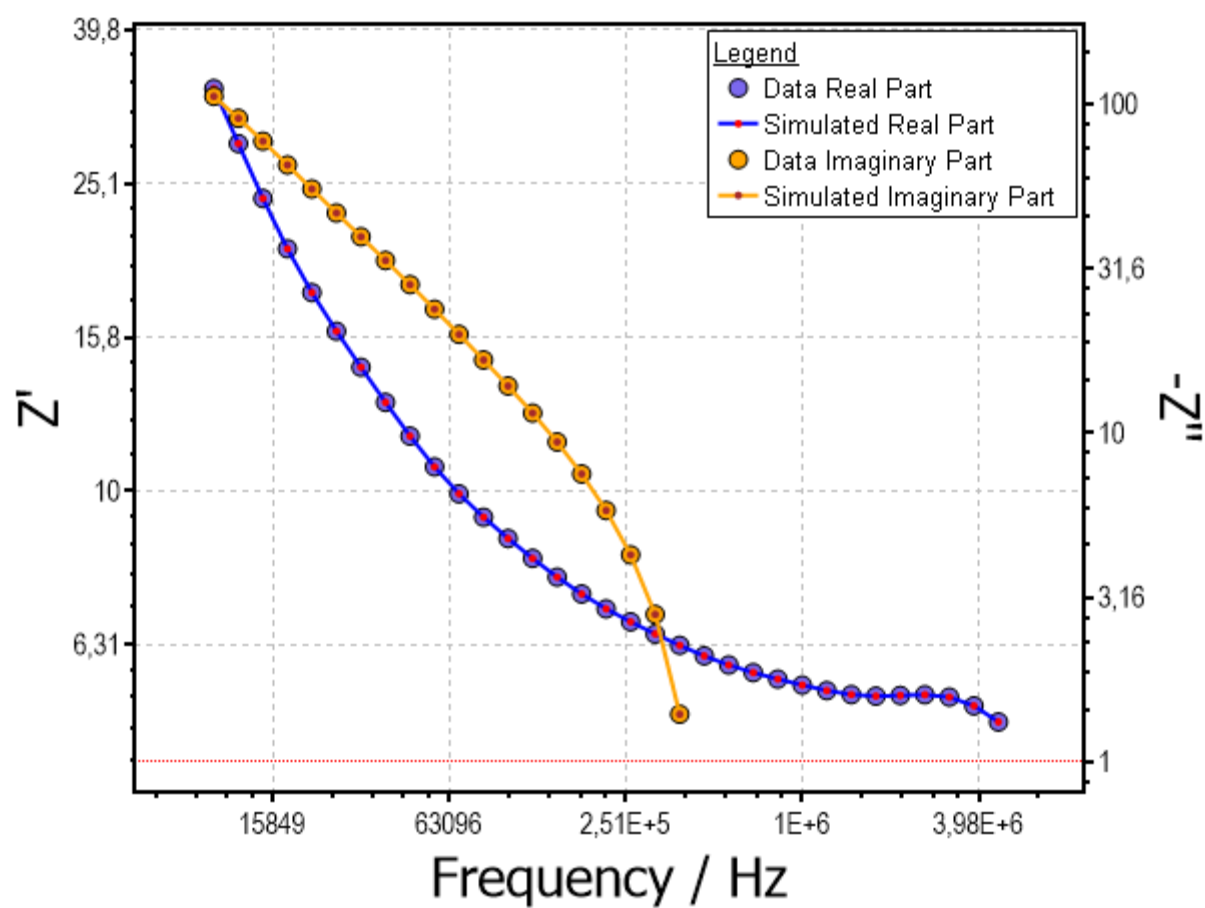

80C

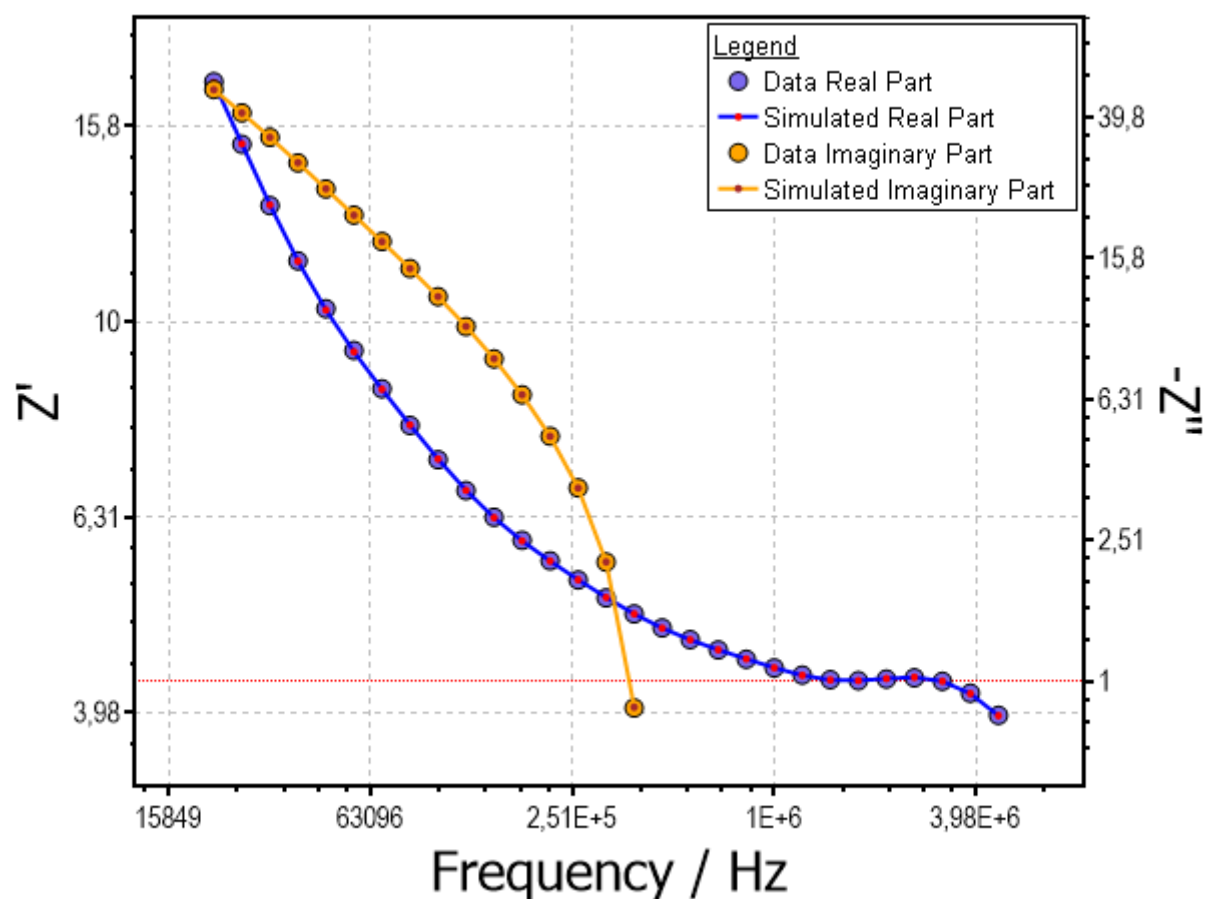

80C

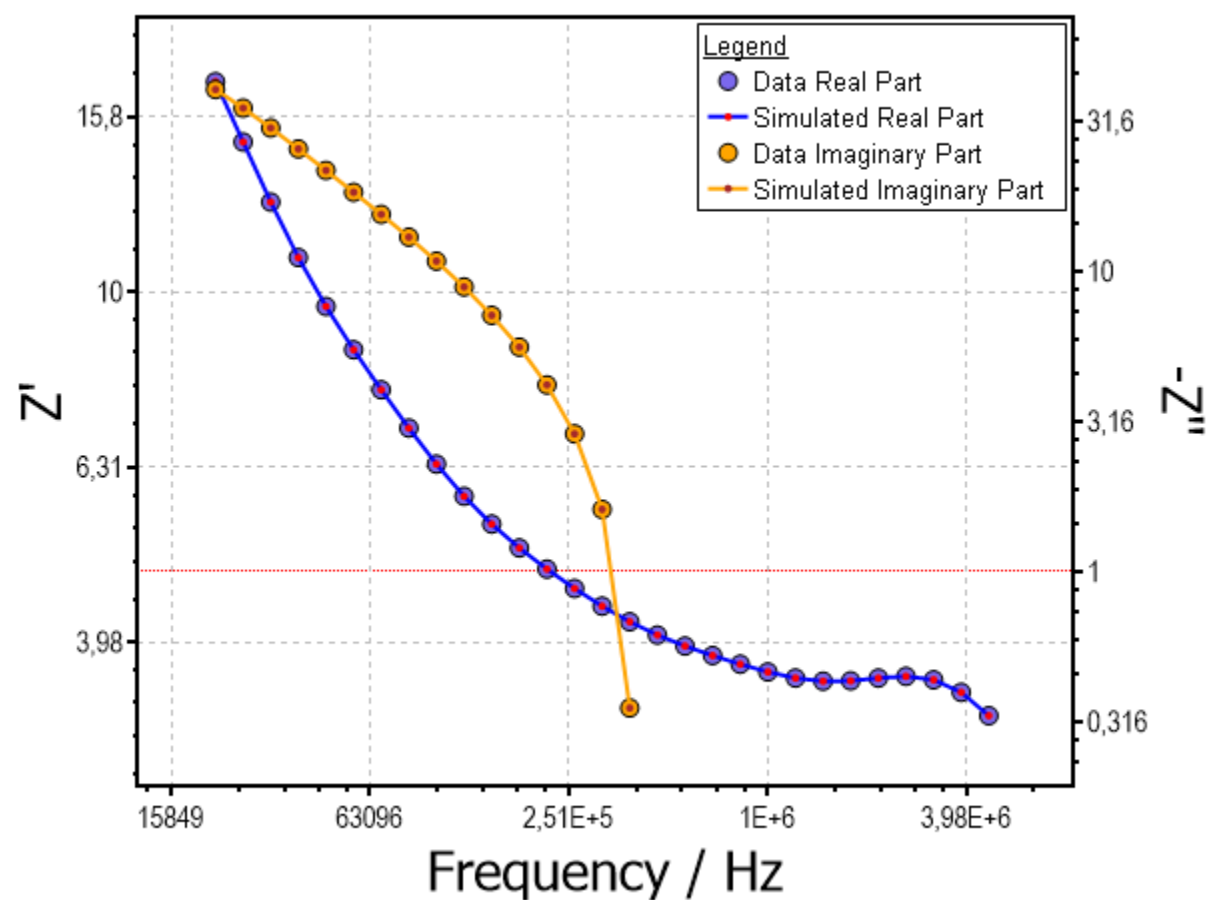

Supplement: Supplementary file 8 — jp2c07910_si_008.pdf [file jp2c07910_si_008.pdf]

30C

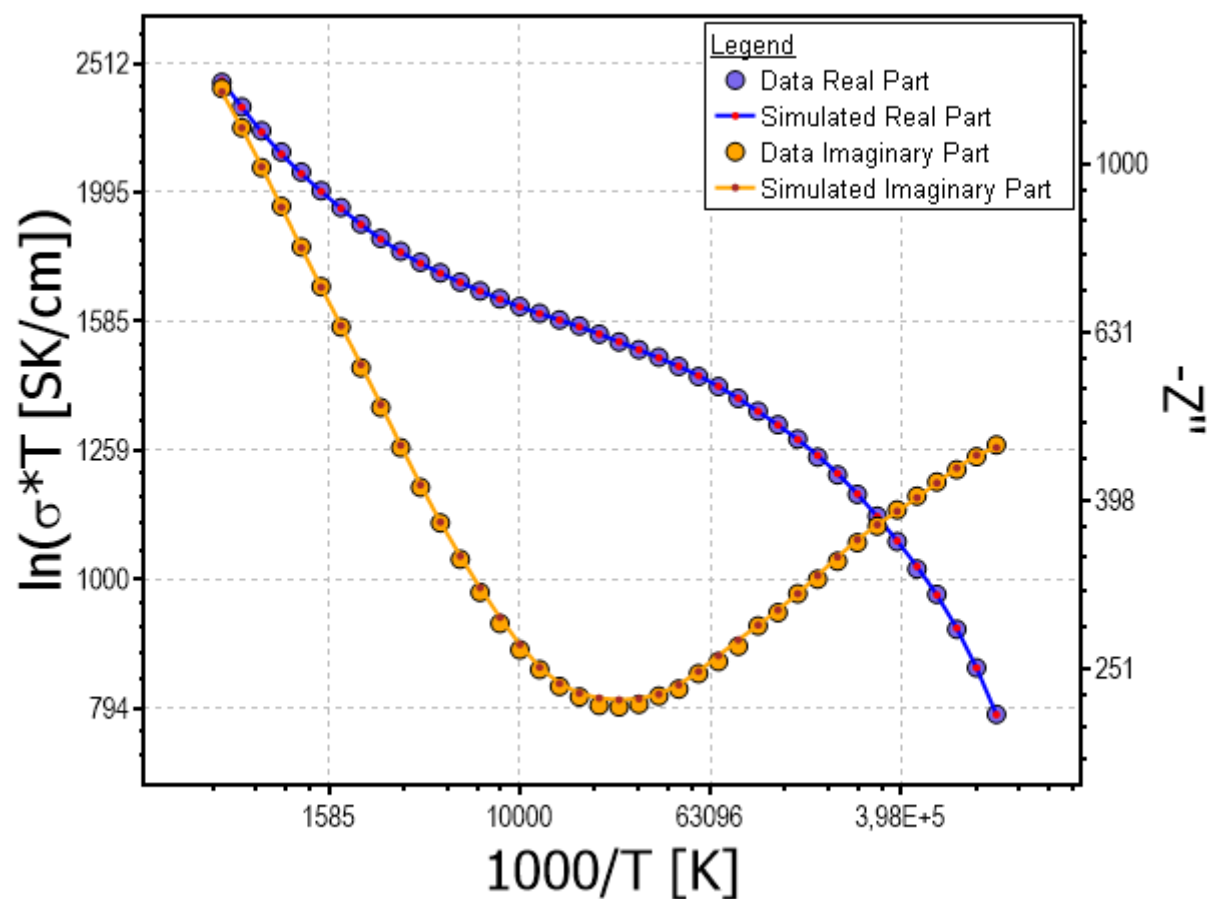

40C

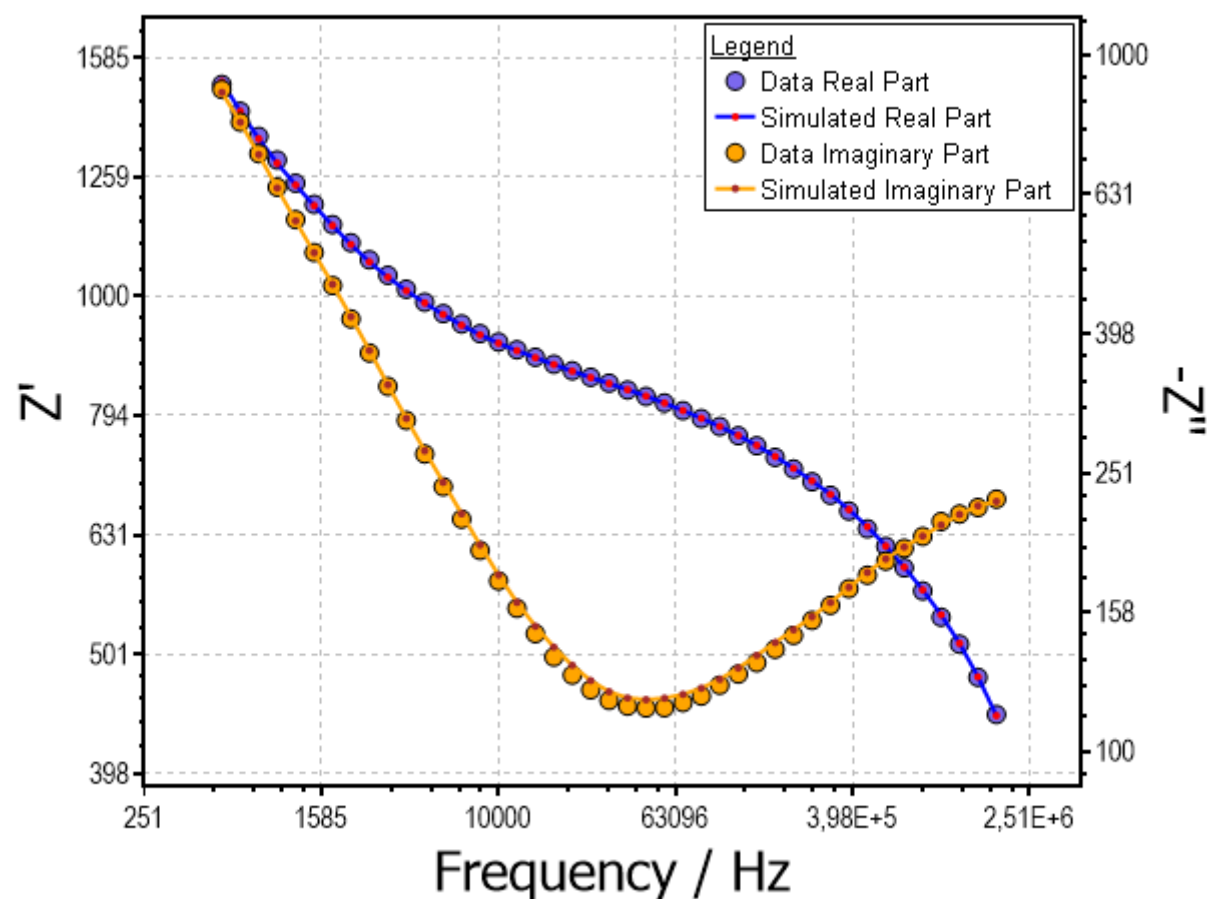

50C

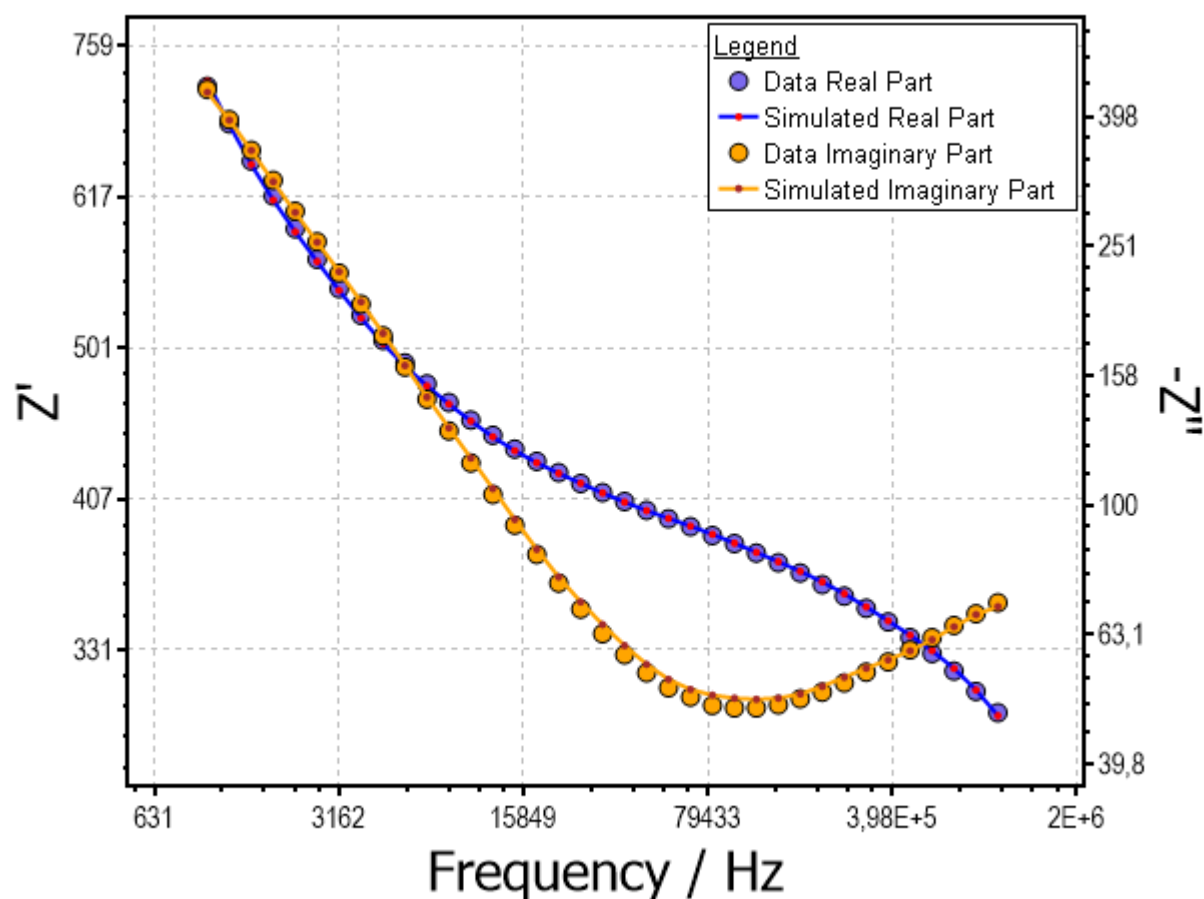

60C

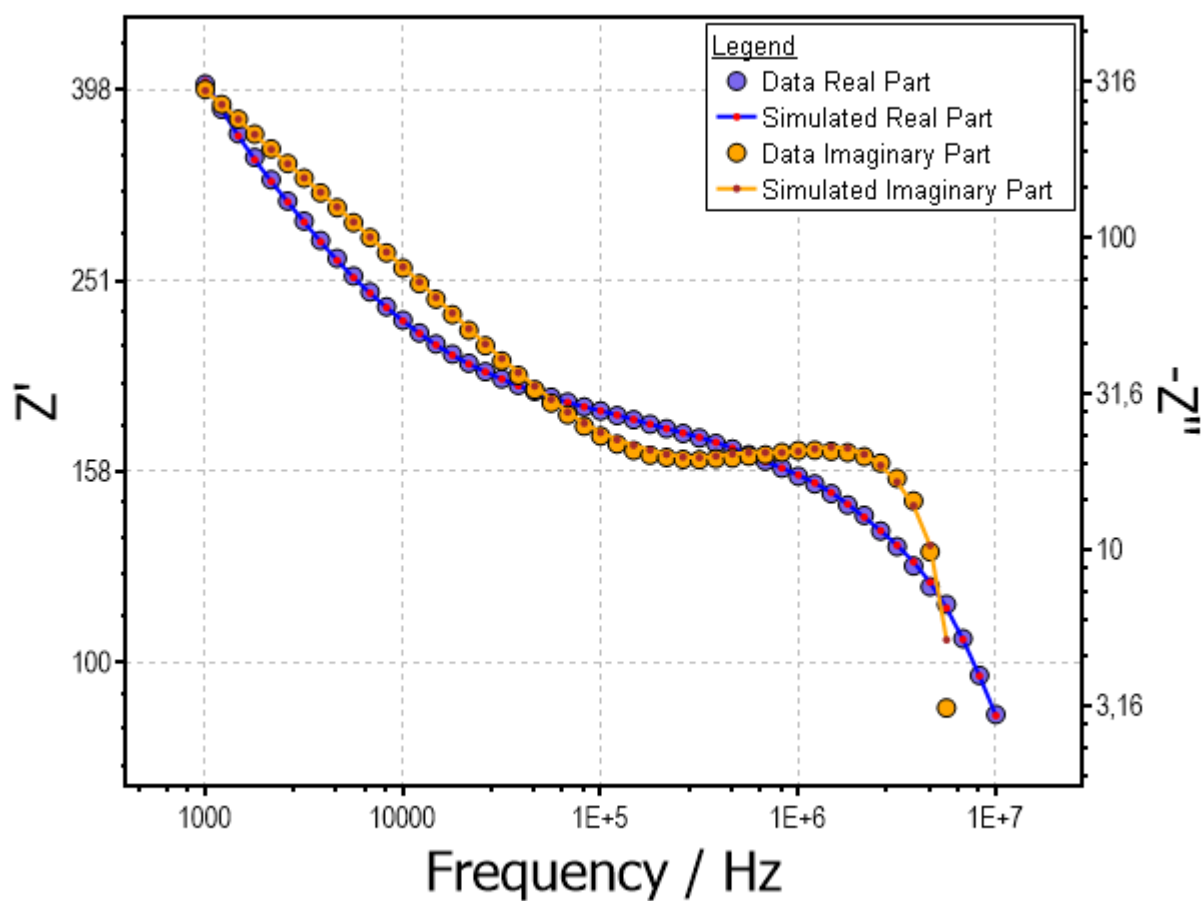

70C

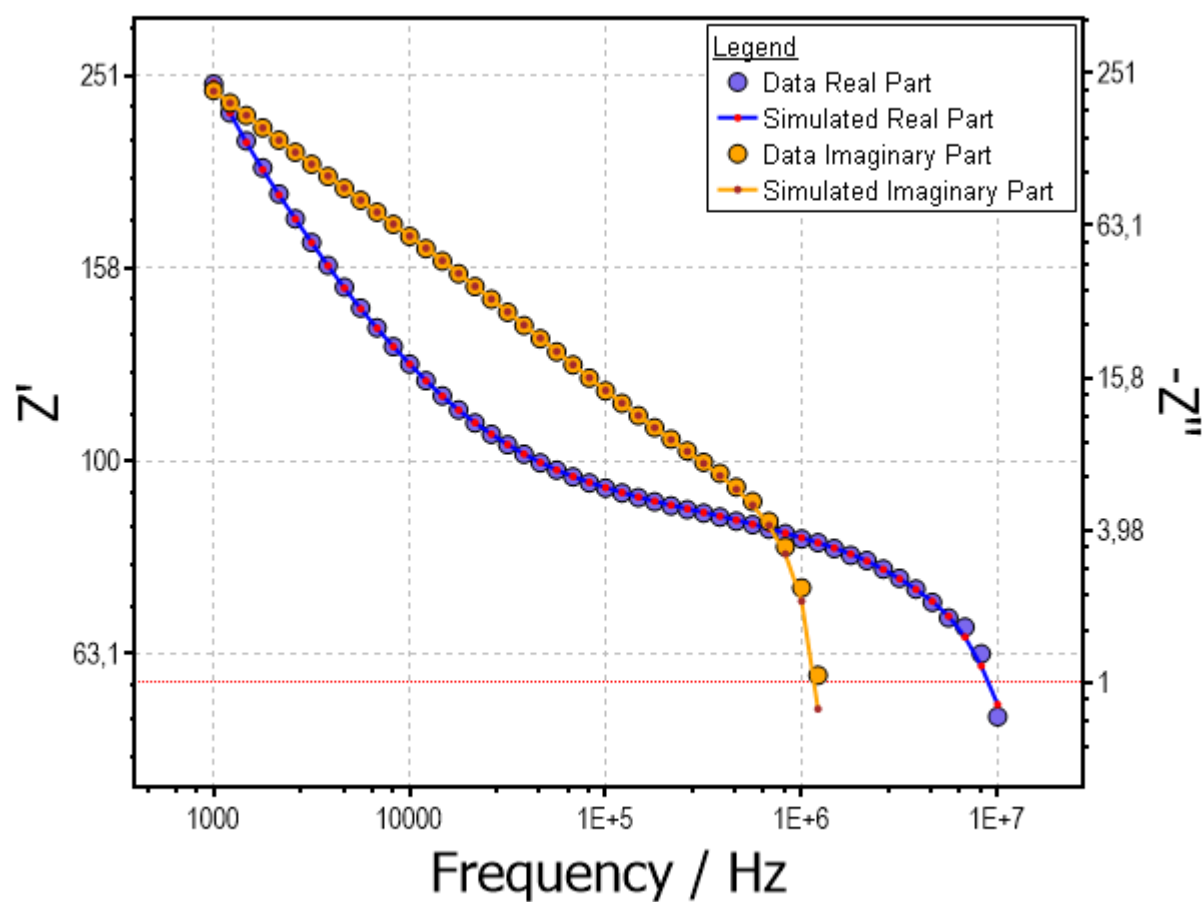

80C

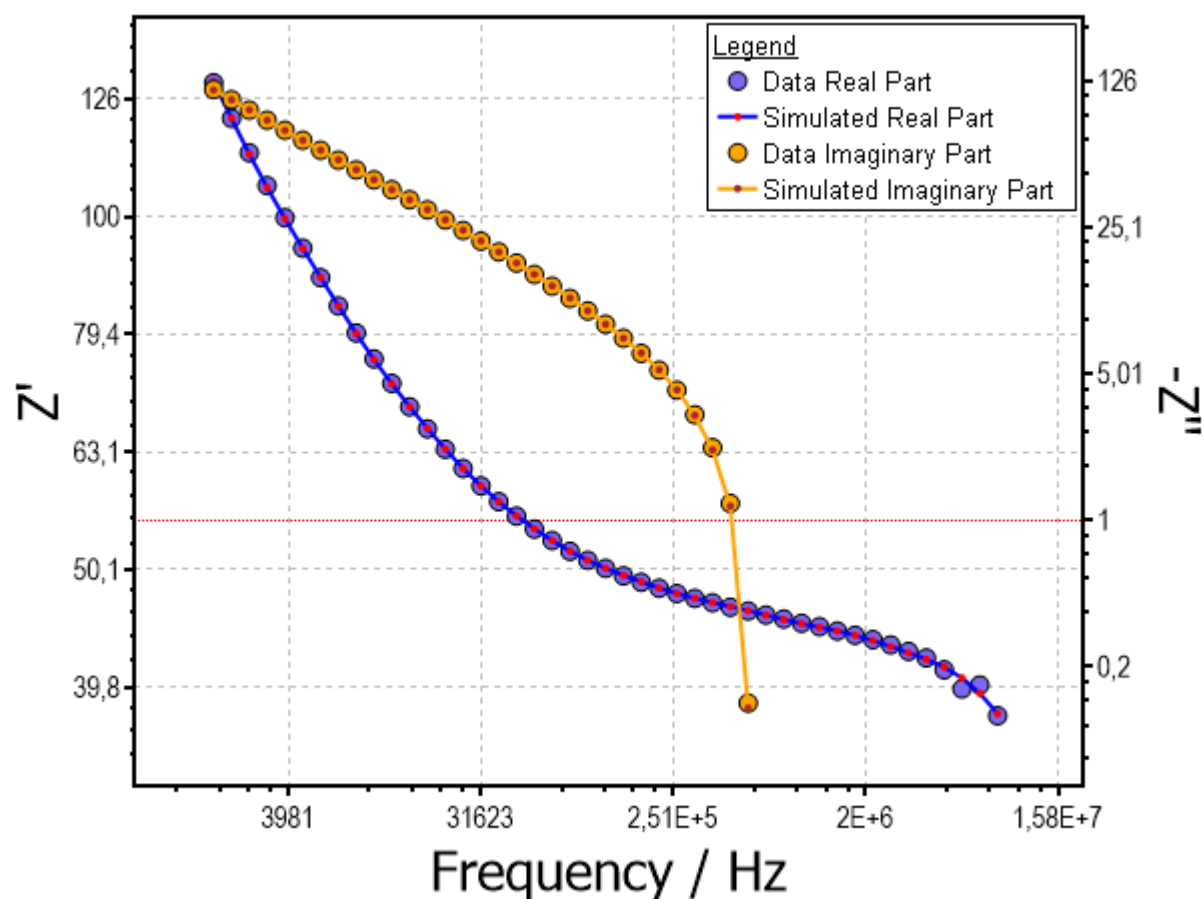

90C

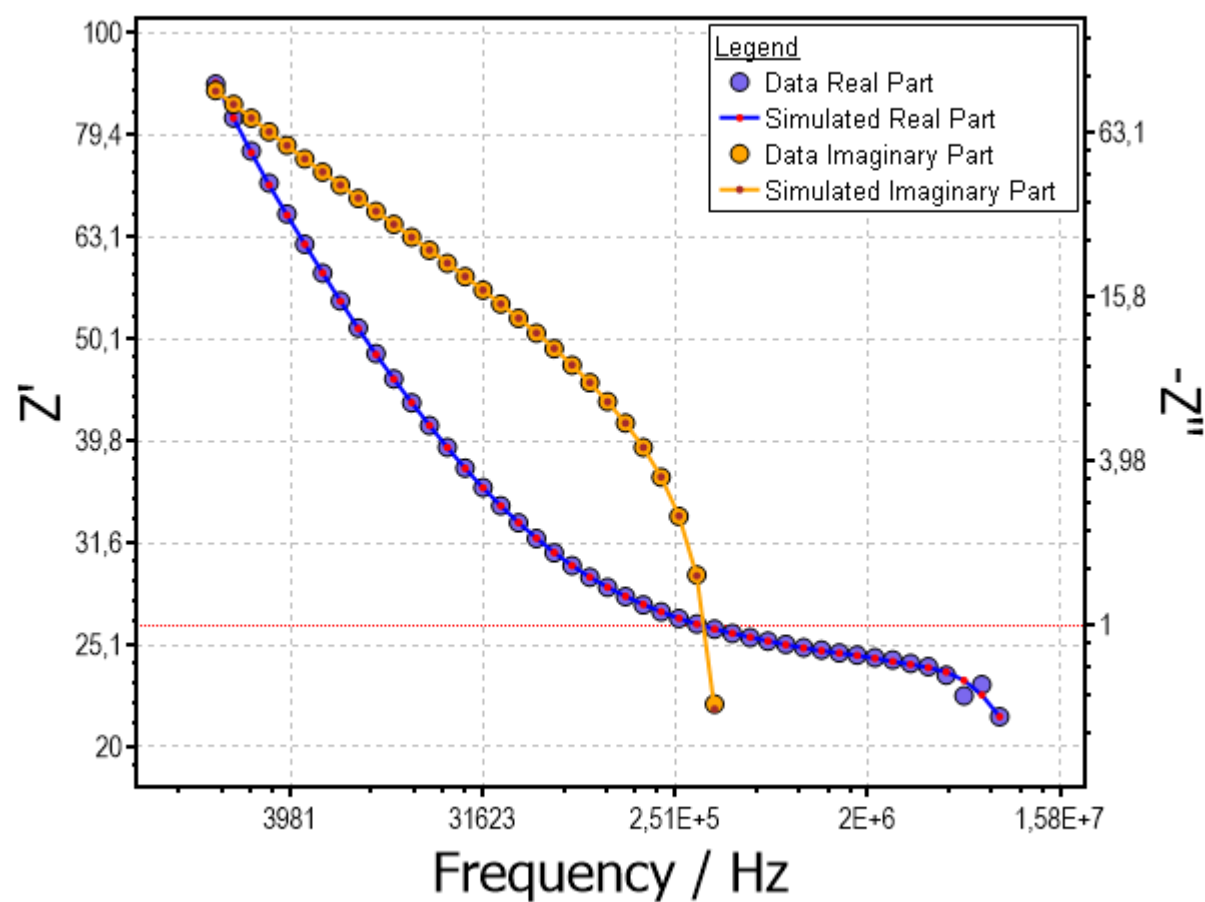

Supplement: Supplementary file 9 — jp2c07910_si_009.pdf [file jp2c07910_si_009.pdf]

30C

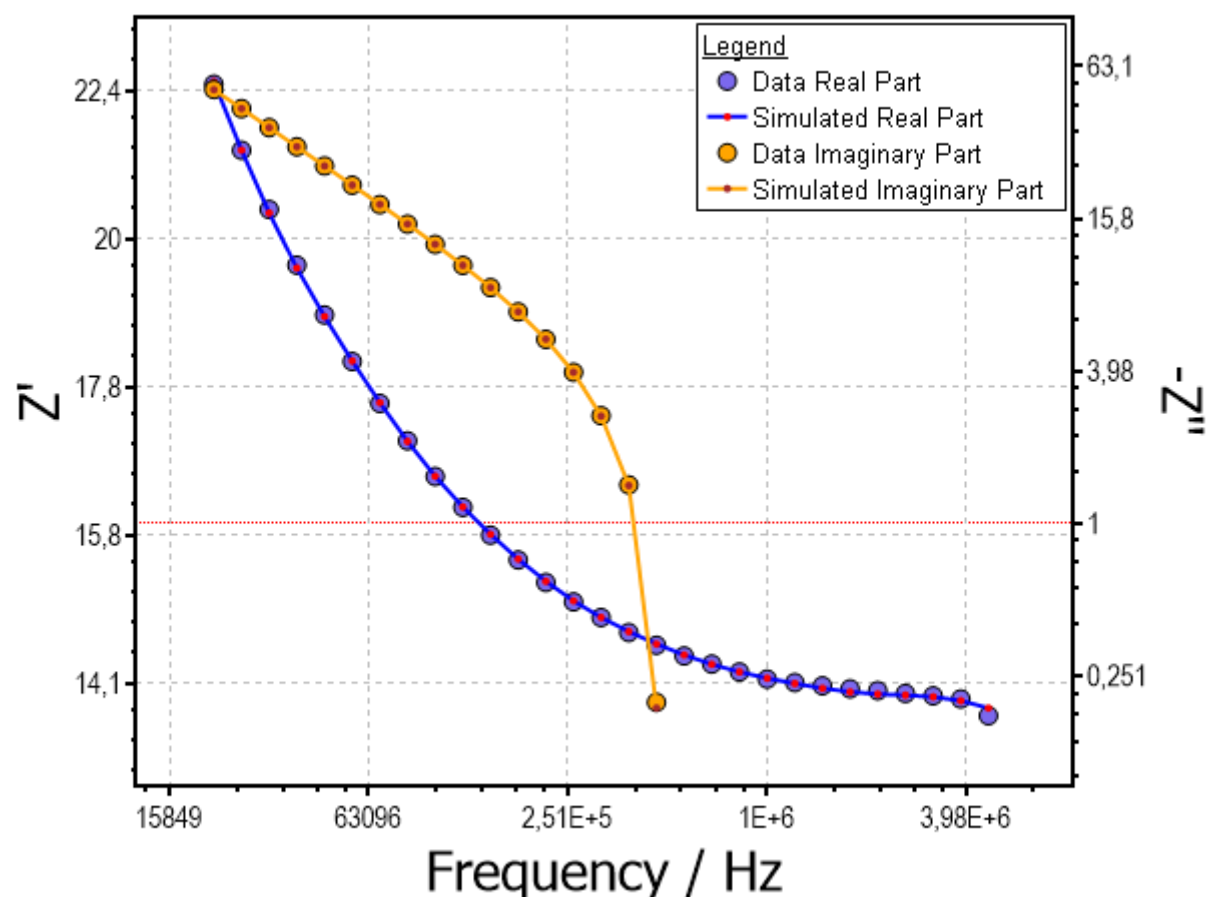

40C

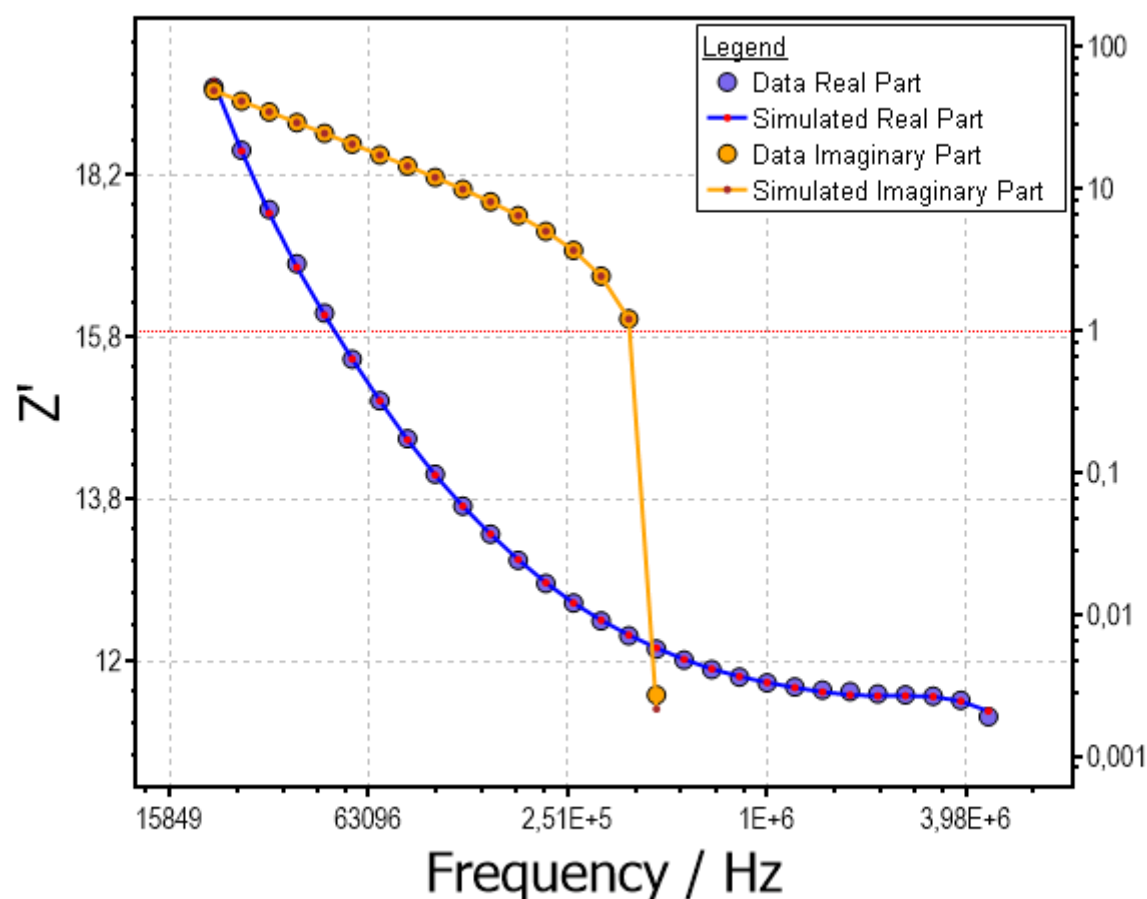

50C

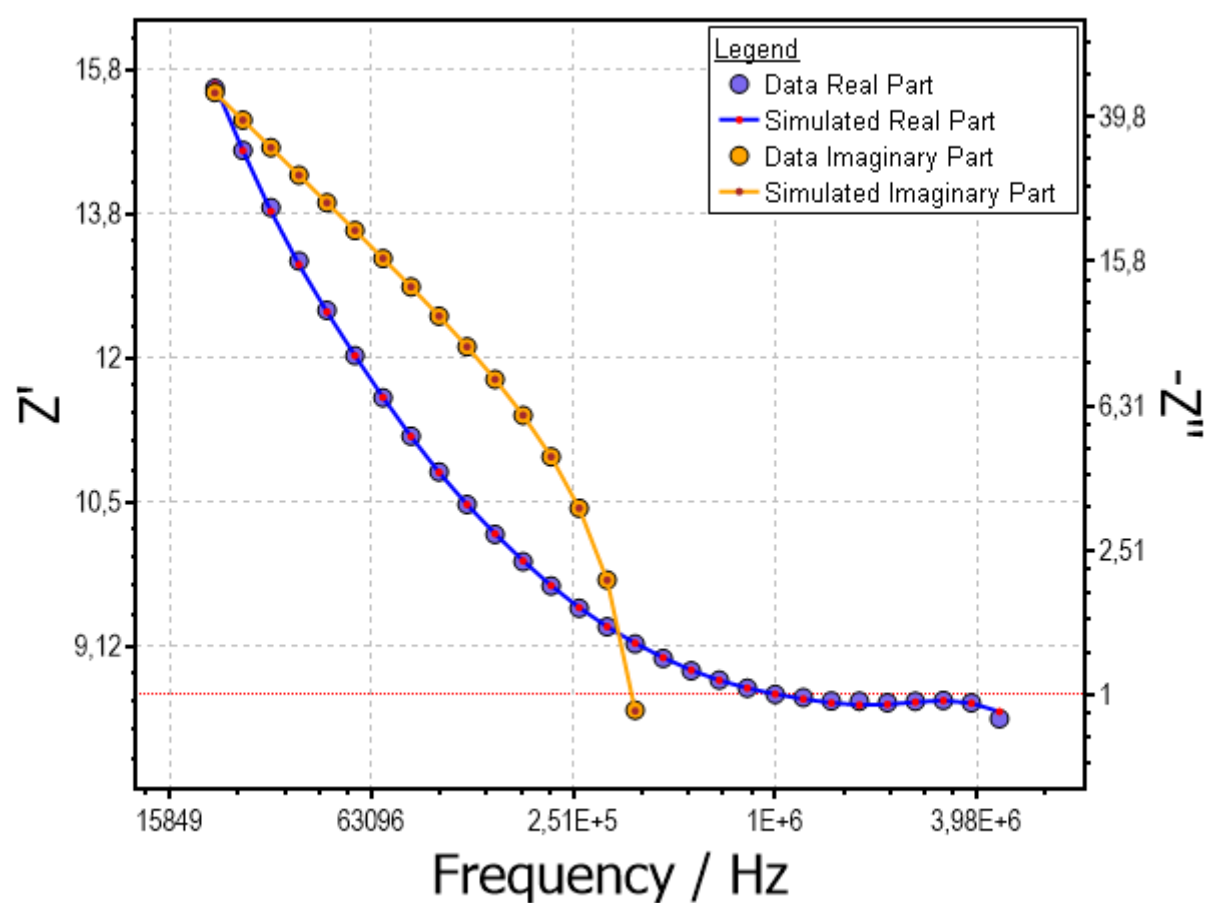

60C

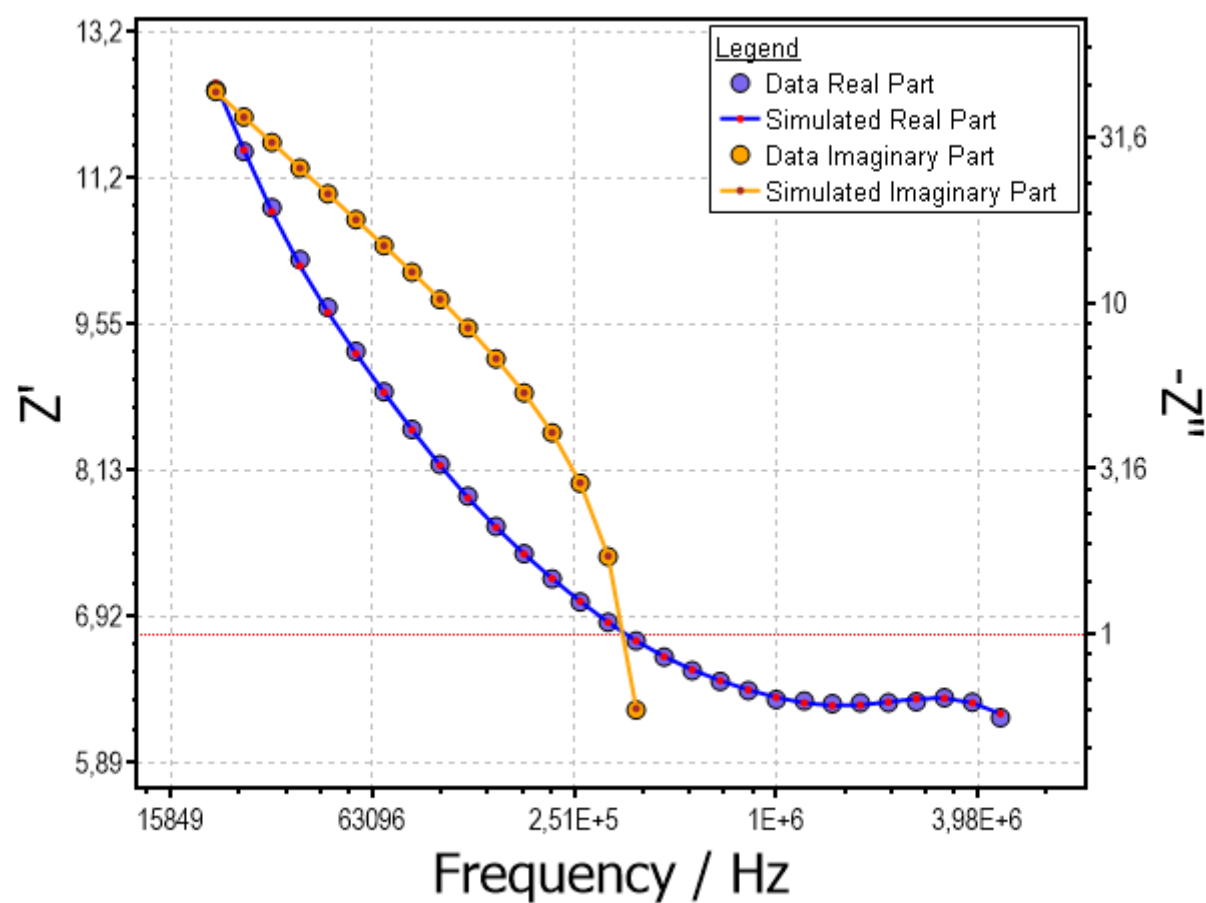

70C

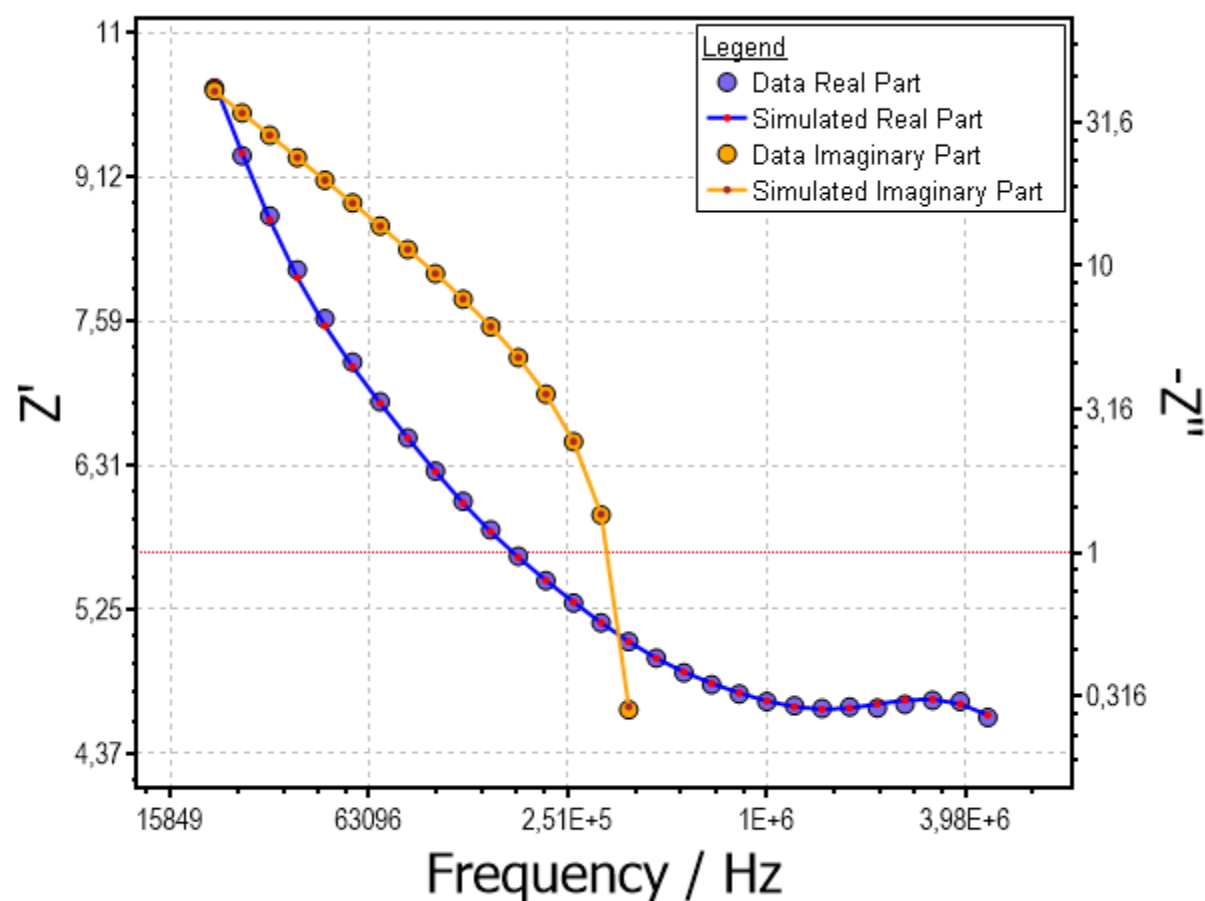

80C

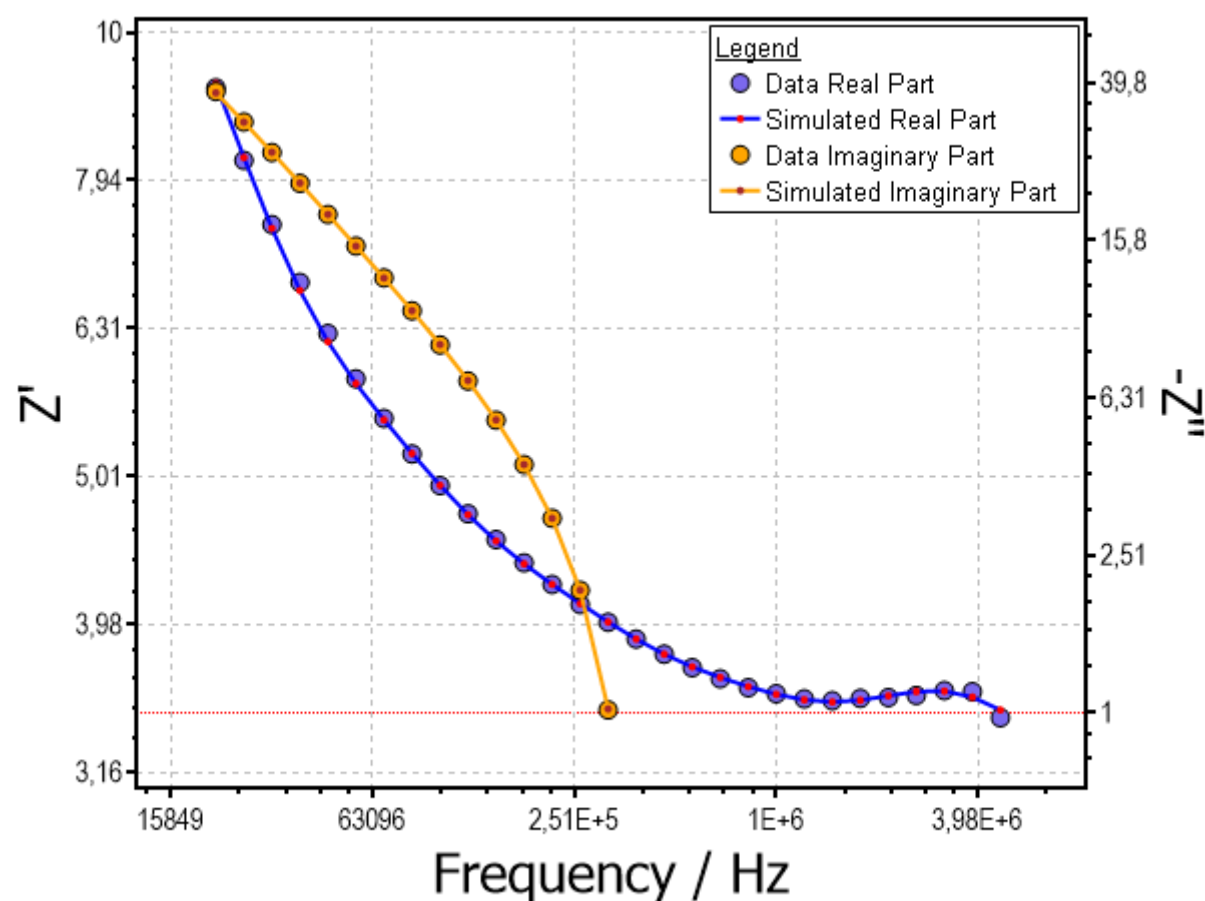

90C

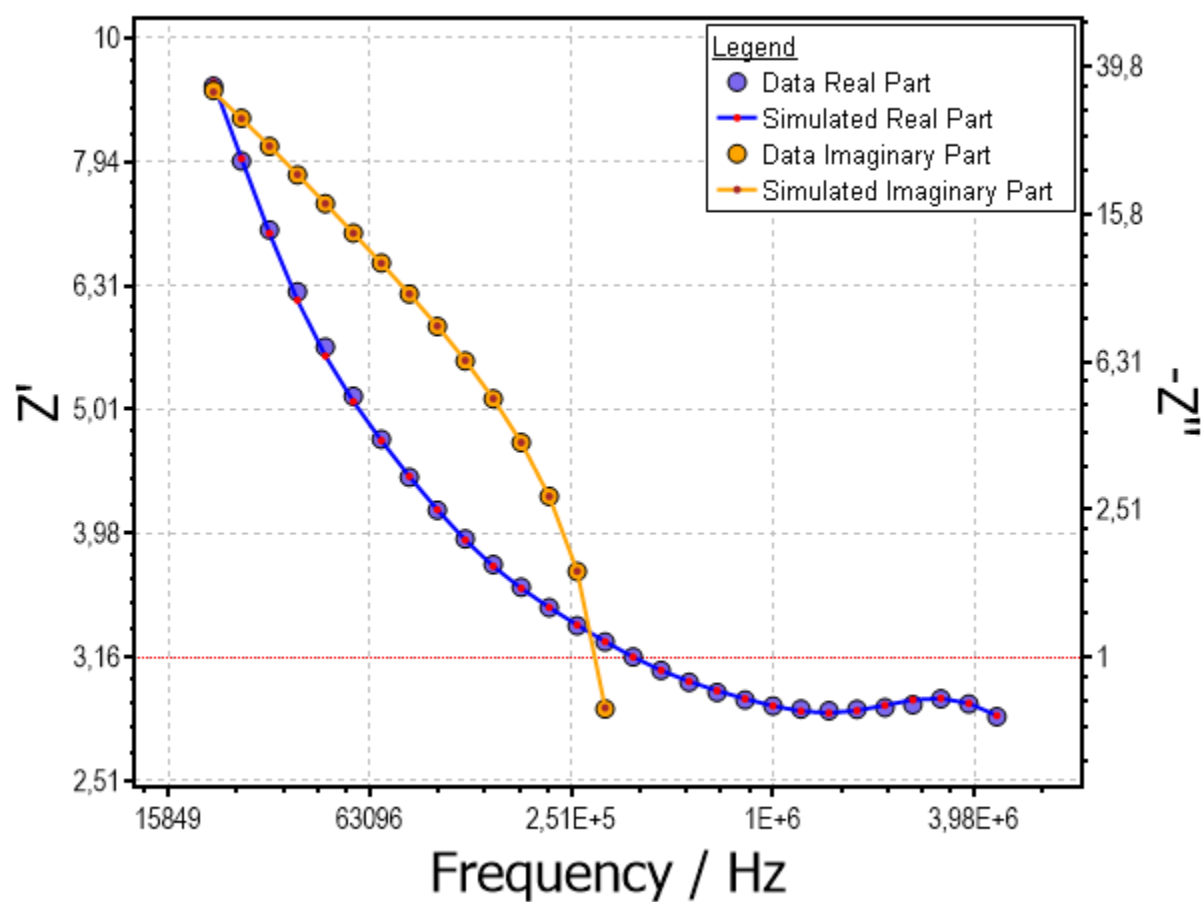

Supplement: Supplementary file 10 — jp2c07910_si_010.pdf [file jp2c07910_si_010.pdf]

30C

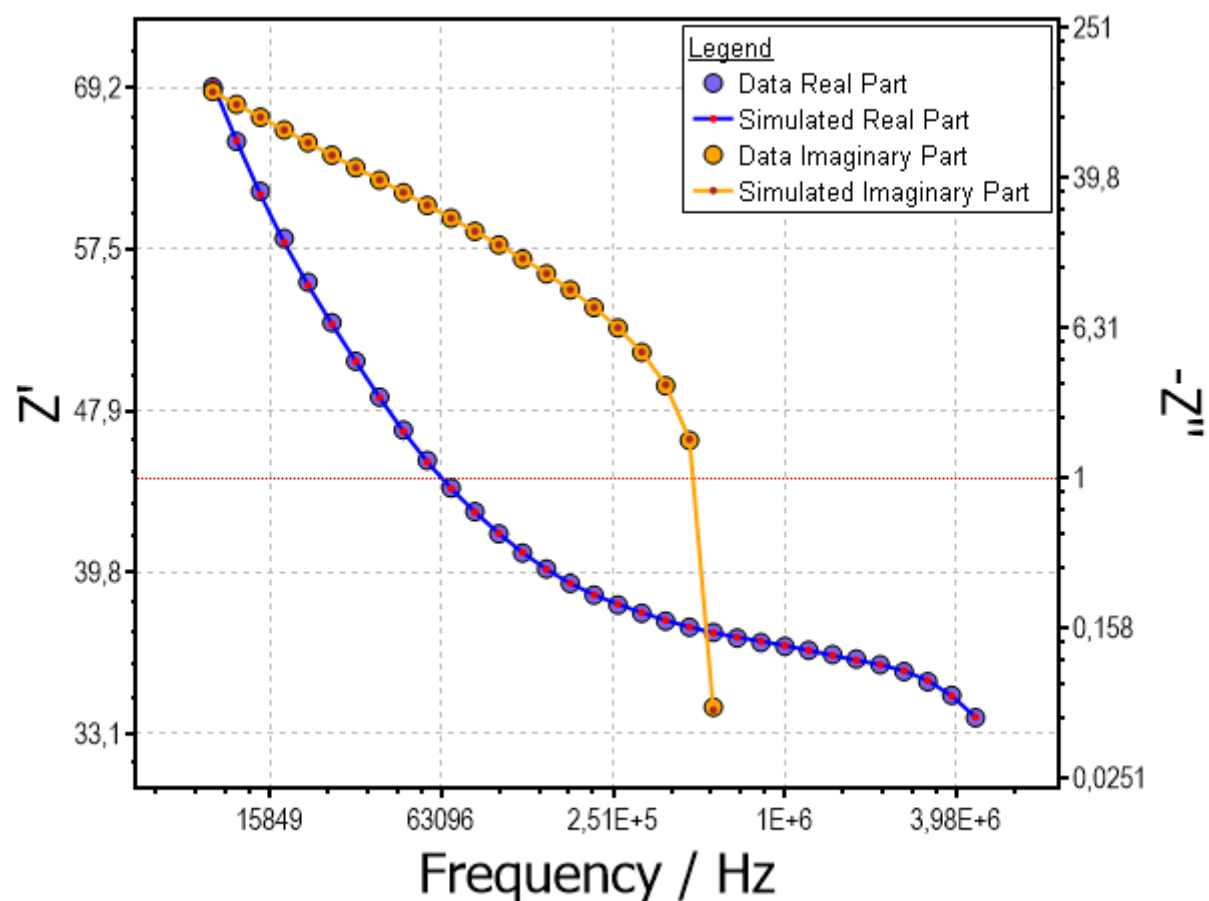

40C

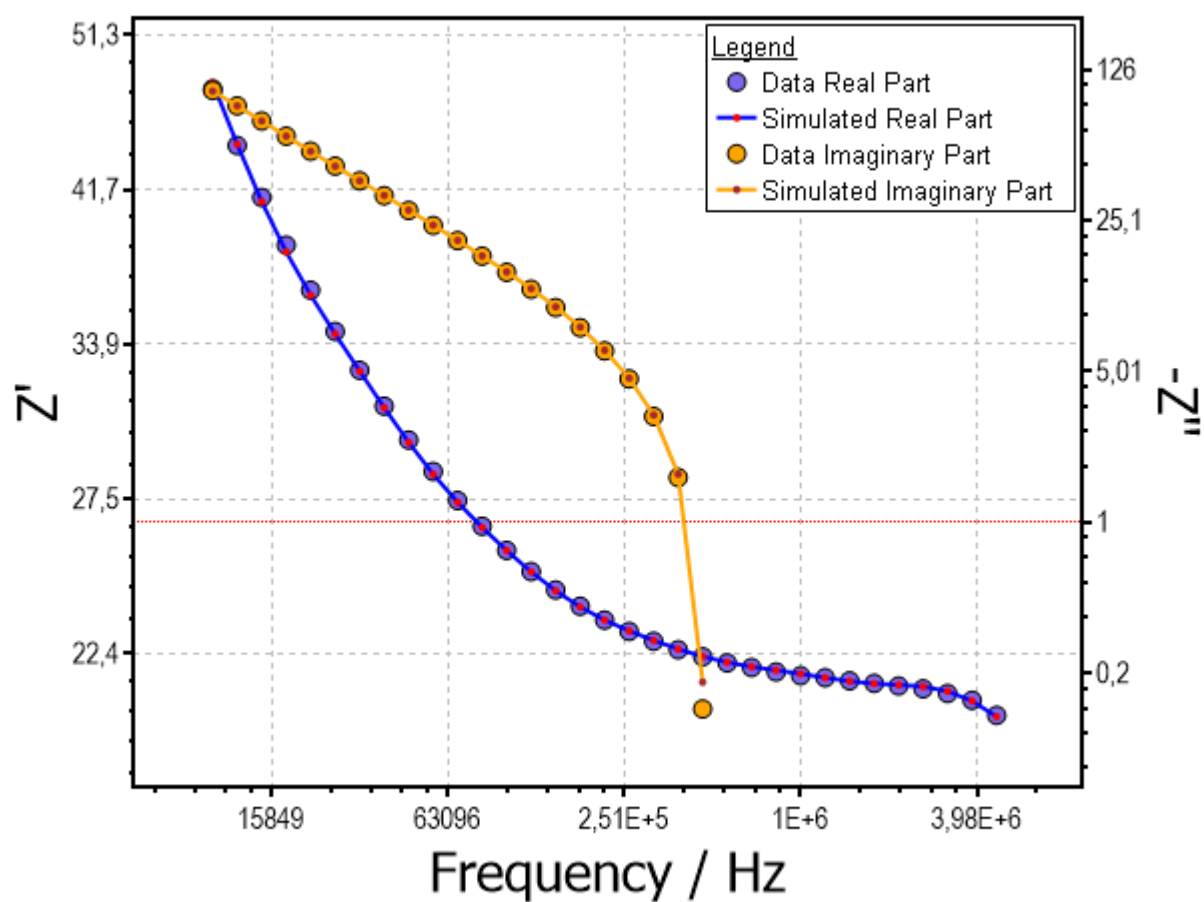

50C

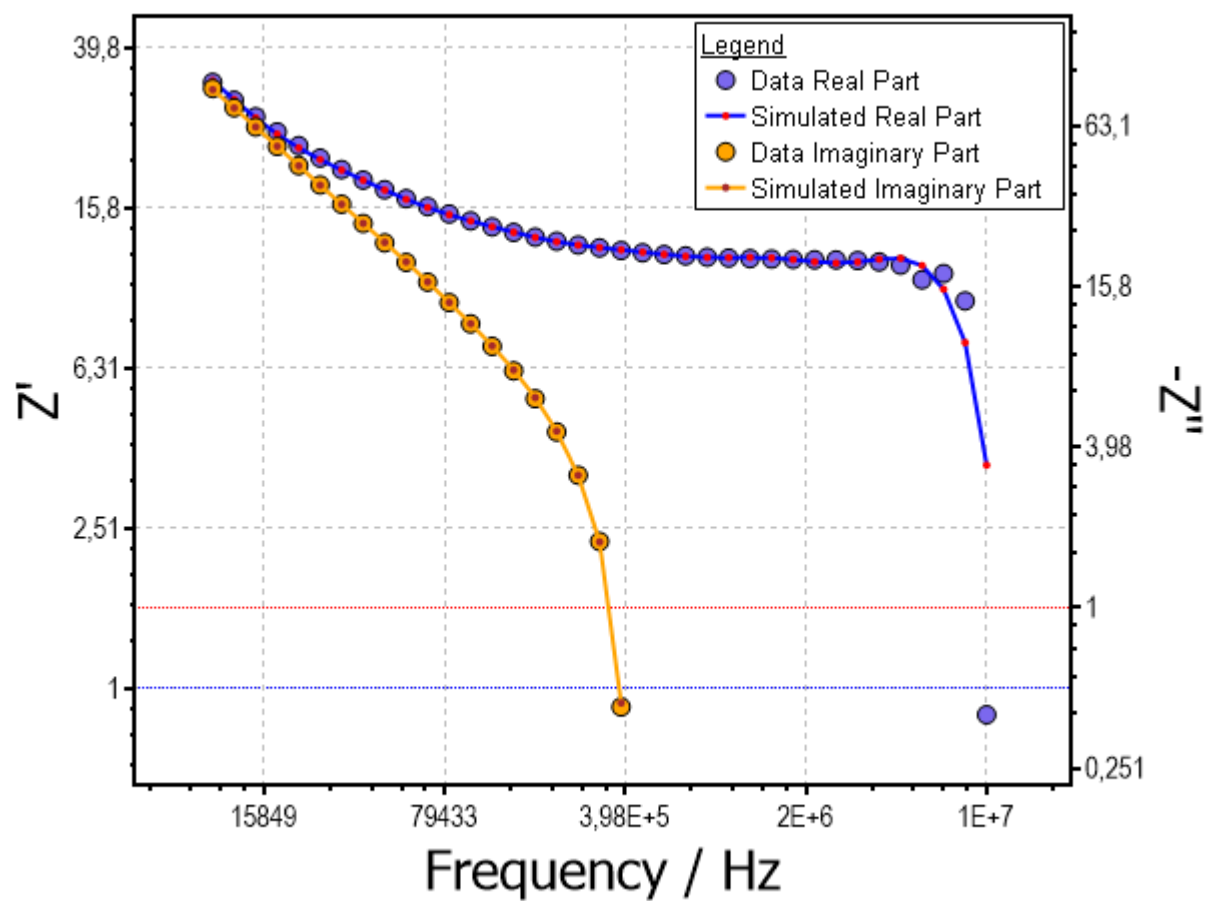

60C

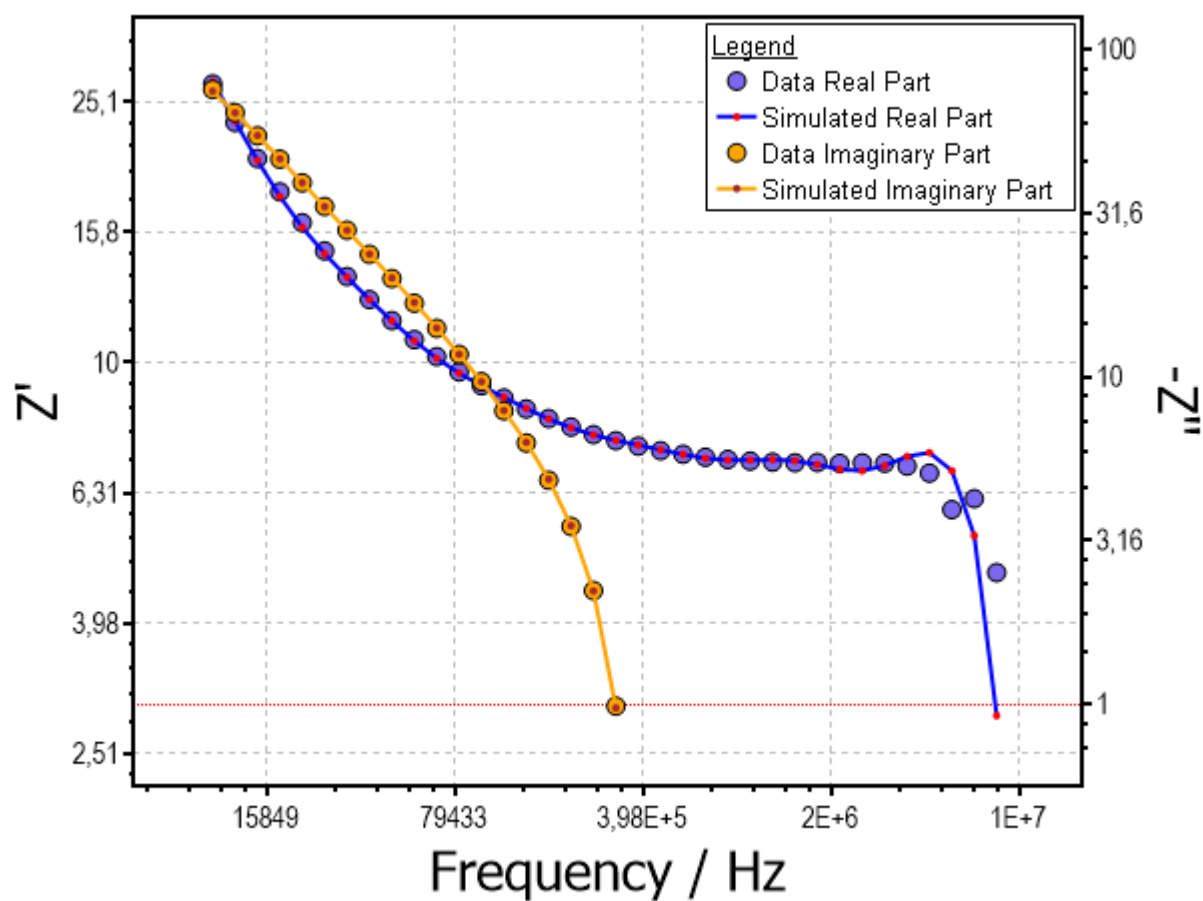

70C

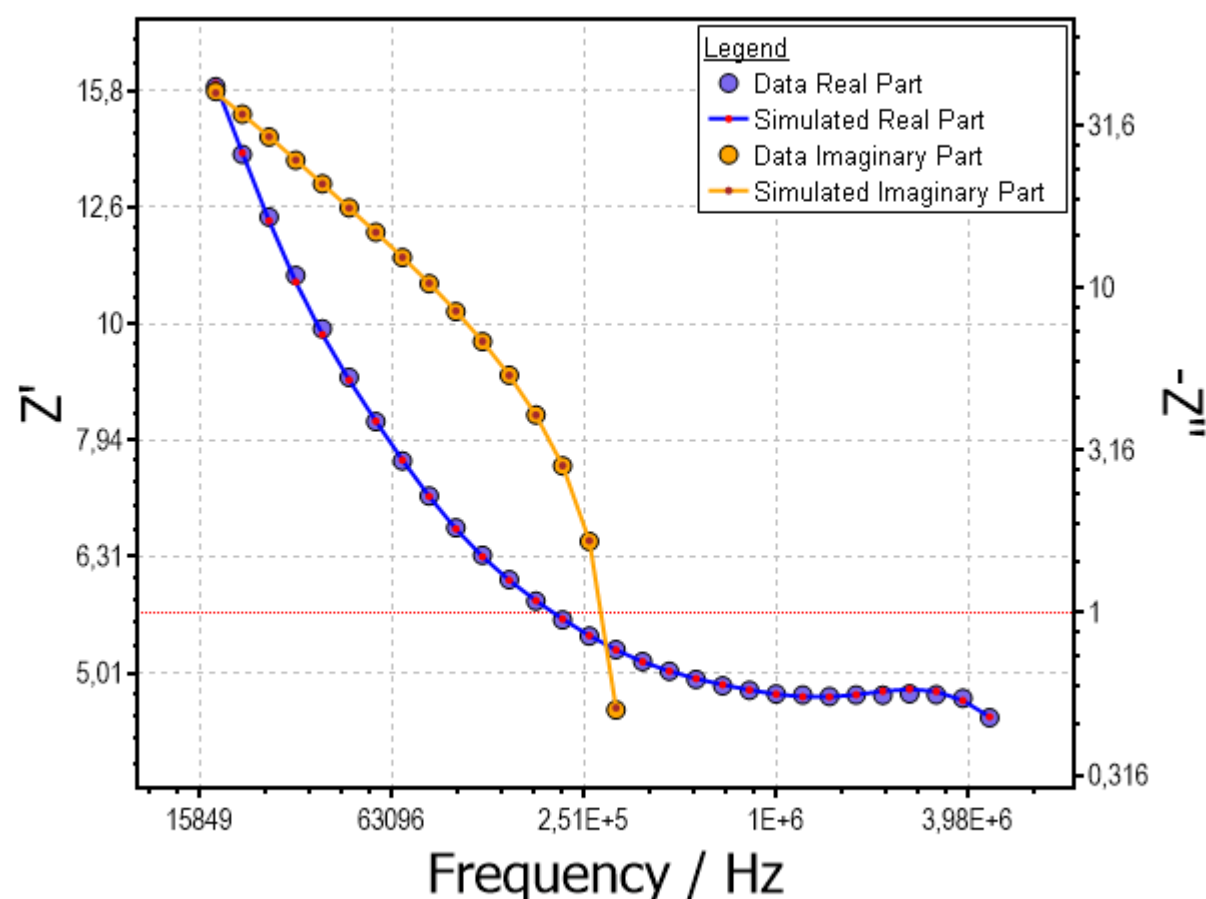

80C

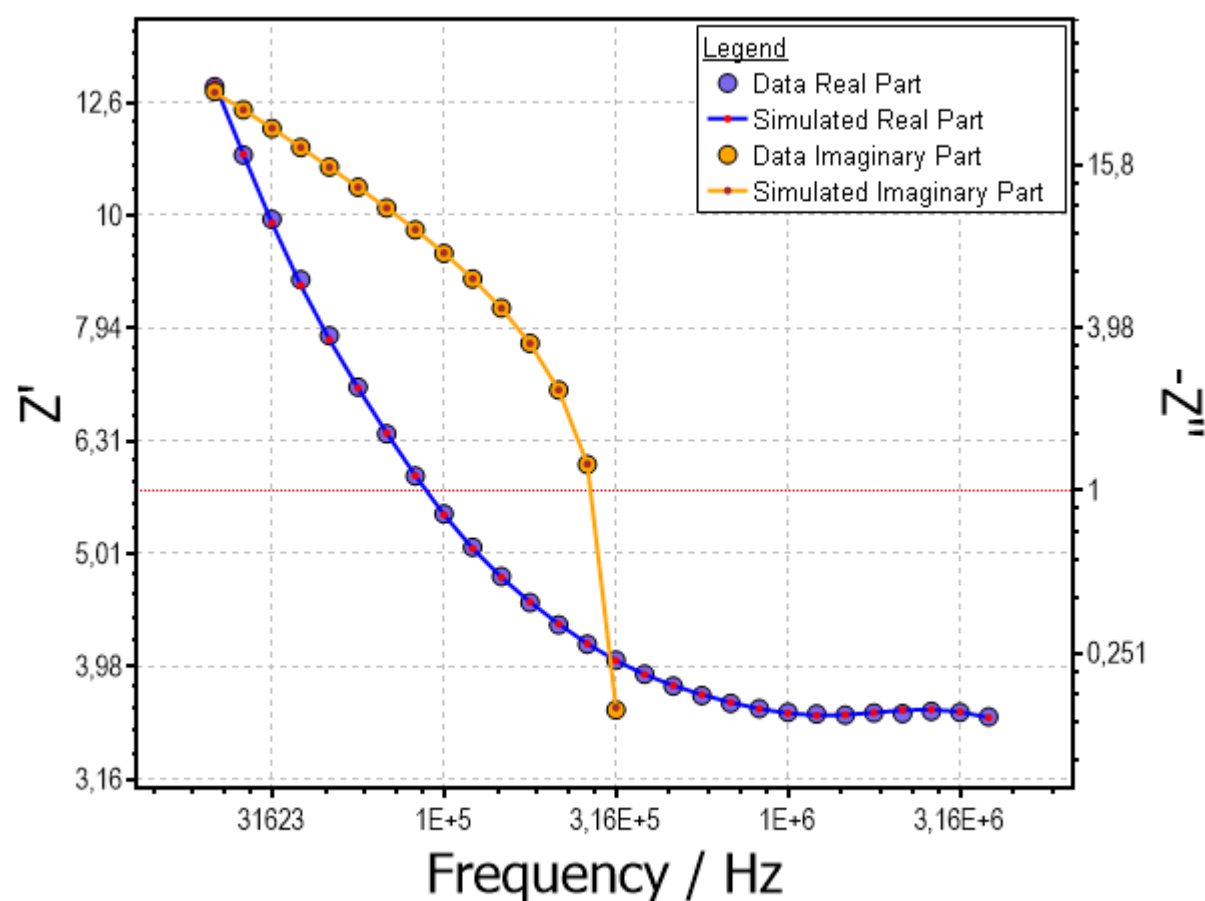

90C

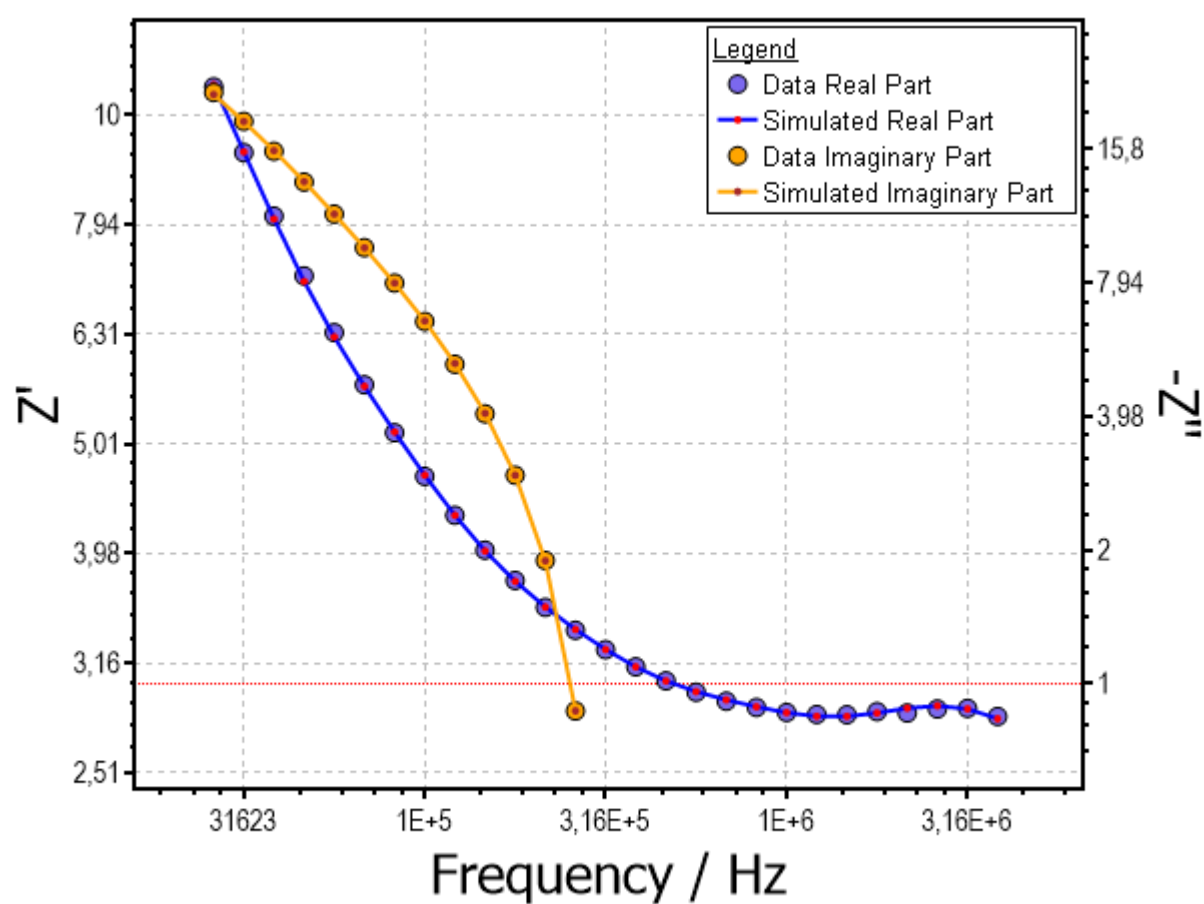

Supplement: Supplementary file 11 — jp2c07910_si_011.pdf [file jp2c07910_si_011.pdf]
